# Diagnostic Accuracy of Clinical Findings for Takayasu Arteritis: A Rapid Review and Meta-Analysis

**DOI:** 10.1155/ijvm/6092362

**Published:** 2025-09-09

**Authors:** Loris Azoyan, Matthieu Bonjour, Olivier Steichen

**Affiliations:** ^1^Sentinelles Network, Pierre Louis Institute of Epidemiology and Public Health (IPLESP), Sorbonne University, INSERM, Paris, France; ^2^Department of Internal Medicine, Tenon Hospital, Greater Paris University Hospitals (AP-HP), Paris, France

**Keywords:** diagnosis, physical examination, systematic review, Takayasu arteritis

## Abstract

**Objective:** Diagnosis of Takayasu arteritis (TA) is based on a combination of demographic, clinical, biological, and imaging data, but the diagnostic value of each clinical sign remains undetermined. The objective of this rapid review and meta-analyses was to estimate the diagnostic accuracy of these clinical signs.

**Methods:** Eligible studies compared the initial clinical presentation of TA with appropriate controls. The diagnostic reference standard had to be specified. We searched PubMed, Embase, and Google Scholar until May 17, 2024. We assessed bias using the QUADAS-2 tool. We performed meta-analyses using a bivariate random effects model for sensitivity and specificity and a sampling-based approach for positive and negative likelihood ratios (PLR, NLR).

**Results:** Of 15 studies included, 13 were case-control. All studies had a high risk of bias. Overall, 1980 patients with TA were compared to 3129 controls, with the majority having another vasculitis, mostly giant cell arteritis (GCA). Among 29 signs, the most suggestive of TA were vascular signs: blood pressure asymmetry (PLR 9.53, 95% CI 3.43–21.9), vascular bruits (9.0, 2.94–22.4), decrease or absent pulse (8.15, 2.35–22.2), and carotid artery with decreased pulse or tenderness (7.23, 3.64–12.5). Compared to GCA only, several signs reduced the likelihood of TA: headache (0.51, 0.25–0.86), jaw claudication (0.15, 0.05–0.35), polymyalgia rheumatica (0.07, 0.01–0.48), and scalp tenderness (0.04, 0.01–0.30).

**Conclusion:** This review highlights the most useful signs for suspecting the disease when compared to other vasculitis and mimics. This will assist clinicians in estimating the likelihood of TA and guiding investigations.

## 1. Introduction

Takayasu arteritis is a chronic vasculitis that mainly affects the aorta and its primary branches, thus classified as large-vessel vasculitis according to the Chapel Hill classification [1]. Patients most frequently diagnosed with Takayasu arteritis are women aged between 20 and 40 years, from South America, the Mediterranean region, Southeast Asia, and Japan [2, 3]. The onset of Takayasu arteritis is most often subacute. Patients may present systemic and vascular signs. Systemic signs such as headache, fever, arthralgia, and myalgia are neither specific nor consistently present. The diagnosis is easier once vascular signs or complications have occurred [4]. The prognosis of Takayasu arteritis is determined by the cardiovascular complications which are the main cause of death [5]. Treatment is based on systemic glucocorticoids, possibly combined with conventional or biologic immunosuppressants [6, 7]. Treatment in the early phase of disease may improve the prognosis [8].

It is therefore important to make the diagnosis of Takayasu arteritis as early as possible. To date, the diagnosis is based on a combination of demographic, clinical, biological, and imaging findings. There is no perfectly sensitive or perfectly specific sign of Takayasu arteritis. It would be useful to know the precise diagnostic value of symptoms and physical signs to guide the diagnostic process and the order of additional investigations [9, 10]. The differential diagnosis remains broad with other large-vessel vasculitis (e.g., giant cell arteritis, Behçet's disease, and infectious aortitis) as well as traditionally noninflammatory vascular diseases, such as severe atherosclerosis or congenital arterial malformations, which may occasionally present with mild inflammatory signs. In particular, in patients aged between 40 and 50 with signs of large-vessel vasculitis, diagnosis between late-onset Takayasu arteritis and early-onset giant cell arteritis can be difficult.

We performed a rapid review and meta-analysis to provide summary estimates of the sensitivity, specificity, and positive likelihood ratio (PLR) and negative likelihood ratio (NLR) for reported clinical features. Relevant references were identified using a broad search strategy referring to diagnosis and Takayasu arteritis across PubMed accessed databases, Embase, and Google Scholar from the date of inception to May 17, 2024.

## 2. Methods

### 2.1. Study Design and Protocol

We conducted a rapid review and meta-analysis and reported it in accordance with the Preferred Reporting Items for a Systematic Review and Meta-analysis of Diagnostic Test Accuracy Studies (PRISMA-DTA) statement [11] (Supporting Information 1: Table S1). Rapid or restricted reviews streamline traditional systematic review constraints to simplify the process without jeopardizing validity [12, 13]. The constraints relaxed in this review are a more focused search strategy, restricted to articles in French or English, and data extracted by a single researcher. A predefined protocol was registered in PROSPERO (CRD42023389888) and is available online [14]. Due to the lack of proper diagnostic studies, the protocol was amended to include case-control studies.

### 2.2. Eligibility Criteria

The initial intent was to include diagnostic studies of a consecutive or random sample of patients suspected of having Takayasu arteritis that compared the initial clinical presentation depending on the final diagnosis (Takayasu arteritis or other). Due to the limited number of such studies, the protocol was amended to include case-control studies if controls were deemed appropriate (e.g., other vasculitis and noninflammatory vascular diseases) and the other criteria were respected. We have defined case-control studies as studies in which groups of patients with and without the target condition are sampled from two distinct source populations [15]. In these studies, the initial suspicion of Takayasu disease was not mandatory for the control group. Diagnostic case-control studies are sometimes referred to as cross-sectional studies or two-gate diagnostic studies. For convenience, we will refer to them as case-control studies in this review. The reference standard for making the diagnosis had to be explicit (e.g., the 2022 American College of Rheumatology [ACR]/European Alliance of Associations for Rheumatology [EULAR] classification criteria [16] or expert consensus). Studies had to include a minimum number of 10 patients per group undergoing a structured clinical examination, with the same definition of clinical findings irrespective of the final diagnosis. Eligible studies had to provide specificity, sensitivity, or likelihood ratios (LRs) for clinical findings in Takayasu arteritis or provide sufficient data to calculate them (i.e., provide a 2 × 2 table of true positive, false positive, true negative, and false negative cases).

### 2.3. Information Sources and Search

We searched the following electronic bibliographic databases: PubMed accessed databases (MEDLINE and PMC), Google Scholar, and Embase. The PubMed search strategy has been reviewed using the Peer Review of Electronic Search Strategies: 2015 Guideline Statement [17]. This search strategy was adapted to other databases. The search strategy included terms referring to diagnosis and Takayasu arteritis. The full search strategy for PubMed accessed databases and Embase is detailed in Supporting Information 1: Tables S2 and S3. References of included primary studies and previous relevant reviews were screened to find additional eligible studies. A metaresearch study has shown that Google Scholar can be an effective tool for identifying gray literature [18]. As recommended by the authors, the search was limited to the first 300 results to balance efficiency and comprehensiveness [18]. Searches were limited to articles written in English and French. Databases were searched from inception date to January 3, 2023, and again on May 17, 2024.

### 2.4. Study Selection and Data Collection

Title and abstracts of all references retrieved using the search strategy were independently screened by two reviewers (L.A. and M.B.) to exclude clearly irrelevant references. The full text of all remaining references was then retrieved and independently assessed by the same reviewers to ascertain inclusion and exclusion criteria. A standardized, prepiloted form was used by one investigator (L.A.) to extract data for the assessment of study quality and evidence synthesis for each symptom or physical sign. Data extraction was checked by another investigator (M.B.). Extracted information included all elements suggested in the checklist of the PRISMA-DTA statement [11]. Disagreements were solved by consensus at each step (title and abstract screening, full text screening, and data extraction).

### 2.5. Risk of Bias and Applicability

Risk of bias and applicability concerns were assessed with an adaptation of the second version of the Quality Assessment of Diagnostic Accuracy Studies (QUADAS-2) tool [19]. QUADAS-2 includes four domains: patient selection, index test (i.e., the evaluated clinical sign), reference standard, and flow and timing. Each domain is evaluated for risk of bias, and the first three domains are also evaluated for applicability issues. Case-control studies were considered to have a high risk of bias due to the retrospective nature of their design, as included patients were not all initially suspected of having Takayasu arteritis. The reference standard was considered to be at high risk of bias due to the incorporation bias (i.e., clinical examination data are necessary for diagnosis) [20]. Most of the studies were deemed to be at high risk of bias for the flow and timing domain because most patients were not evaluated with the same reference standard to rule in Takayasu arteritis or an alternative diagnosis. The studies using expert opinion as a reference standard were considered to be of low concern regarding their applicability, as they were close to usual practice. Two investigators (L.A. and M.B.) independently performed these evaluations. Disagreements were resolved by consensus.

### 2.6. Diagnostic Accuracy Measures, Synthesis of Results, and Meta-Analysis

We performed a qualitative synthesis of the settings, methods, and findings of included studies. Synthesis of results was conducted according to the Cochrane Handbook for Systematic Reviews of Diagnostic Accuracy Studies [21]. We constructed paired forest plots of sensitivity and specificity for each symptom or physical sign. The paired results for sensitivity and specificity were plotted as points in a summary receiver operating characteristics (SROC) space. A bivariate random effects model was fitted to produce estimates of pooled specificity and sensitivity [22]. Pooled PLR and NLR were estimated using a sampling-based approach. They were then compared to calculated PLR and NLR calculated from the pooled sensitivity and specificity to check for consistency [23]. When only two studies were available for a given sign, we used univariate random effects logistic regression to independently compute pooled sensitivity, specificity, PLR, and NLR [24]. Discrepancies between bivariate and univariate random effects models were systematically assessed when the number of studies was small (i.e., four or less). Heterogeneity was not formally assessed due to the relatively small number of studies but was visually explored on the forest plots and the SROC curves. We did not evaluate publication bias given the number of index tests assessed and the lack of suitable methods for meta-analysis of diagnostic accuracy studies [25]. We also evaluated the diagnostic properties of clinical signs in studies comparing only Takayasu arteritis and giant cell arteritis, both large-vessel vasculitis. Analyses were performed in R Version 4.2.0 using the mada package [26–28].

## 3. Results

### 3.1. Study Selection and Study Characteristics

**Figure 1:**
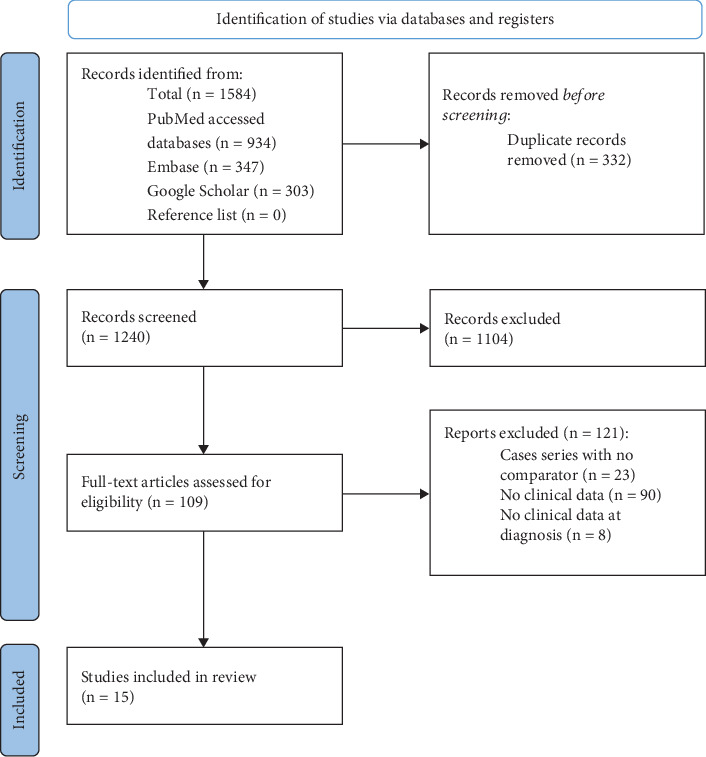
PRISMA flow chart of the study selection process.

Among the 1572 records screened, 15 studies fulfilled the selection criteria (Figure 1). The 15 included studies are described in Table 1. Among them, 13 are case-control studies and two are cohort studies (one prospective and one retrospective). Six used giant cell arteritis as a comparator, while the other nine used a variety of inflammatory and noninflammatory diseases. For all studies, the reference standard included vascular imaging. Eight of them used the ACR 1990 criteria [30], most often modified to include patients under 50 years (rather than under 40 years) and noninvasive imaging procedures. The reference standard of the other studies was based on the opinion of one or more experts. Only the study by Chugh et al. and by Meng et al. specified the reason to suspect Takayasu arteritis as they, respectively, described a cohort of patients with renovascular hypertension and with mid-aortic syndrome [31, 42]. A total of 1980 patients with Takayasu arteritis were compared to 3129 subjects without Takayasu arteritis. Age and sex ratios of cases with Takayasu arteritis and comparators often differed largely.

### 3.2. Risk of Bias and Concerns for Applicability

**Figure 2:**
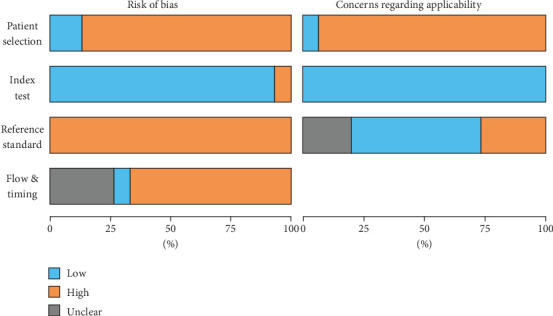
Unweighted proportions of included studies with low, high, or unclear risk of bias or concerns regarding applicability for each domain of the QUADAS-2 tool.

The summary bar plot of the QUADAS-2 evaluation is drawn in Figure 2, and details for each study are available in Supporting Information 1: Table S4. Among the 15 studies, 13 of them were deemed at high risk of bias for the patient selection domain due to their design. The reference standard was considered at high risk of bias for all studies due to an incorporation bias [20]. Most of the studies were deemed at high risk of bias for the flow and timing domain because patients were not evaluated with the same reference standard to rule in Takayasu arteritis or another diagnosis. The index test was considered at low risk of bias for 14 of the studies. A potential selection bias compromised the applicability of all included studies, either due to the case-control design or to the specific causes for suspecting Takayasu arteritis (renovascular hypertension or mid-aortic syndrome).

### 3.3. Results of Individual Studies and Synthesis

**Figure 3:**
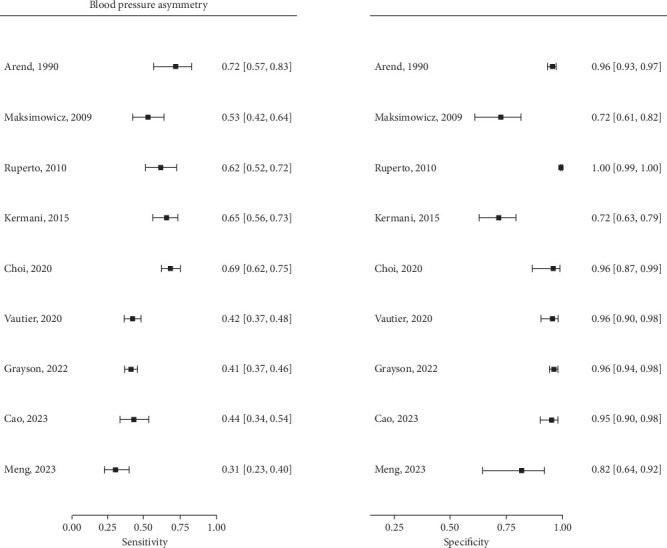
Forest plots of sensitivity and specificity for blood pressure asymmetry.

**Figure 4:**
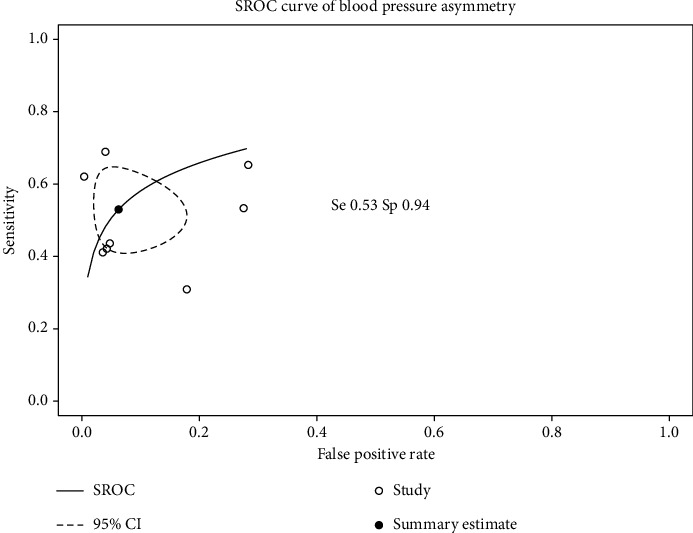
Summary receiver operating characteristics (SROC) curve for blood pressure asymmetry. False positive rate is equal to 1 minus specificity. Summary estimates of sensitivity (Se) and specificity (Sp) are indicated on the plot. CI, confidence intervals.

Forest plots of sensitivity and specificity for each symptom and physical sign are drawn in Supporting Information 2: Figure S1. As an example, the forest plots for the nine studies evaluating blood pressure asymmetry are displayed in Figure 3. The specificity of vascular signs is always better than their sensitivity, and both show moderate heterogeneity. SROC curves, the summary estimate obtained with the bivariate model and its 95% confidence interval region, are plotted in Supporting Information 3: Figure S2 and the blood pressure asymmetry example plotted in Figure 4. Summary estimates of sensitivity, specificity, PLR, and NLR for 21 other vascular features and eight nonvascular features are shown in Table 2. The summary estimates of 11 other symptoms or signs that were only evaluated in two studies are available in Supporting Information 1: Table S5. Vascular signs were the most suggestive of Takayasu arteritis, particularly blood pressure asymmetry (PLR 9.53, 95% CI 3.43–21.9; NLR 0.51, 95% CI 0.40–0.63), vascular bruits (PLR 9.0, 95% CI 2.94–22.4; NLR 0.46, 95% CI 0.34–0.59), decrease or absent pulse (PLR 8.15, 95% CI 2.35–22.2; NLR 0.48, 95% CI 0.35–0.62), and carotid artery with decreased pulse or tenderness (PLR 7.23, 95% CI 3.64–12.5; NLR 0.75, 95% CI 0.56–0.89). Vascular signs involving the carotids or the arms were generally more suggestive than those involving the legs. No systemic signs could distinguish patients with or without Takayasu arteritis. According to the visual assessment, studies showed some degree of heterogeneity for all clinical signs.

### 3.4. Analysis Restricted to Studies Using Giant Cell Arteritis as a Comparator

As a sensitivity analysis, we evaluated 23 clinical signs in studies comparing only Takayasu arteritis and giant cell arteritis. Summary estimates of sensitivity, specificity, PLR, and NLR are shown in Supporting Information 1: Table S6. Of these signs, nine were already reported in the main analysis because all studies compared Takayasu arteritis to giant cell arteritis. The remaining 14 signs showed broadly similar results between the main analysis and the sensitivity analysis for 10 signs. Some signs appeared less specific: decreased or absent pulse (79.5, 95% CI 43.9–95.1 vs. 91.6, 95% CI 74.5–97.6 in the main analysis), arm claudication (78.0, 95% CI 44.5–94.0 vs. 91.3, 95% CI 76.6–97.4), and headache (47.7, 95% CI 30.3–67.4 vs. 63.0, 95% CI 48.1–75.8). The PLR of arm claudication was lower (1.93, 95% CI 0.75–5.2 vs. 5.5, 95% CI 1.1–17.4) and not significant to differentiate the two diseases. The PLR of myocardial infarction was higher (5.86, 95% CI 1.5–16.0 vs. 2.79, 95% CI 0.22–12.7) and in favor of Takayasu arteritis. The PLR of headache was lower (0.51, 95% CI 0.25–0.86 vs. 0.80, 95% CI 0.53–1.21) and in favor of giant cell arteritis. Among the most useful signs, blood pressure asymmetry, decreased or absent pulse, carotid bruit, subclavian arteries bruit, and vascular bruits were in favor of Takayasu arteritis, whereas headache, jaw claudication, arthralgia, myalgia, polymyalgia rheumatica, and scalp tenderness were in favor of giant cell arteritis. Of note, myocardial infarction and stroke or transient ischemic attack were also in favor of Takayasu arteritis despite the younger age of patients.

## 4. Discussion

### 4.1. Main Findings

This rapid review and meta-analysis provides summary estimates of sensitivity, specificity, PLR, and NLR for 29 symptoms and physical signs for the diagnosis of Takayasu arteritis. Although those estimates were obtained from a limited number of studies with a high risk of bias, we were able to identify the most useful signs to distinguish Takayasu arteritis from other vasculitis or mimics. Vascular clinical findings, especially those involving the carotid or arm arteries, had a better diagnostic accuracy than systemic signs. Although none of these signs alone is sufficient to establish the diagnosis, they allow for a more precise assessment of the likelihood of the disease to guide the diagnostic process and the selection and timing of additional investigations. When restricted to studies comparing Takayasu arteritis and giant cell arteritis, diagnostic properties were broadly similar for most of the evaluated signs. The presence of headache, jaw claudication, arthralgia, myalgia, polymyalgia rheumatica, and scalp tenderness was in favor of giant cell arteritis.

### 4.2. Limitations

Our study suffers from several limitations, related both to the review process and to the included studies. As our search strategy was limited to articles written in French or English, other eligible studies may have been overlooked. However, this is only expected to marginally affect the results and conclusion of our review [43].

The small number of included studies and their design limit the significance of the results. None of the included studies was a rigorous diagnostic accuracy study. Such a study would evaluate a population suspected of having Takayasu arteritis and then compare the findings in participants having or not having the disease according to a reference standard. The reason to suspect Takayasu arteritis was not reported in most of the included studies. The majority of them are case-control studies, and the choice of comparators is questionable. Indeed, in most studies, sex and age already discriminate cases with Takayasu arteritis from comparators, given the predominance of giant cell arteritis. In addition, the type of comparators included in the studies may overestimate or underestimate the specificity, PLR, and NLR of each sign [15], as shown by the analysis restricted to patients with giant cell arteritis.

Furthermore, as a significant number of the included studies were used to develop classification criteria, the comparators may include other vasculitis that share few clinical features with Takayasu arteritis, such as small vessel vasculitis [30, 33, 36]. Using classification criteria to differentiate patients also results in a homogeneous population that may not represent the full spectrum of Takayasu arteritis. This issue was better addressed in more recent studies. For example, in the 2022 ACR classification criteria, one-third of the comparators had giant cell arteritis, one-third had vasculitis that could mimic Takayasu arteritis, and one-third had other mimicking noninflammatory diseases [16]. Symptoms and physical signs were not always explicitly and homogeneously defined, which prevented them from being combined in the meta-analyses.

All included studies suffered from incorporation bias: the investigated clinical signs were part of the diagnostic reference standard [20]. This is particularly true for vascular signs, for example, limb claudication, blood pressure asymmetry, diminished pulse, or bruits that were already included in the 1990 ACR criteria [30]. The diagnostic value of these signs may therefore have been overestimated. On the other hand, as the diagnosis is more often made after the appearance of vascular signs, the accuracy of signs that could have occurred earlier, such as fever, weight loss, or arthralgia, may be underestimated [44].

We could not estimate the diagnostic accuracy of less frequent findings that were not reported in the included studies, such as pyoderma gangrenosum, erythema nodosum, or episcleritis. We could not study the diagnostic accuracy of a combination of signs because we had no access to individual data. Finally, we could not perform subgroup or metaregression analysis to evaluate heterogeneity and its causes due to the limited number of studies.

### 4.3. Generalizability, Applicability, and Perspective

Despite these limitations, this review updates the diagnostic value of well-known clinical signs based on more recent studies with broader comparator groups, helping to refine pretest probability assessments. The better diagnostic accuracy of vascular signs is consistent with what has been shown in the studies that have served as a basis for the diagnostic criteria [16, 29, 30, 36, 45].

We have identified relevant signs for an evidence-based approach to the clinical examination for Takayasu arteritis and quantified how they change the clinical probability of the disease. A more accurate assessment of clinical probability can improve the diagnostic process, with timely requests for appropriate additional investigations, particularly imaging tests. However, these diagnostic properties have been established in comparison with other vasculitis or mimics and not in a general clinical context. For example, the specificity of leg claudication established in the review is 90%, but this is when compared to other vasculitis after more frequent causes, such as peripheral artery disease, have been ruled out. These findings are also useful for medical teaching by targeting relevant knowledge and reducing superfluous information for students.

Future diagnostic studies should include patients suspected of having Takayasu arteritis and compare the clinical findings between those who have the disease and those who do not, according to a robust reference standard. These studies would enable a precise estimate of the diagnostic value of clinical findings without suffering from the limits of the studies included in our review. They would also provide more representative data on the spectrum of differential diagnoses encountered in clinical practice, beyond the overrepresentation of giant cell arteritis in existing studies, and contribute to the development of more robust diagnostic criteria.

## 5. Conclusion

This rapid review and meta-analyses outline that vascular signs are the most useful to increase the clinical probability of Takayasu arteritis compared to other vasculitis or mimics. Although our results suffer from a number of limitations, they are a first step toward an evidence-based approach to the clinical examination of this disease.

## Figures and Tables

**Table 1:** Summary of included studies. Age at diagnosis refers to the mean or median, depending on each study. TAK, Takayasu arteritis; NA, not applicable; GCA, giant cell arteritis; PAN, polyarteritis nodosa; GPA, granulomatosis with polyangiitis; EGPA, eosinophilic granulomatosis with polyangiitis; ACR, American College of Rheumatology; LVV, large-vessel vasculitis; AAV, antineutrophil cytoplasmic autoantibody–associated vasculitis; BD, Behçet's disease; CNS, central nervous system.

**Study (year)**	**Design**	**Period**	**Location**	**Reference standard for TAK diagnosis**	**Comparator**	**Number of TAK cases**	**Age at TAK Dx**	**Number of comparator cases**	**Age at comparator Dx**
Ishikawa (1988) [29]	Case-control	1957–1986	Japan	Expert opinion + angiography	6 NA 6 TAK suspicions excluded after angiography	96 (90.6)	32.1	12 (NA)	29.1
Arend et al. (1990) [30]	Case-control	1982–1987	United States, Canada, and Mexico	Agreement between submitting physician and another investigator	214 GCA 118 PAN 129 unspecified 93 HV 85 GPA 85 IgA vasculitis 20 EGPA	63 (85.7)	26.4	744 (54.2)	49.7
Chugh et al. (1992) [31]	Prospective cohort of patients with renovascular hypertension	NA	India	Clinical + angiography	58 FMD 16 atherosclerosis	125 (55.2)	26.8	74 (48.6)	28.3
Maksimowicz-McKinnon et al. (2009) [32]	Case-control	1992–2004	United States	Modified ACR 1990 criteria + mandatory imaging	69 GCA	75 (90.7)	26.0	69 (82.6)	67.0
Ruperto et al. (2010) [33]	Pediatric case-control	NA–2008	International	Diagnosis by center physician + committee decision for random and difficult cases	827 IgA 150 PAN 60 GPA	87 (67.8)	10.4	1037 (55.3)	8.1
Furuta et al. (2015) [34]	Case-control	NA	United Kingdom	ACR 1990 criteria	22 GCA	23 (78.3)	29.2	22 (86.4)	65.8
Kermani et al. (2015) [35]	Case-control	1984–2009	United States	Modified ACR 1990 criteria + mandatory imaging	120 GCA with upper arm involvement	125 (91.2)	30.9	120 (80.0)	67.8
Kong et al. (2015) [36]	Case-control	2009–2014	China	Agreement between two experts	68 inflammatory vasculitis 64 noninflammatory vascular disease	131 (80.9)	36.7	132 (34.8)	57.8
Fukui et al. (2019) [37]	Case-control	2003–2017	Japan	Modified ACR 1990 criteria	20 GCA	25 (96.0)	24.0	20 (60.0)	72.0
Choi et al. (2020) [38]	Case-control	1995–2015	Korea	ACR 1990 criteria	50 BD with arterial involvement	206 (83.5)	43.2	50 (40.0)	46.5
Vautier et al. (2020) [39]	Case-control	NA	France	ACR or Sharma criteria	118 GCA with LVV	299 (86.6)	36.0	118 (70.3)	68.0
Grayson et al. (2022) [16]	Case-control	2011–2017	International	Two reviewers + mandatory imaging	151 GCA 150 other vasculitis 149 mimics of LVV	462 (84.6)	32.3	450 (54.7)	58.6
Boiardi et al. (2023) [40]	Case-control	1996–2016	Italy	ACR 1990 or Sharma criteria ± expert opinion	127 GCA with	59 (91.5)	32.0	127 (72.4)	67.0
Cao et al. (2023) [41]	Case-control	2012–2022	China	Agreement between two experts	108 atherosclerotic stenosis 18 GCA	94 (87.2)	28.0	126 (23.8)	53.0
Meng et al. (2023) [42]	Retrospective cohort of patients with mid aortic syndrome	2010–2019	China	ACR 1990 or Sharma criteria	28 aortic atherosclerosis	110 (80.0)	37.2	28 (35.7)	56.3

**Table 2:** Diagnostic accuracy of symptoms and physical signs. CI, confidence interval; TA, Takayasu arteritis; Se, sensitivity; Sp, specificity; PLR, positive likelihood ratio; NLR, negative likelihood ratio.

**Symptoms and physical signs**	**Number of studies**	**Number of TAK cases**	**Number of comparators**	**Se (95% CI)**	**Sp (95% CI)**	**PLR (95% CI)**	**NLR (95% CI)**
Vascular							
Decreased or absent pulse [29–31, 33, 35, 36, 38–40, 42]	10	1279	2336	57.1 (43.9–69.3)	91.6 (74.5–97.6)	8.15 (2.35–22.2)	0.48 (0.35–0.62)
Vascular bruits [16, 29–31, 33, 36, 38–41]	10	1598	2603	57.9 (44.5–70.2)	92.6 (78.3–97.7)	9.00 (2.94–22.4)	0.46 (0.34–0.59)
Blood pressure asymmetry [16, 30, 32, 33, 35, 38, 39, 41, 42]	9	1497	2473	53.0 (43.2–62.5)	93.8 (85.3–97.5)	9.53 (3.43–21.9)	0.51 (0.40–0.63)
Hypertension [29, 31, 32, 35, 36, 38, 40, 42]	8	877	608	41.1 (30.9–52.2)	58.4 (39.6–75.0)	1.01 (0.78–1.01)	1.03 (0.86–1.29)
Arm claudication [16, 30, 32, 35, 38, 39, 42]	7	1338	1568	32.3 (22.1–47.8)	91.3 (76.6–97.4)	4.35 (1.34–11.2)	0.75 (0.61–0.91)
Leg claudication [16, 30, 32, 35, 38, 39, 42]	7	1337	1568	14.9 (10.2–21.0)	90.1 (83.7–94.1)	1.61 (0.71–3.19)	0.95 (0.85–1.05)
Claudication [30, 31, 33, 36, 40, 41]	6	526	2195	26.2 (15.7–40.3)	93.9 (80.8–98.2)	5.5 (1.1–17.4)	0.79 (0.63–0.98)
Carotidynia/neck pain [29, 36, 38, 40, 42]	5	602	349	10.5 (6.7–16.2)	97.2 (90.1–99.3)	4.54 (1.21–12.3)	0.92 (0.87–0.98)
Abdominal bruit [30, 32, 35, 42]	4	360	765	37.9 (20.4–59.3)	89.1 (69.8–96.7)	4.52 (0.89–14.5)	0.71 (0.44–1.04)
Carotid artery with reduced pulse or tenderness [16, 29, 39, 41]	4	951	706	26.9 (13.5–46.3)	96.2 (93.7–97.7)	7.23 (3.64–12.5)	0.75 (0.56–0.89)
Chest pain [16, 36, 38, 39]	4	1098	750	12.4 (6.0–23.9)	95.3 (87.4–98.3)	2.89 (1.06–6.5)	0.91 (0.82–0.99)
Myocardial infarction [34, 39, 40, 42]	4	491	295	5.5 (2.4–12.5)	96.5 (82.1–99.4)	2.79 (0.22–12.7)	0.99 (0.90–1.16)
Pulse deficit in arm [16, 30, 32, 41]	4	689	1270	58.5 (43.5–72.0)	83.2 (47.3–96.5)	4.75 (1.09–16.3)	0.54 (0.33–0.91)
Amaurosis [32, 36, 40]	3	265	328	6.20 (1.90–18.6)	94.5 (87.7–97.7)	1.57 (0.22–5.51)	0.99 (0.85–1.10)
Aortic valve murmur [29, 30, 34]	3	181	769	18.7 (8.7–35.6)	95.0 (92.2–96.9)	4.25 (1.27–10.1)	0.85 (0.67–0.98)
Blindness^a^ [32, 34, 40]	3	149	218	2.0 (0.4–10.8)	94.1 (81.1–98.3)	0.79 (0.03–4.3)	1.05 (0.92–1.22)
Blurred vision [32, 36, 42]	3	284	196	7.8 (5.1–11.6)	91.2 (64.1–98.4)	1.36 (0.19–5.35)	1.05 (0.92–1.45)
Jaw claudication^a^ [32, 34, 40]	3	149	218	2.7 (0.8–9.2)	79.8 (62.5–90.3)	0.15 (0.05–0.35)	1.24 (1.09–1.5)
Stroke [32, 39, 42]	3	484	215	9.9 (7.5–12.9)	94.0 (85.6–97.6)	1.85 (0.64–4.34)	0.97 (0.91–1.06)
Stroke or transient ischemic attack^a^ [32, 35, 40]	3	231	316	9.4 (6.0–14.2)	95.5 (26.0–97.3)	2.2 (1.08–4.09)	0.95 (0.90–1.0)
Subclavian artery bruit [30, 32, 35]	3	248	711	38.0 (14.0–69.8)	89.8 (62.5–97.9)	5.66 (0.62–23.0)	0.71 (0.33–1.18)
Other							
Headache [30–32, 34–40, 42]	11	1193	1469	28.8 (21.7–37.2)	63.0 (48.1–75.8)	0.80 (0.53–1.21)	1.15 (0.93–1.47)
Fever [32, 33, 35–42]	10	1156	1794	23.4 (15.3–34.2)	76.9 (64.3–84.7)	1.04 (0.65–1.60)	1.00 (0.87–1.16)
Arthralgia [32, 33, 36, 38, 39]	5	766	1373	15.0 (7.3–28.4)	65.9 (43.3–83.1)	0.46 (0.29–0.68)	1.32 (1.09–1.75)
Weight loss [35, 36, 38, 39, 42]	5	817	415	18.7 (11.1–29.7)	81.5 (62.4–92.1)	1.09 (0.52–2.15)	1.01 (0.87–1.25)
Abdominal pain [33, 38, 39]	3	592	1205	13.3 (1.80–56.6)	77.0 (36.0–95.2)	0.78 (0.11–2.55)	1.16 (0.69–1.98)
Faintness [33, 38, 39]	3	592	1205	30.5 (6.4–73.7)	87.4 (40.5–98.6)	2.78 (1.0–7.27)	0.8 (0.5–1.0)
Myalgia [32, 33, 39]	3	461	1134	12.7 (3.2–39.0)	69.7 (54.9–81.4)	0.51 (0.10–1.42)	1.24 (0.86–1.62)
Oral ulcer^b^ [33, 36, 38]	3	392	1186	4.8 (2.1–10.6)	71.0 (4.7–99.2)	0.29 (0.07–1.12)	1.07 (0.91–1.26)

^a^For these signs, all comparators had giant cell arteritis.

^b^PLR and NLR were estimated using a univariate random effect logistic regression to maintain consistency when only a limited number of studies were available.

## Data Availability

Data that support the findings of this study and the R script are openly available in the diagnostic accuracy of clinical findings for Takayasu arteritis at https://osf.io/d29sz/?view_only=564d11fd77b44e98bea15bbcf673d25a.

## Supporting Information

Supporting Information 1 Table S1: PRISMA DTA checklist. Table S2: PubMed accessed databases query using the PubMed query system with language restrictions with the number of hits on January 3, 2023. Table S3: Embase query with the number of hits on January 3, 2023. Table S4: Detailed summary of QUADAS-2 items for each study. Table S5: Diagnostic accuracy of symptoms and physical signs estimated by only two studies. Sensitivity, specificity, PLR, and NLR were estimated using a univariate random effects model, and the 95% confidence interval is indicated between parentheses. TA, Takayasu arteritis; Se, sensitivity; Sp, specificity; PLR, positive likelihood ratio; NLR, negative likelihood ratio; NA, not applicable. ⁣^∗^For these signs, all comparators had giant cell arteritis. Table S6: Diagnostic accuracy of symptoms and physical signs for Takayasu arteritis restricted to studies using giant cell arteritis as a comparator. TA, Takayasu arteritis; GCA, giant cell arteritis; Se, sensitivity; Sp, specificity; PLR, positive likelihood ratio; NLR, negative likelihood ratio; NA, not applicable.

Supporting Information 2 Figure S1: Paired forest plots of sensitivity and specificity for each symptom, physical sign, demographic feature, and complications listed in alphabetical order.

Supporting Information 3 Figure S2: Summary receiver operating characteristics (SROC) curve for each symptom, physical sign, demographic feature, and complications listed in alphabetical order. False positive rate is equal to 1 minus specificity. Sensitivity (Se) and specificity (Sp) of the summary estimates are indicated on each plot. CI, confidence intervals.
