# Supplementary material for: Diagnostic Accuracy of Clinical Findings for Takayasu Arteritis: A Rapid Review and Meta-Analysis
Source: Int J Vasc Med. 2025 Sep 9;2025:6092362. doi: 10.1155/ijvm/6092362 (PMC12440657; doi:10.1155/ijvm/6092362)
Supplement: Supporting Information 1 — Table S1: PRISMA DTA checklist. Table S2: PubMed accessed databases query using the PubMed query system with language restrictions with the number of hits on January 3, 2023. Table S3: Embase query with the number of hits on January 3, 2023. Table S4: Detailed summary of QUADAS-2 items for each study. Table S5: Diagnostic accuracy of symptoms and physical signs estimated by only two studies. Sensitivity, specificity, PLR, and NLR were estimated using a univariate random effects model, and the 95% confidence interval is indicated between parentheses. TA, Takayasu arteritis; Se, sensitivity; Sp, specificity; PLR, positive likelihood ratio; NLR, negative likelihood ratio; NA, not applicable. ⁣∗For these signs, all comparators had giant cell arteritis. Table S6: Diagnostic accuracy of symptoms and physical signs for Takayasu arteritis restricted to studies using giant cell arteritis as a comparator. TA, Takayasu arteritis; GCA, giant cell arteritis; Se, sensitivity; Sp, specificity; PLR, positive likelihood ratio; NLR, negative likelihood ratio; NA, not applicable. [file 6092362.f1.docx]

**Supplementary material**

**Supplementary tables legends:**

**Supplementary table S.1** – PRISMA DTA Checklist

**Supplementary table S.2.** PubMed accessed databases query using PubMed query system with languages restrictions with the number of hits on January 3, 2023.

**Supplementary table S.3.** Embase query with the number of hits on January 3, 2023.

**Supplementary table S.4.** Detailed summary of QUADAS-2 items for each study.

**Supplementary table S.5.** Diagnostic accuracy of symptoms and physical signs estimated by only two studies. Sensitivity, specificity, PLR and NLR were estimated using a univariate random effect model, 95% Confidence interval are indicated between parentheses. TA: Takayasu arteritis, Se: sensitivity, Sp: specificity, PLR: positive likelihood ratio, NLR: negative likelihood ratio, NA: not applicable. *For these signs, all comparators had giant cell arteritis.

**Supplementary table S.6.** Diagnostic accuracy of symptoms and physical signs for Takayasu arteritis restricted to studies using giant cell arteritis as a comparator. TA: Takayasu arteritis, GCA: giant cell arteritis, Se: sensitivity, Sp: specificity, PLR: positive likelihood ratio, NLR: negative likelihood ratio, NA: not applicable.

**Supplementary figures legends:**

**Supplementary figure S.1.** Paired forest plots of sensitivity and specificity for each symptom, physical sign, demographic feature and complications listed in alphabetical order.

**Supplementary figure S.2.** Summary receiver operating characteristics (SROC) curve for each symptom, physical sign, demographic feature and complications listed in alphabetical order. False positive rate is equal to 1 minus specificity. Sensitivity (Se) and specificity (Sp) of the summary estimates are indicated on each plot. CI: confidence intervals.

| **Section/topic** | **#** | **PRISMA-DTA Checklist Item** | **Reported on page #** |
| --- | --- | --- | --- |
| **TITLE / ABSTRACT** | | |  |
| Title | 1 | Identify the report as a systematic review (+/- meta-analysis) of diagnostic test accuracy (DTA) studies. | 1 |
| Abstract | 2 | Abstract: See PRISMA-DTA for abstracts. | 2 |
| **INTRODUCTION** | | |  |
| Rationale | 3 | Describe the rationale for the review in the context of what is already known. | 3 |
| Clinical role of index test | D1 | State the scientific and clinical background, including the intended use and clinical role of the index test, and if applicable, the rationale for minimally acceptable test accuracy (or minimum difference in accuracy for comparative design). | 3 |
| Objectives | 4 | Provide an explicit statement of question(s) being addressed in terms of participants, index test(s), and target condition(s). | 3 |
| **METHODS** | | |  |
| Protocol and registration | 5 | Indicate if a review protocol exists, if and where it can be accessed (e.g., Web address), and, if available, provide registration information including registration number. | 3-4 |
| Eligibility criteria | 6 | Specify study characteristics (participants, setting, index test(s), reference standard(s), target condition(s), and study design) and report characteristics (e.g., years considered, language, publication status) used as criteria for eligibility, giving rationale. | 4 |
| Information sources | 7 | Describe all information sources (e.g., databases with dates of coverage, contact with study authors to identify additional studies) in the search and date last searched. | 4-5 |
| Search | 8 | Present full search strategies for all electronic databases and other sources searched, including any limits used, such that they could be repeated. | 4-5, table S2 and table S3 |
| Study selection | 9 | State the process for selecting studies (i.e., screening, eligibility, included in systematic review, and, if applicable, included in the meta-analysis). | 5 |
| Data collection process | 10 | Describe method of data extraction from reports (e.g., piloted forms, independently, in duplicate) and any processes for obtaining and confirming data from investigators. | 5 |
| Definitions for data extraction | 11 | Provide definitions used in data extraction and classifications of target condition(s), index test(s), reference standard(s) and other characteristics (e.g. study design, clinical setting). | 5 |
| Risk of bias and applicability | 12 | Describe methods used for assessing risk of bias in individual studies and concerns regarding the applicability to the review question. | 5-6 |
| Diagnostic accuracy measures | 13 | State the principal diagnostic accuracy measure(s) reported (e.g. sensitivity, specificity) and state the unit of assessment (e.g. per-patient, per-lesion). | 6 |
| Synthesis of results | 14 | Describe methods of handling data, combining results of studies and describing variability between studies. This could include, but is not limited to: a) handling of multiple definitions of target condition. b) handling of multiple thresholds of test positivity, c) handling multiple index test readers, d) handling of indeterminate test results, e) grouping and comparing tests, f) handling of different reference standards | 6 |
| Meta-analysis | D2 | Report the statistical methods used for meta-analyses, if performed. | 6 |
| Additional analyses | 16 | Describe methods of additional analyses (e.g., sensitivity or subgroup analyses, meta-regression), if done, indicating which were pre-specified. | 6 |
| **RESULTS** | | | |
| Study selection | 17 | Provide numbers of studies screened, assessed for eligibility, included in the review (and included in meta-analysis, if applicable) with reasons for exclusions at each stage, ideally with a flow diagram. | 6-7, figure 1 |
| Study characteristics | 18 | For each included study provide citations and present key characteristics including: a) participant characteristics (presentation, prior testing), b) clinical setting, c) study design, d) target condition definition, e) index test, f) reference standard, g) sample size, h) funding sources | 6-7, table 1 |
| Risk of bias and applicability | 19 | Present evaluation of risk of bias and concerns regarding applicability for each study. | 7, figure 2, table S4 |
| Results of individual studies | 20 | For each analysis in each study (e.g. unique combination of index test, reference standard, and positivity threshold) report 2x2 data (TP, FP, FN, TN) with estimates of diagnostic accuracy and confidence intervals, ideally with a forest or receiver operator characteristic (ROC) plot. | 7-8, Figure 3 and 4, figure S1 and S2 |
| Synthesis of results | 21 | Describe test accuracy, including variability; if meta-analysis was done, include results and confidence intervals. | 7-8, table 2 |
| Additional analysis | 23 | Give results of additional analyses, if done (e.g., sensitivity or subgroup analyses, meta-regression; analysis of index test: failure rates, proportion of inconclusive results, adverse events). | 8, table S5 S6 |
| **DISCUSSION** | | | |
| Summary of evidence | 24 | Summarize the main findings including the strength of evidence. | 9 |
| Limitations | 25 | Discuss limitations from included studies (e.g. risk of bias and concerns regarding applicability) and from the review process (e.g. incomplete retrieval of identified research). | 9-10 |
| Conclusions | 26 | Provide a general interpretation of the results in the context of other evidence. Discuss implications for future research and clinical practice (e.g. the intended use and clinical role of the index test). | 11 |
| **FUNDING** | | | |
| Funding | 27 | For the systematic review, describe the sources of funding and other support and the role of the funders. | 1 |

**Supplementary table S.1** – PRISMA DTA Checklist

| **N°** | **Query** | **Hits** |
| --- | --- | --- |
| **#1** | Takayasu arteritis[Majr] | 4 053 |
| **#2** | Diagnosis/Broad[filter] | 6 260 136 |
| **#3** | 1 AND 2 | 2 156 |
| **#4** | "Case Reports" [Publication Type] | 2 308 139 |
| **#5** | 3 NOT 4 | 964 |
| **#6** | Restricted to French and English articles | 840 |

**Supplementary table S.2.**

| **N°** | **Query** | **Hits** |
| --- | --- | --- |
| **#1** | 'aortic arch syndrome'/exp/mj | 6 665 |
| **#2** | 'diagnosis'/exp/mj | 2 199 403 |
| **#3** | 1 AND 2 | 776 |
| **#4** | [english]/lim OR [french]/lim | [37](https://www-embase-com.ezproxy.u-paris.fr/) 054 014 |
| **#5** | 3 AND 4 | 623 |
| **#6** | [humans]/lim | 25 659 666 |
| **#7** | 5 AND 6 | 521 |
| **#8** | 'case report'/de | [2 889 160](https://www-embase-com.ezproxy.u-paris.fr/) |
| **#9** | 7 NOT 9 | 302 |

**Supplementary table S.3.**

| Study | Risk of bias | | | | Concerns regarding applicability | | |
| --- | --- | --- | --- | --- | --- | --- | --- |
|  | Patient selection | Index test | Reference standard | Flow & timing | Patient selection | Index test | Reference standard |
| Ishikawa, 1988 | 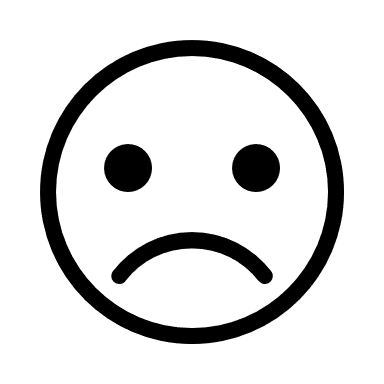 | 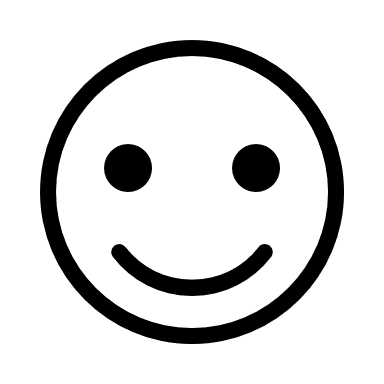 | 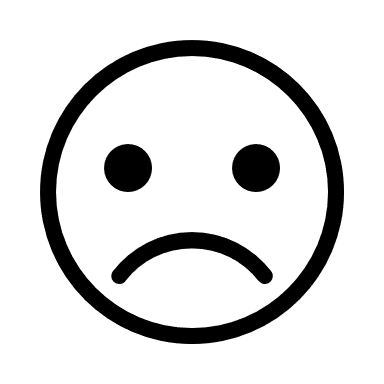 | 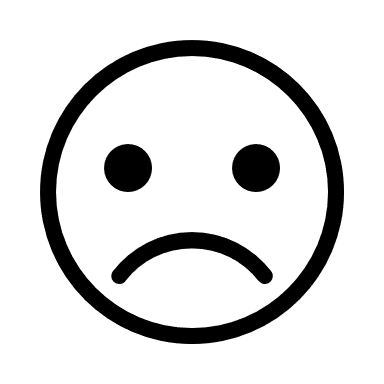 | 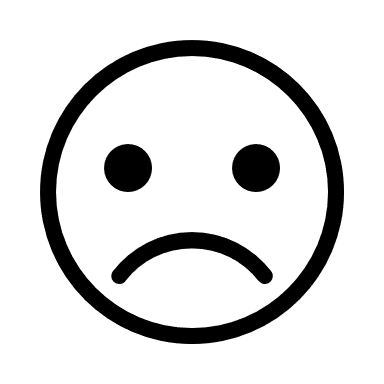 | 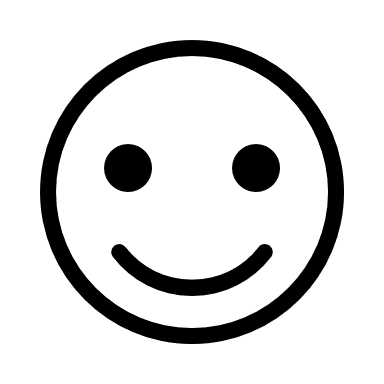 | 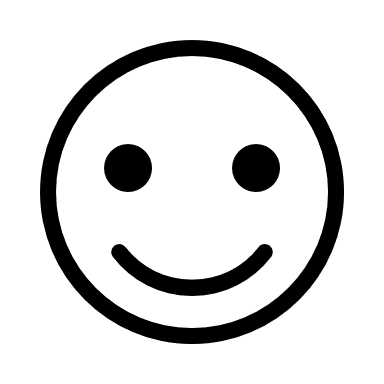 |
| Arend, 1990 | 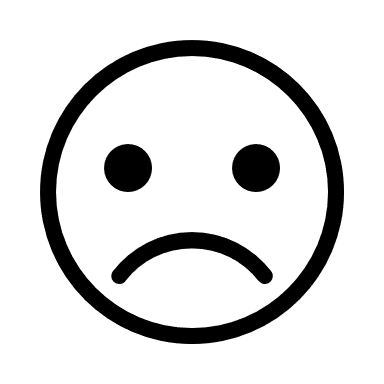 | 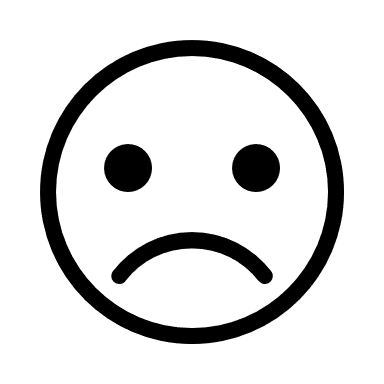 | 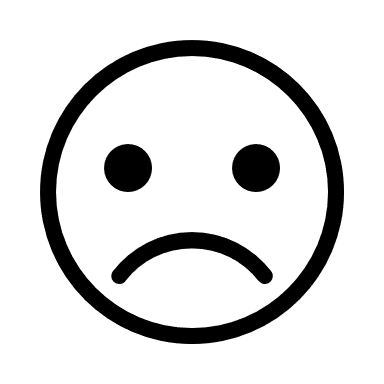 | ? | 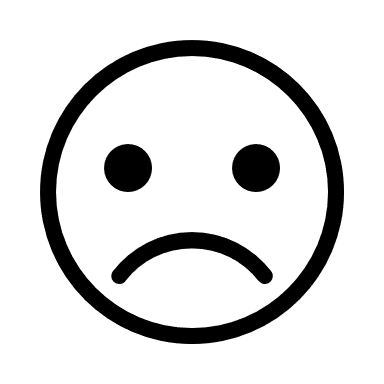 | 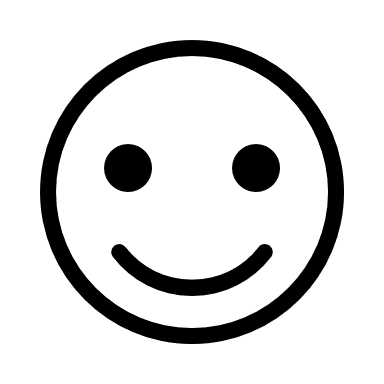 | 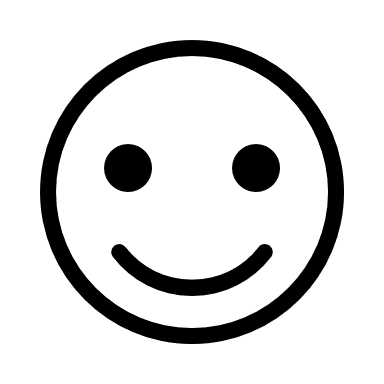 |
| Chugh, 1992 | 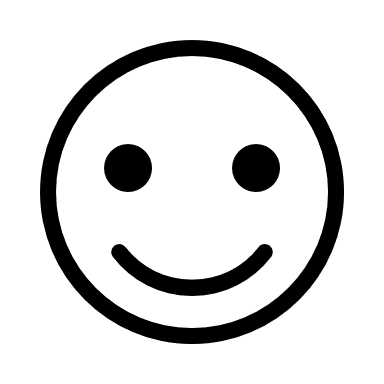 | 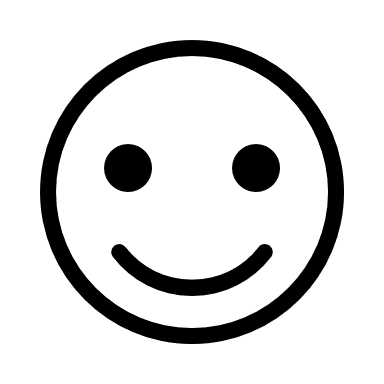 | ? | 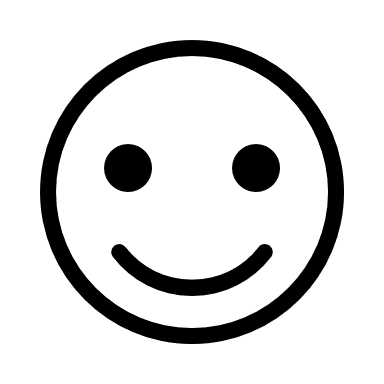 | 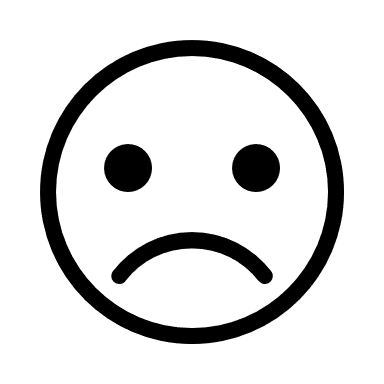 | 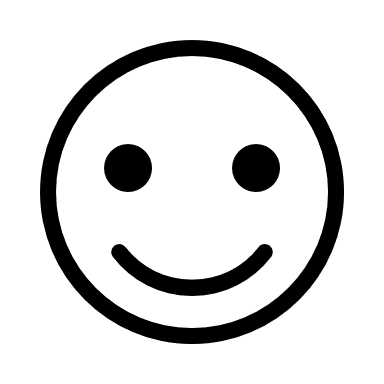 | 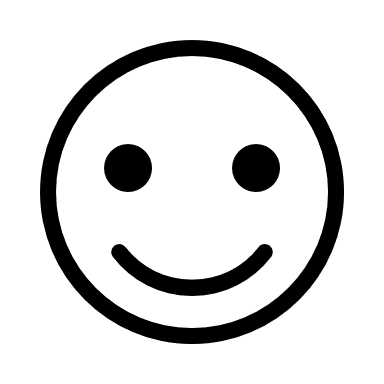 |
| Maksimowicz, 2009 | 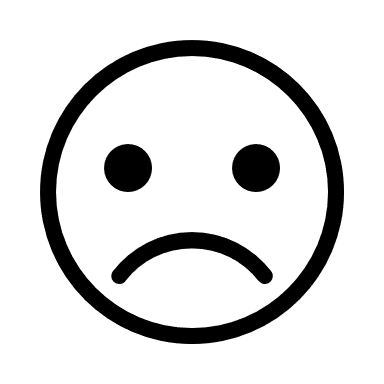 | 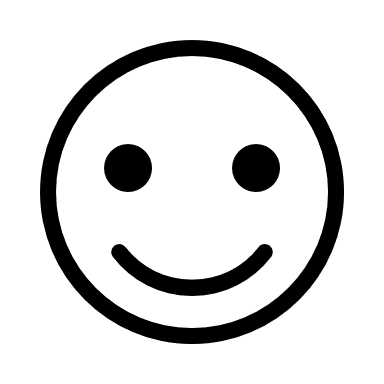 | 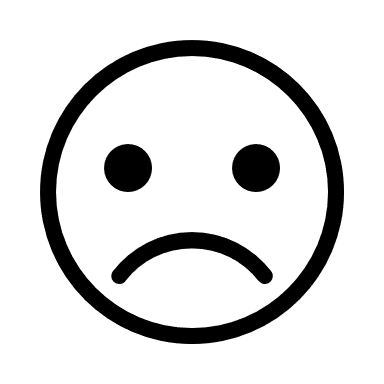 | 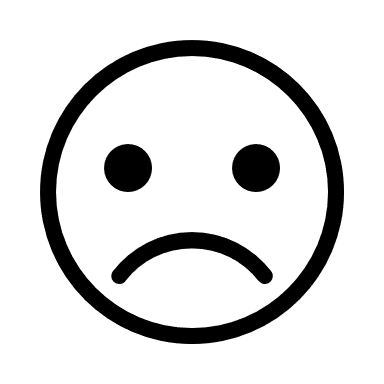 | 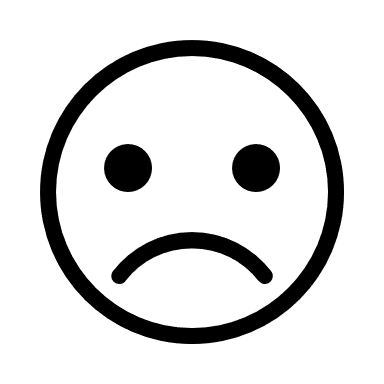 | 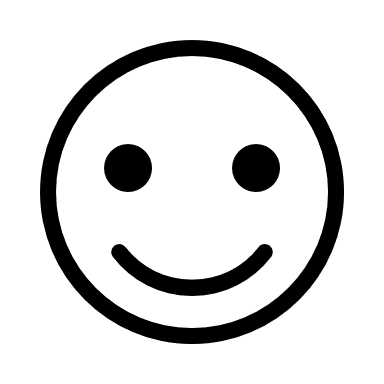 | ? |
| Ruperto, 2010 | 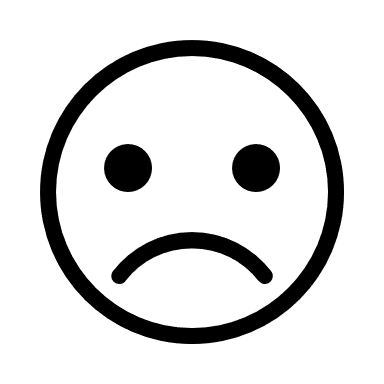 | 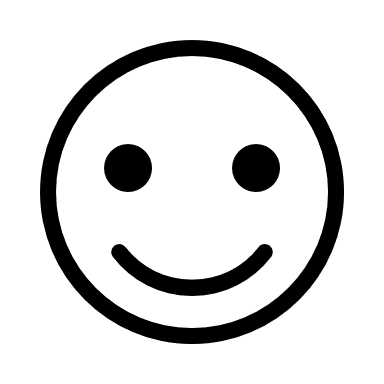 | 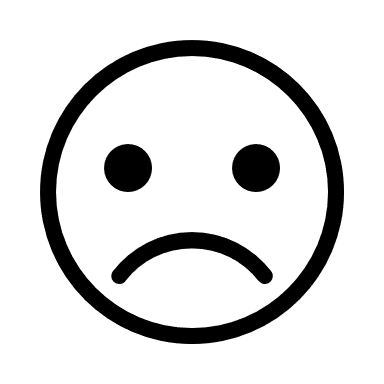 | 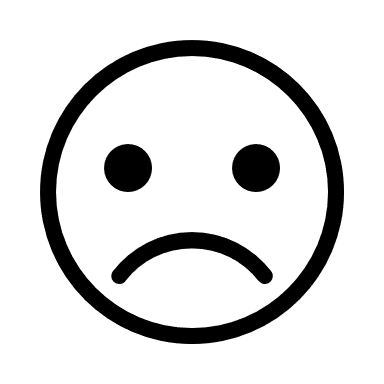 | 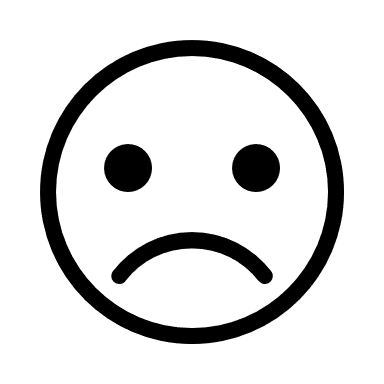 | 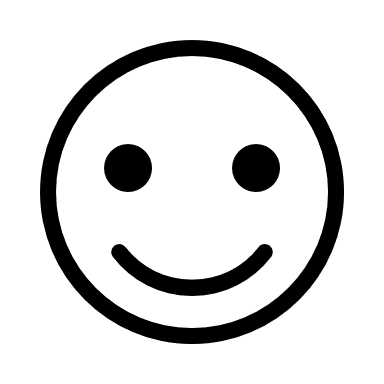 | ? |
| Furuta, 2015 | 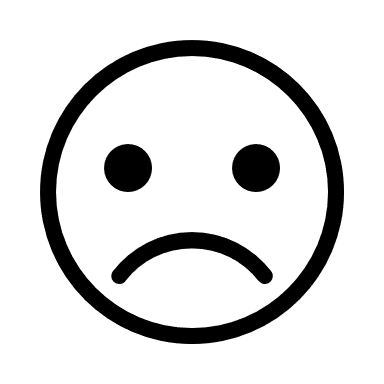 | 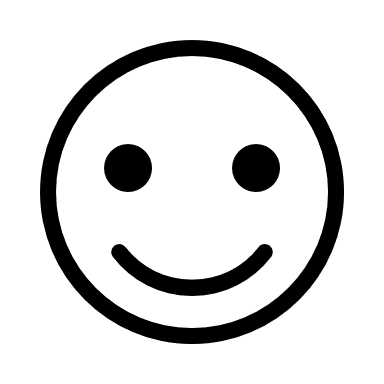 | 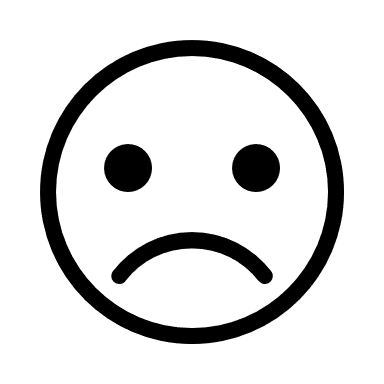 | 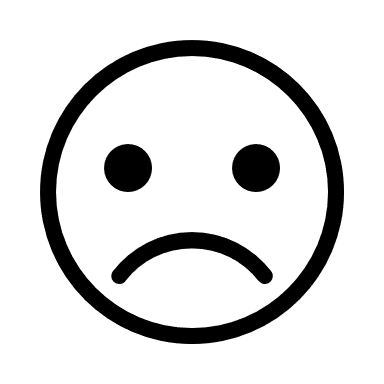 | 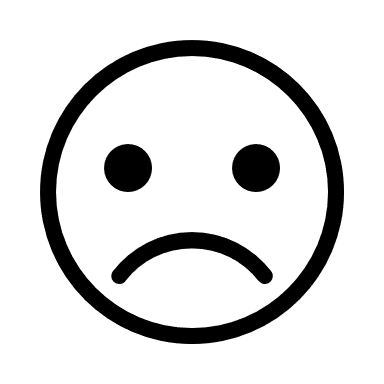 | 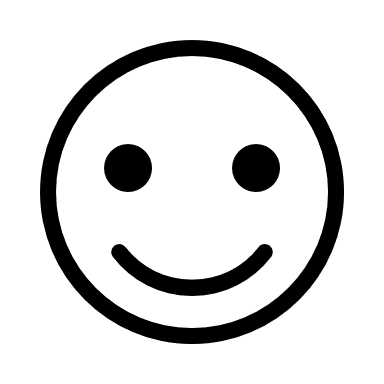 | ? |
| Kermani, 2015 | 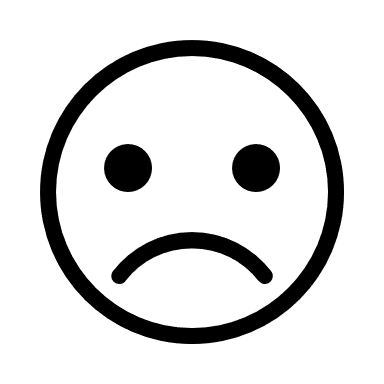 | 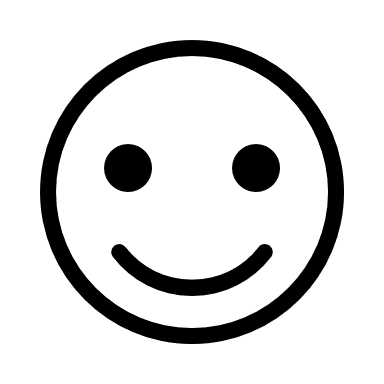 | 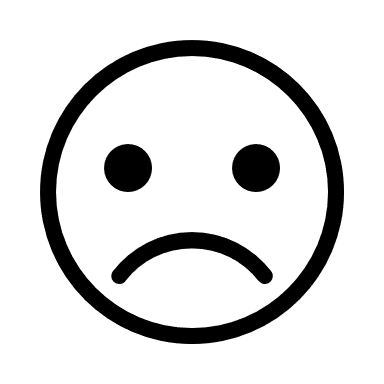 | 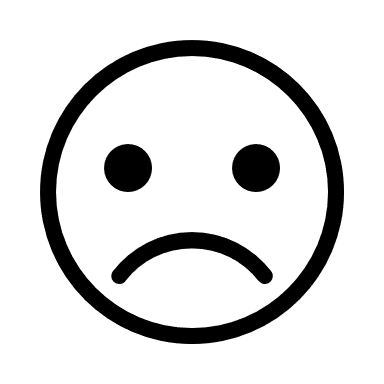 | 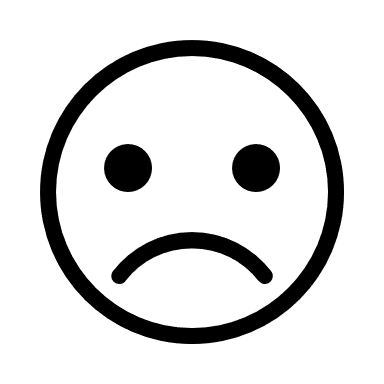 | 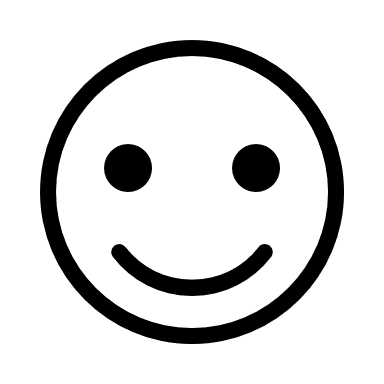 | ? |
| Kong, 2015 | 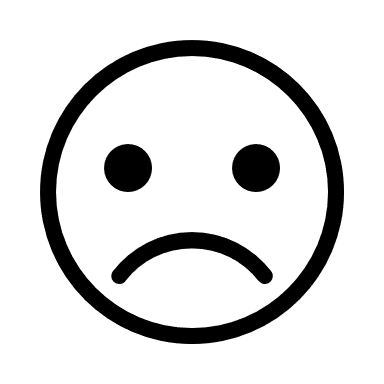 | 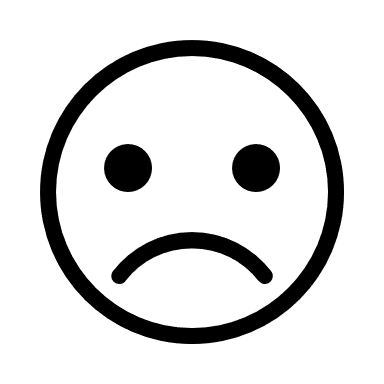 | 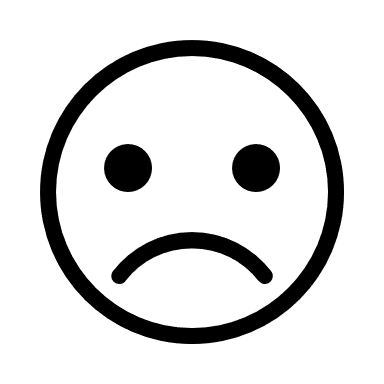 | 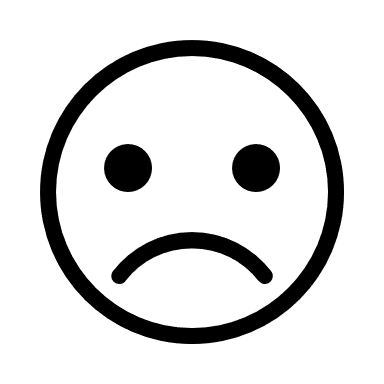 | 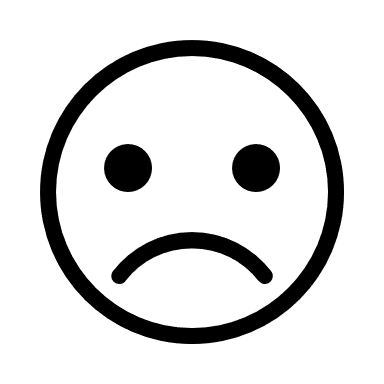 | 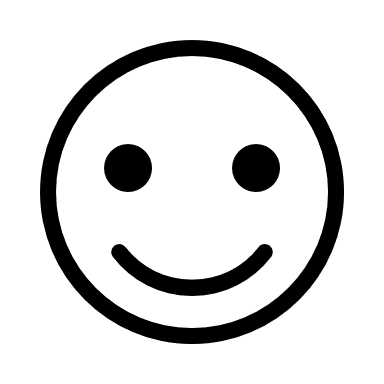 | 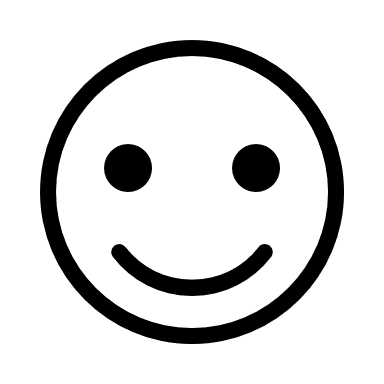 |
| Fukui, 2019 | 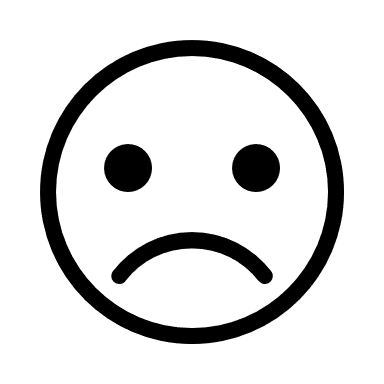 | 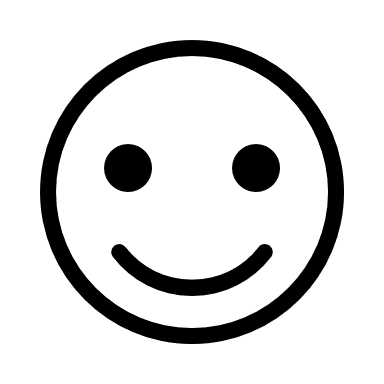 | 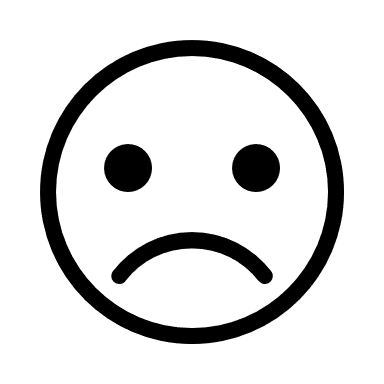 | 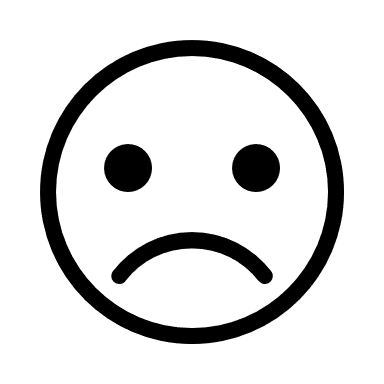 | 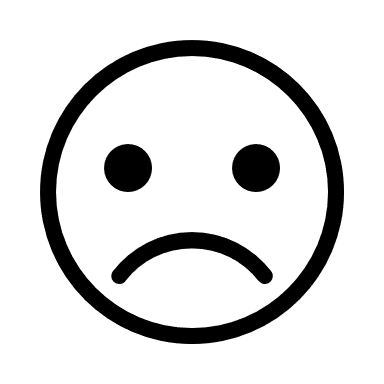 | 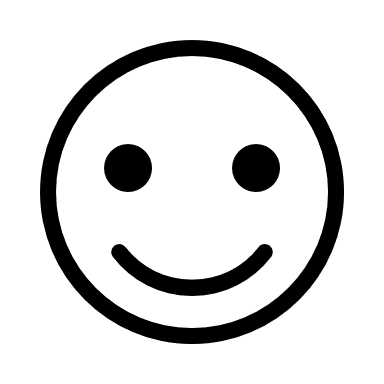 | 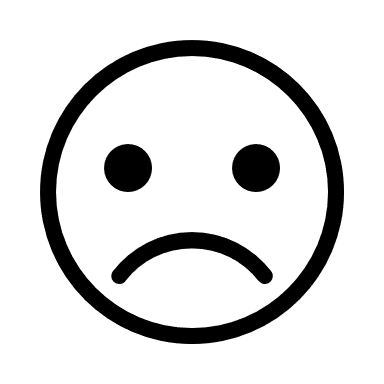 |
| Choi, 2020 | 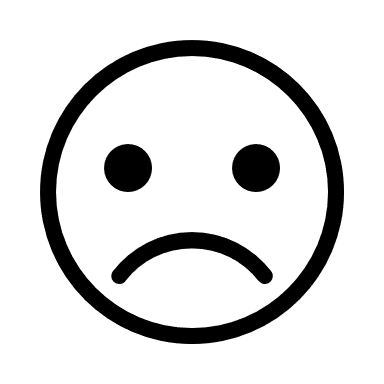 | 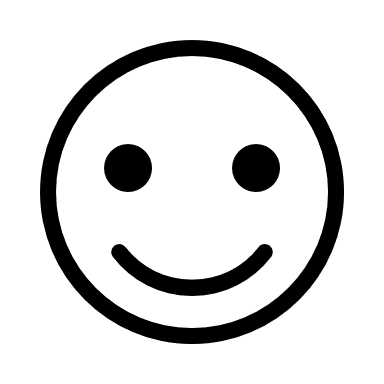 | 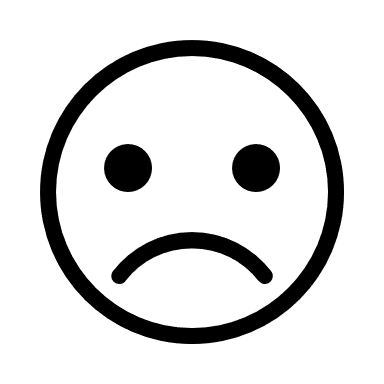 | 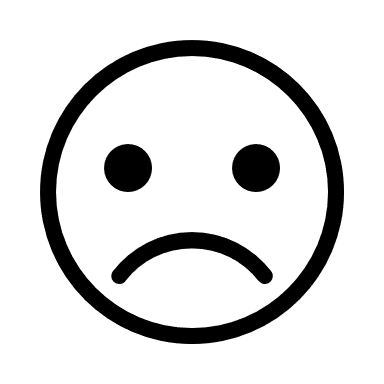 | 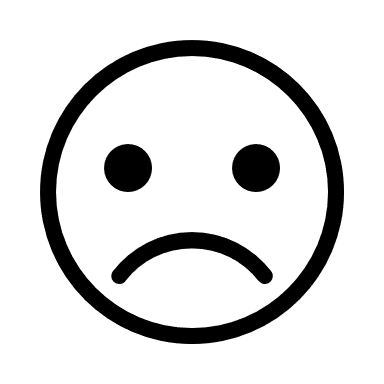 | 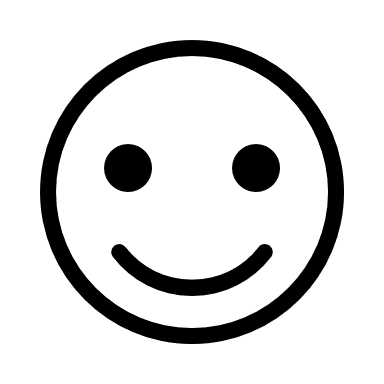 | 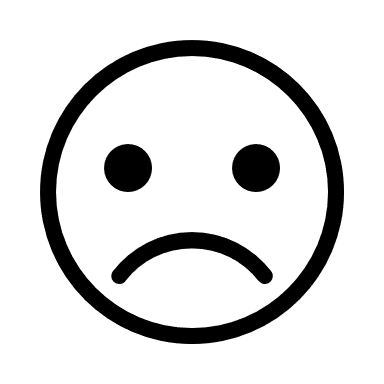 |
| Vautier, 2020 | 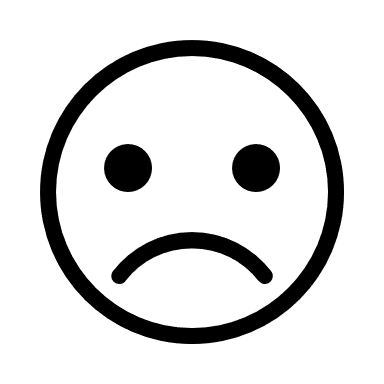 | 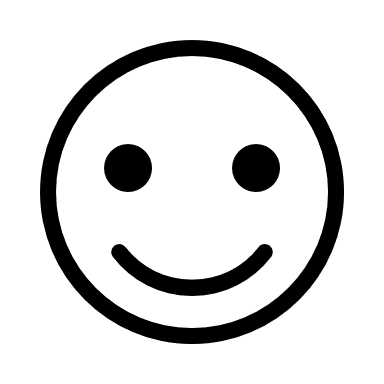 | 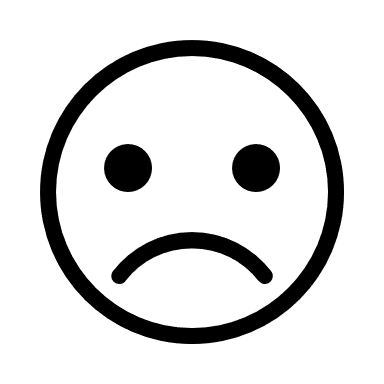 | 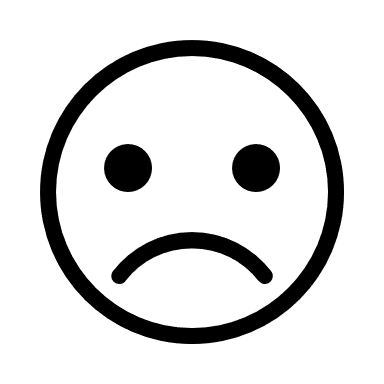 | 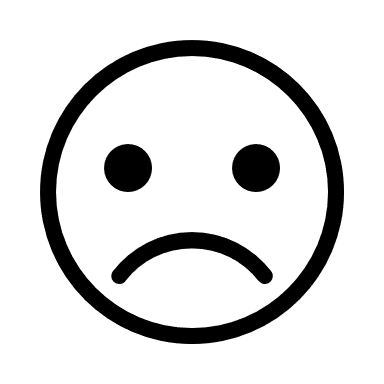 | 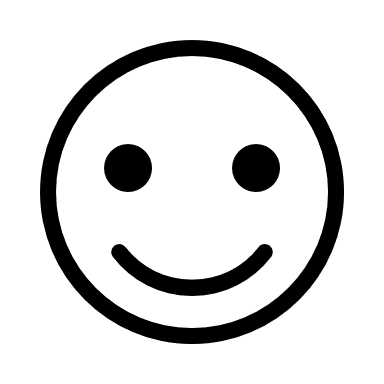 | 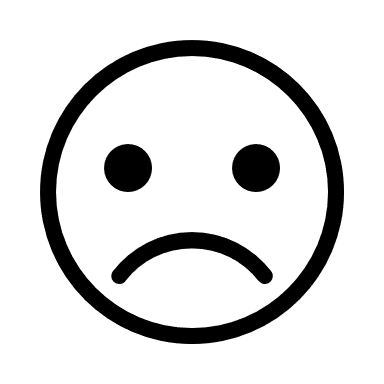 |
| Grayson, 2022 | 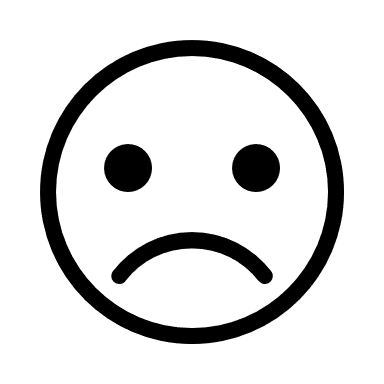 | 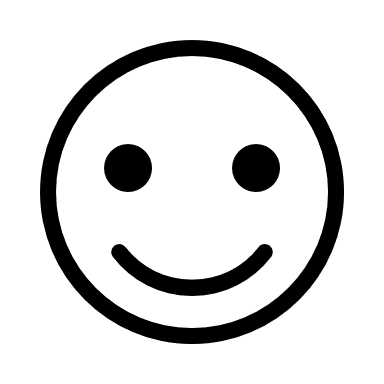 | 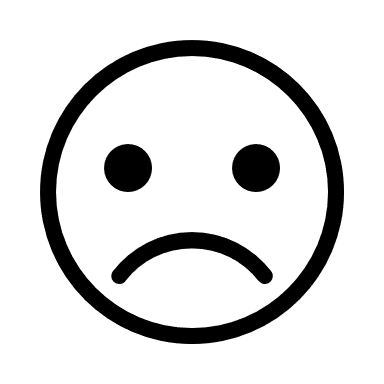 | ? | 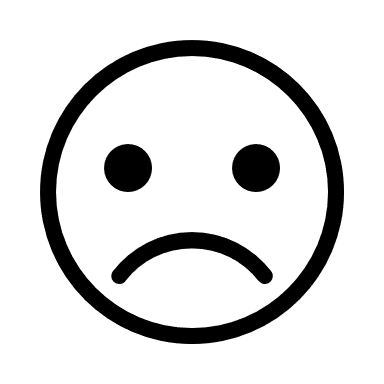 | 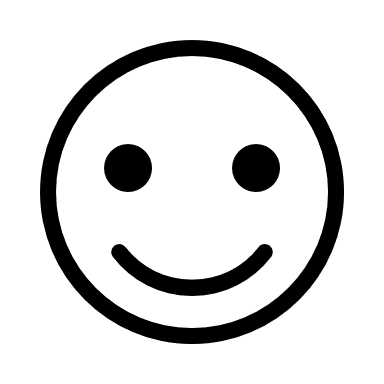 | 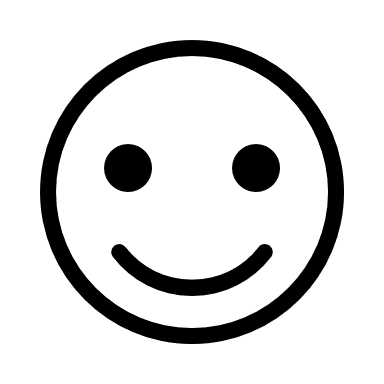 |
| Boiardi, 2023 | 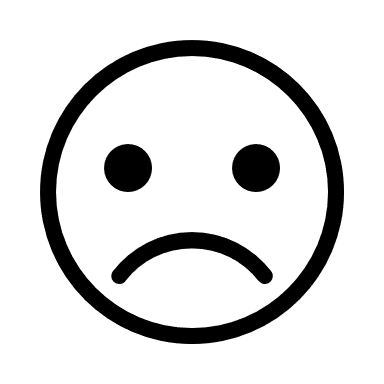 | 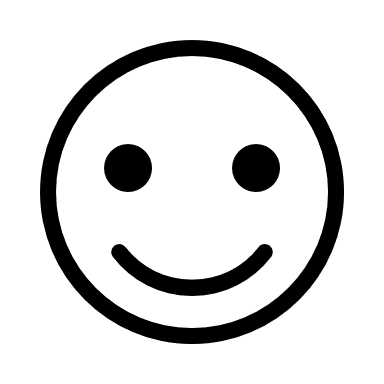 | 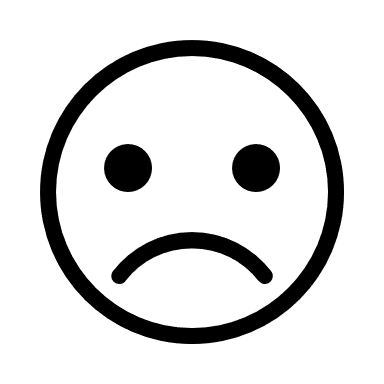 | 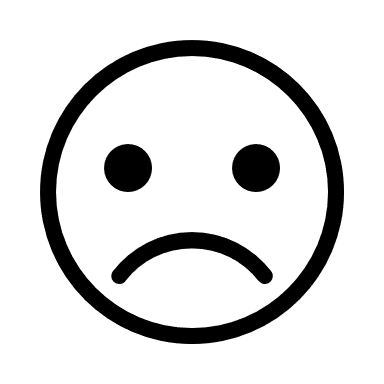 | 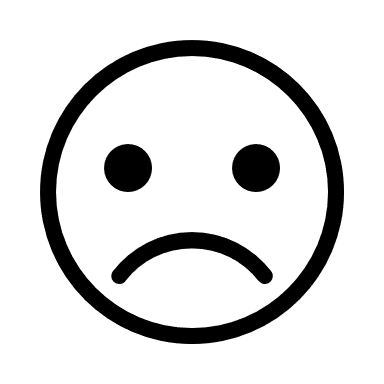 | 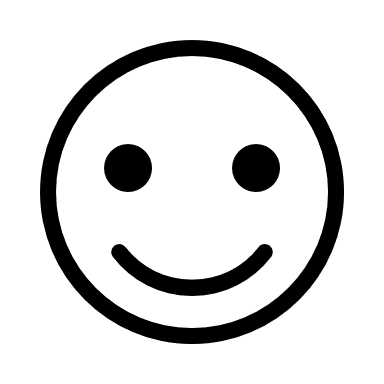 | 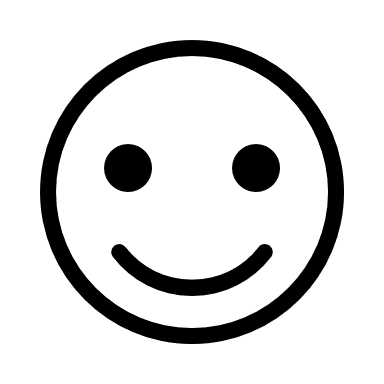 |
| Cao, 2023 | 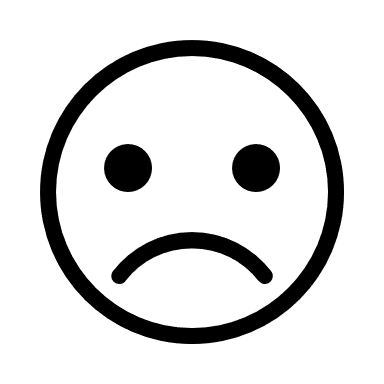 | 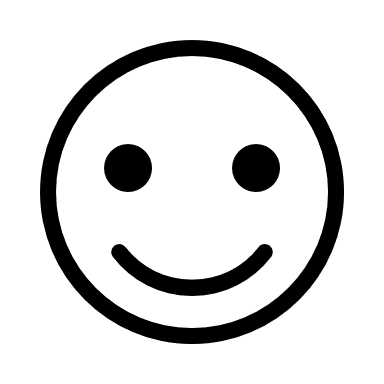 | 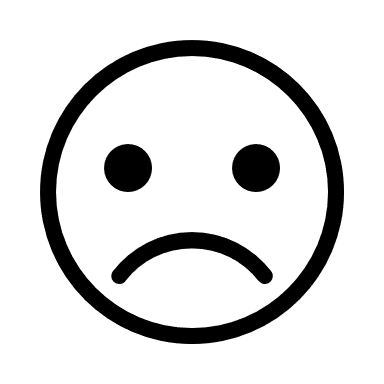 | ? | 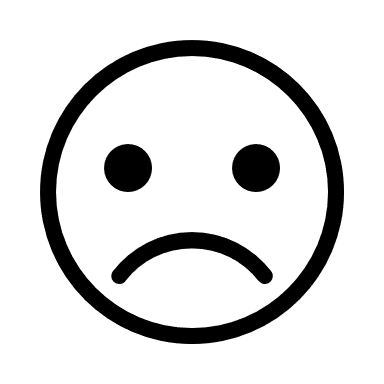 | 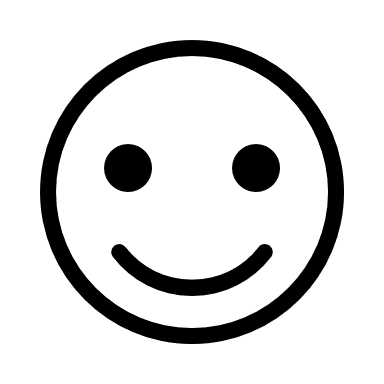 | 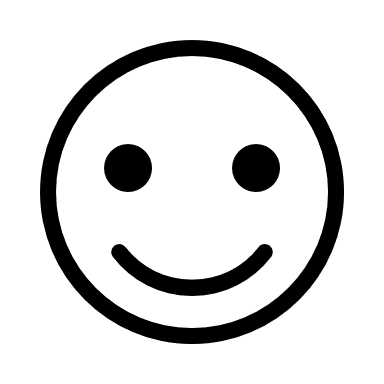 |
| Meng, 2023 | 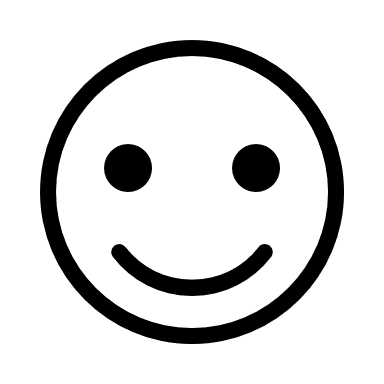 | 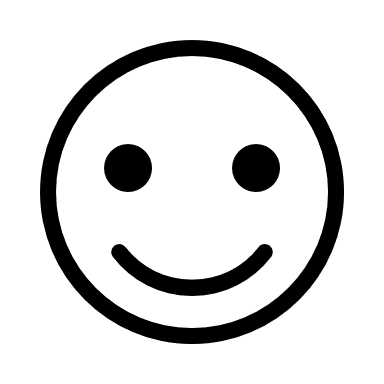 | 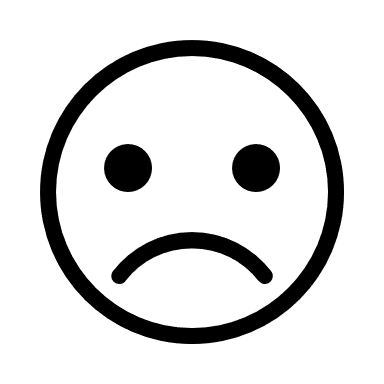 | ? | 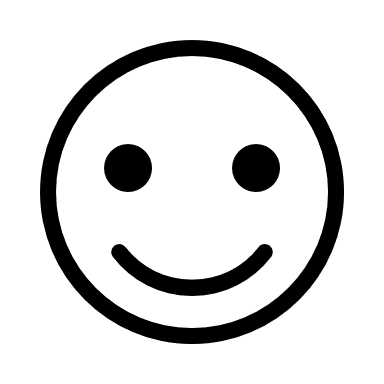 | 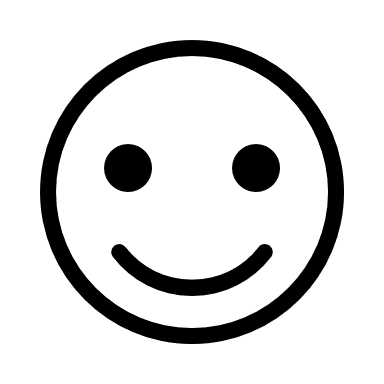 | 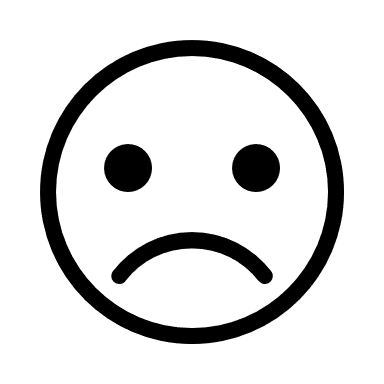 |

| 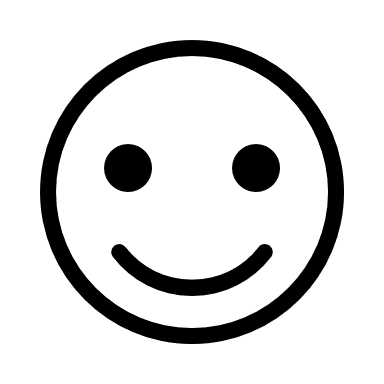 | 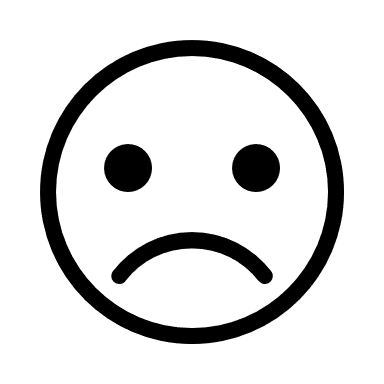 | ? |
| --- | --- | --- |
| Low | High | Unclear |

**Supplementary table S.4.**

| Symptoms and physical signs | Number of studies | Number of TA cases | Number of comparators | Se  (95 % CI) | Sp (95 % CI) | PLR (95 % CI) | NLR (95 % CI) |
| --- | --- | --- | --- | --- | --- | --- | --- |
| Age at onset < 40  (27,31) | 2 | 161 | 838 | 84.5  (48.0 - 97.0) | 78.0  (61.5 - 88.7) | 3.50  (2.26 - 5.43) | 0.19  (0.03 - 1.10) |
| Carotid bruit*  (36,38) | 2 | 195 | 165 | 43.8  (34.4 - 53.8) | 73.9  (66.7 - 80.1) | 1.68  (1.25 - 2.27) | 0.76  (0.65 - 0.89) |
| Diplopia* [33,41] | 2 | 126 | 196 | 0.0 (NA-NA) | 96.1 (87.7 – 98.9) | 0.18  (0.02 – 1.41) | 1.04 (0.97 – 1.13) |
| Dizziness [37,39] | 2 | 305 | 149 | 18.2  (12.9 – 25.0) | 90.6  (84.8 – 94.4) | 1.71 (0.96 – 3.03) | 0.93 (0.86 – 1.00) |
| Femoral bruit*  (36,38) | 2 | 194 | 149 | 14.4  (10.2 - 20.1) | 84.6  (77.8 - 89.5) | 0.95  (0.56 - 1.58) | 1.01  (0.92 - 1.10) |
| Palpitations [30,39] | 2 | 331 | 124 | 15.2 (9.12 – 24.3) | 82.8 (69.8 – 90.9) | 0.93 (0.59 – 1.46) | 1.0 (0.92 – 1.1) |
| *Polymyalgia Rheumatica** [35,41] | 2 | 74 | 149 | 0.0 (NA-NA) | 73.2 (65.5 – 79.7) | 0.07 (0.01 – 0.48) | 1.29  (1.07 – 1.56) |
| Scalp tenderness* [33,41] | 2 | 126 | 171 | 0.0 (NA-NA) | 81.3 (74.7 – 86.5) | 0.04 (0.01 – 0.3) | 1.22 (1.12 – 1.34) |
| Sensory peripheral neuropathy [34,39] | 2 | 293 | 1087 | 1.6 (0.0 – 80.1) | 92.9 (77.6 – 98.0) | 0.77 (0.17 – 3.59) | 1.03 (1.01 – 1.05) |
| Syncope [31,39] | 2 | 316 | 78 | 21.4 (8.4 – 44.6) | 86.1 (63.8 – 95.8) | 1.42 (0.81 – 2.47) | 0.94  (0.87 – 1.02) |
| Weakness [37,42] | 2 | 225 | 258 | 41.5 (32.1 – 51.6) | 91.1 (86.9 – 94.0) | 4.57  (3.02 – 6.94) | 0.65 (0.54 – 0.77) |

**Supplementary table S.5.**

| Symptoms and physical signs | Number of studies | Number of TA cases | Number of GCA cases | Se (95 % CI) | Sp (95 % CI) | PLR (95 % CI) | NLR (95 % CI) |
| --- | --- | --- | --- | --- | --- | --- | --- |
| Headache [33,35,36,38,40,41] | 6 | 591 | 496 | 24.7  (11.5 – 45.2) | 48.7  (30.3 – 67.4) | 0.51 (0.25 – 0.86) | 1.57  (1.1 – 2.32) |
| Fever [33,36,38,40,41] | 5 | 560 | 454 | 27.5 (15.7 – 43.7) | 69.5 (50.8 – 83.4) | 0.94  (0.53 – 1.56) | 1.06 (0.83 – 1.35) |
| Arm claudication [33,36,40] | 3 | 498 | 307 | 34.9  (29.3 – 41.0) | 78.0  (44.5 – 94.0) | 1.93 (0.75 – 5.2) | 0.89  (0.72 – 1.38) |
| Blindness [33,35,41] | 3 | 149 | 218 | 2.0 (0.4 – 10.8) | 94.1 (81.1 – 98.3) | 0.79 (0.03 – 4.3) | 1.05 (0.92 – 1.22) |
| Blood pressure asymmetry [33,36,40] | 3 | 495 | 300 | 54.3 (41.2 – 66.8) | 83.8 (57.3 – 95.2) | 3.83 (1.52 – 9.0) | 0.56 (0.48 – 0.65) |
| Decreased or absent pulse [36,40,41] | 3 | 462 | 327 | 60.5 (44.7 – 74.4) | 79.5 (43.9 – 95.1) | 3.55  (1.3 – 9.35) | 0.52  (0.43 – 0.64) |
| Hypertension [33,36,41] | 3 | 209 | 312 | 32.3  (25.5 – 40.0) | 60.3  (36.0 – 80.3) | 0.87 (0.53 – 1.52) | 1.18  (0.86 – 1.79) |
| Jaw claudication [33,35,41] | 3 | 149 | 218 | 2.7  (0.8 – 9.2) | 79.8 (62.5 – 90.3) | 0.15 (0.05 – 0.35) | 1.24 (1.09 – 1.5) |
| Leg claudication [33,36,40] | 3 | 498 | 307 | 17.2 (12.1 – 23.8) | 87.8 (79.4 – 93.1) | 1.44  (0.94 – 2.15) | 0.95 (0.89 – 1.01) |
| Myocardial infarction [35,40,41] | 3 | 381 | 267 | 6.7 (2.3 – 18.2) | 98.6 (94.5 – 99.7) | 5.86 (1.5 – 16.0) | 0.94 (0.84 – 0.99) |
| Stroke or   transient ischemic attack  [33,36,41] | 3 | 231 | 316 | 9.4 (6.0 – 14.2) | 95.5  (92.6 – 97.3) | 2.2 (1.08 - 4.09) | 0.95 (0.9 – 1.0) |
| Abdominal bruit [33,36] | 2 | 196 | 145 | 24.6 (12.3 – 43.1) | 88.3 (81.4 – 92.9) | 2.05 (0.42 – 9.94) | 0.84  (0.58 – 1.22) |
| Amaurosis [33,41] | 2 | 134 | 196 | 3.0  (1.12 – 7.68) | 93.9  (85.3 – 97.6) | 0.39 (0.13 – 1.17) | 1.04 (0.97 – 1.10) |
| Arthralgia [33,40] | 2 | 374 | 187 | 21.9 (18.0 – 26.4) | 64.7 (57.6 – 71.2) | 0.63  (0.48 – 0.83) | 1.2 (1.06 – 1.35) |
| Carotid bruit  (36,38) | 2 | 195 | 165 | 43.8  (34.4 - 53.8) | 73.9  (66.7 - 80.1) | 1.68  (1.25 - 2.27) | 0.76  (0.65 - 0.89) |
| Diplopia [33,41] | 2 | 126 | 196 | 0.0 (NA-NA) | 96.1 (87.7 – 98.9) | 0.18  (0.02 – 1.41) | 1.04 (0.97 – 1.13) |
| Femoral bruit  (36,38) | 2 | 194 | 149 | 14.4  (10.2 - 20.1) | 84.6  (77.8 - 89.5) | 0.95  (0.56 - 1.58) | 1.01  (0.92 - 1.10) |
| Myalgia [33,40] | 2 | 374 | 187 | 7.25 (2.32 – 20.4) | 65.3 (49.6 – 78.3) | 0.22 (0.08 – 0.26) | 1.38 (1.16 – 1.64) |
| *Polymyalgia Rheumatica* [35,41] | 2 | 74 | 149 | 0.0 (NA-NA) | 73.2 (65.5 – 79.7) | 0.07 (0.01 – 0.48) | 1.29  (1.07 – 1.56) |
| Scalp tenderness [33,41] | 2 | 126 | 171 | 0.0 (NA-NA) | 81.3 (74.7 – 86.5) | 0.04 (0.01 – 0.3) | 1.22 (1.12 – 1.34) |
| Stroke [33,40] | 2 | 374 | 187 | 9.63 (7.02 – 13.1) | 96.3 (92.4 – 98.2) | 2.29 (0.95 – 5.51) | 0.95 (0.89 – 1.02) |
| Subclavian arteries bruit [33,36] | 2 | 195 | 170 | 28.7 (9.69 – 60.0) | 79.8 (62.7 – 90.3) | 1.55 (1.13 – 2.14) | 0.85  (0.63 – 1.15) |
| Vascular bruits [40,41] | 2 | 340 | 203 | 56.1 (42.5 – 68.9) | 73.7 (64.8 – 81.0) | 2.2 (1.71 – 2.87) | 0.60 (0.48-0.75) |
| Weight loss [36,40] | 2 | 402 | 238 | 24.9 (14.2 – 39.9) | 62.6 (55.6 – 69.2) | 0.67 (0.25 – 1.79) | 1.17 (0.78 – 1.76) |

**Supplementary table S.6.**
