# Supplementary material for: Diagnostic Accuracy of Clinical Findings for Takayasu Arteritis: A Rapid Review and Meta-Analysis
Source: Int J Vasc Med. 2025 Sep 9;2025:6092362. doi: 10.1155/ijvm/6092362 (PMC12440657; doi:10.1155/ijvm/6092362)
Supplement: Supporting Information 2 — Figure S1: Paired forest plots of sensitivity and specificity for each symptom, physical sign, demographic feature, and complications listed in alphabetical order. [file 6092362.f2.pdf]

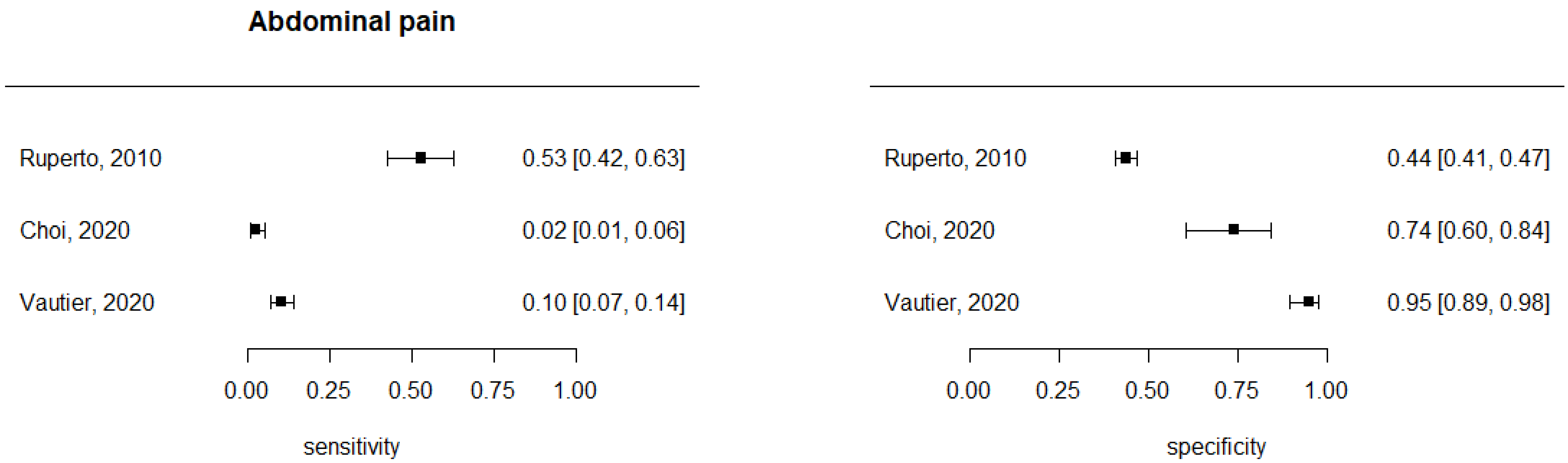
Abdominal pain
Ruperto, 2010
0.53 [0.42, 0.63]
Choi, 2020
0.02 [0.01, 0.06]
Vautier, 2020
0.10 [0.07, 0.14]
0.00
0.25
0.50
0.75
1.00
sensitivity
Ruperto, 2010
0.44 [0.41, 0.47]
Choi, 2020
0.74 [0.60, 0.84]
Vautier, 2020
0.95 [0.89, 0.98]
0.00
0.25
0.50
0.75
1.00
specificity

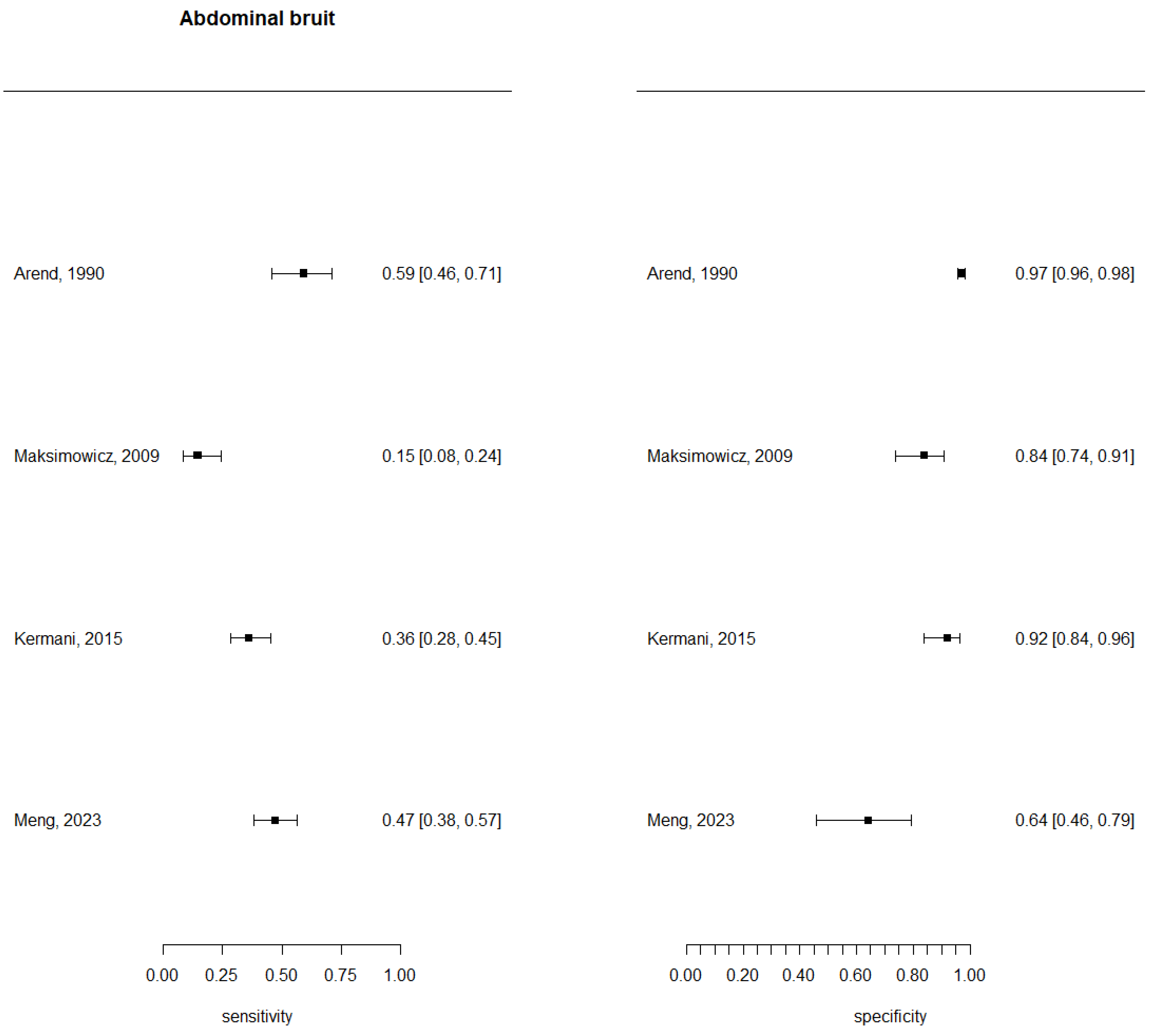
Abdominal bruit
Arend, 1990
0.59 [0.46, 0.71]
Maksimowicz, 2009
0.15 [0.08, 0.24]
Kermani, 2015
0.36 [0.28, 0.45]
Meng, 2023
0.47 [0.38, 0.57]
0.00
0.25
0.50
0.75
1.00
sensitivity
Arend, 1990
0.97 [0.96, 0.98]
Maksimowicz, 2009
0.84 [0.74, 0.91]
Kermani, 2015
0.92 [0.84, 0.96]
Meng, 2023
0.64 [0.46, 0.79]
0.00
0.20
0.40
0.60
0.80
1.00
specificity

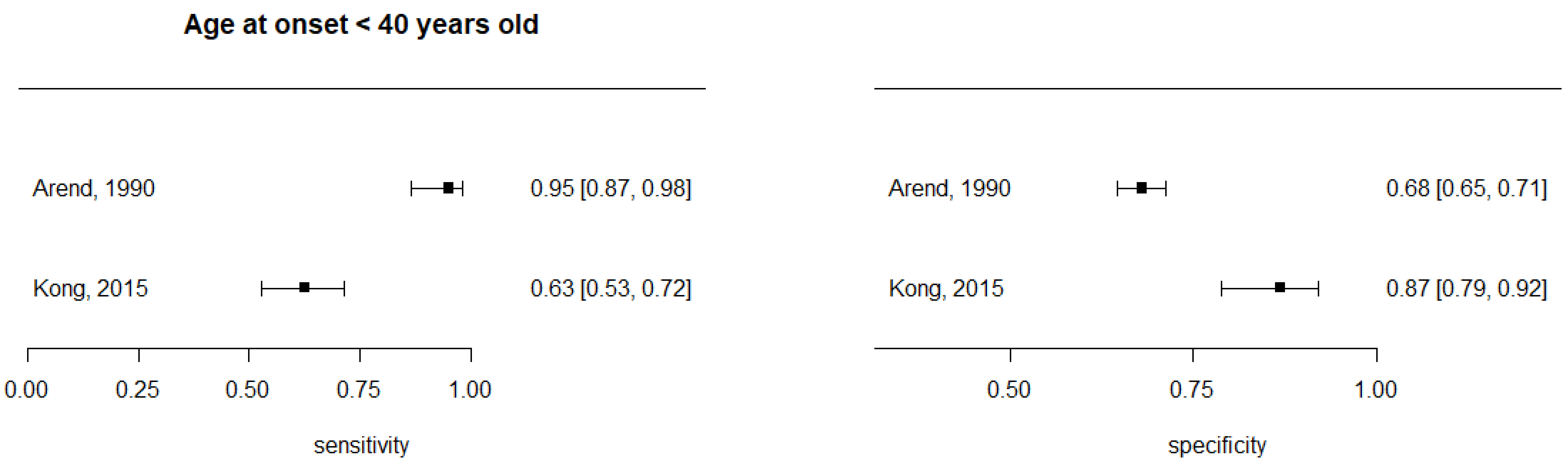

Age at onset < 40 years old
Arend, 1990
0.95 [0.87, 0.98]
Kong, 2015
0.63 [0.53, 0.72]
0.00
0.25
0.50
0.75
1.00
sensitivity
Arend, 1990
0.68 [0.65, 0.71]
Kong, 2015
0.87 [0.79, 0.92]
0.50
0.75
1.00
specificity

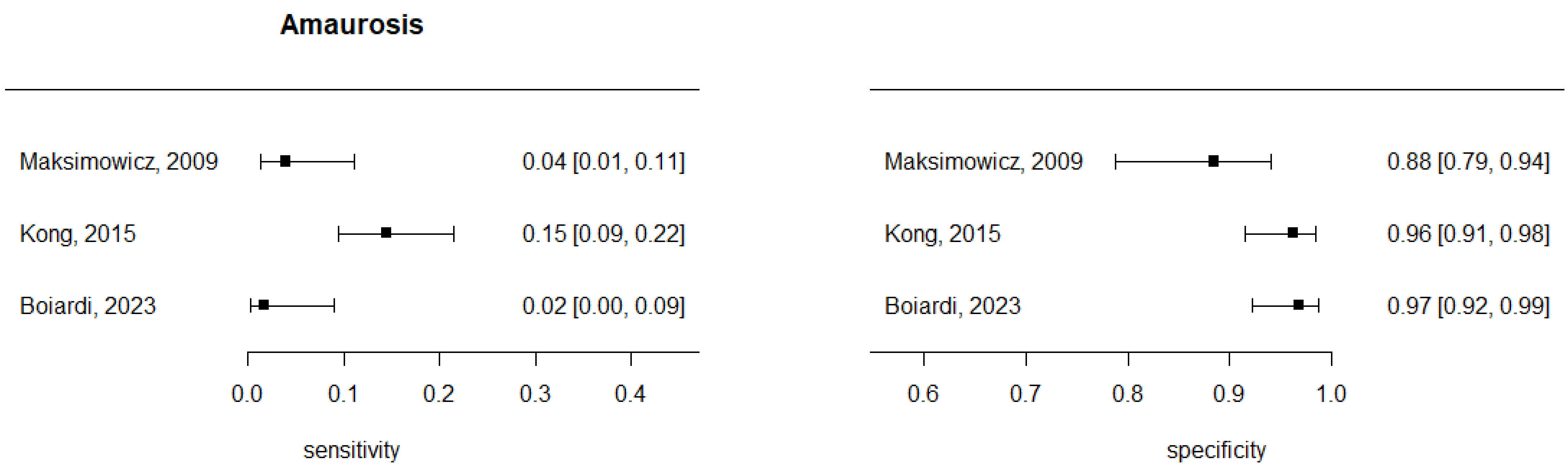
Amaurosis
Maksimowicz, 2009
Kong, 2015
Boiardi, 2023
0.04 [0.01, 0.11]
0.15 [0.09, 0.22]
0.02 [0.00, 0.09]
0.0
0.1
0.2
0.3
0.4
sensitivity
Maksimowicz, 2009
Kong, 2015
Boiardi, 2023
0.88 [0.79, 0.94]
0.96 [0.91, 0.98]
0.97 [0.92, 0.99]
0.6
0.7
0.8
0.9
1.0
specificity

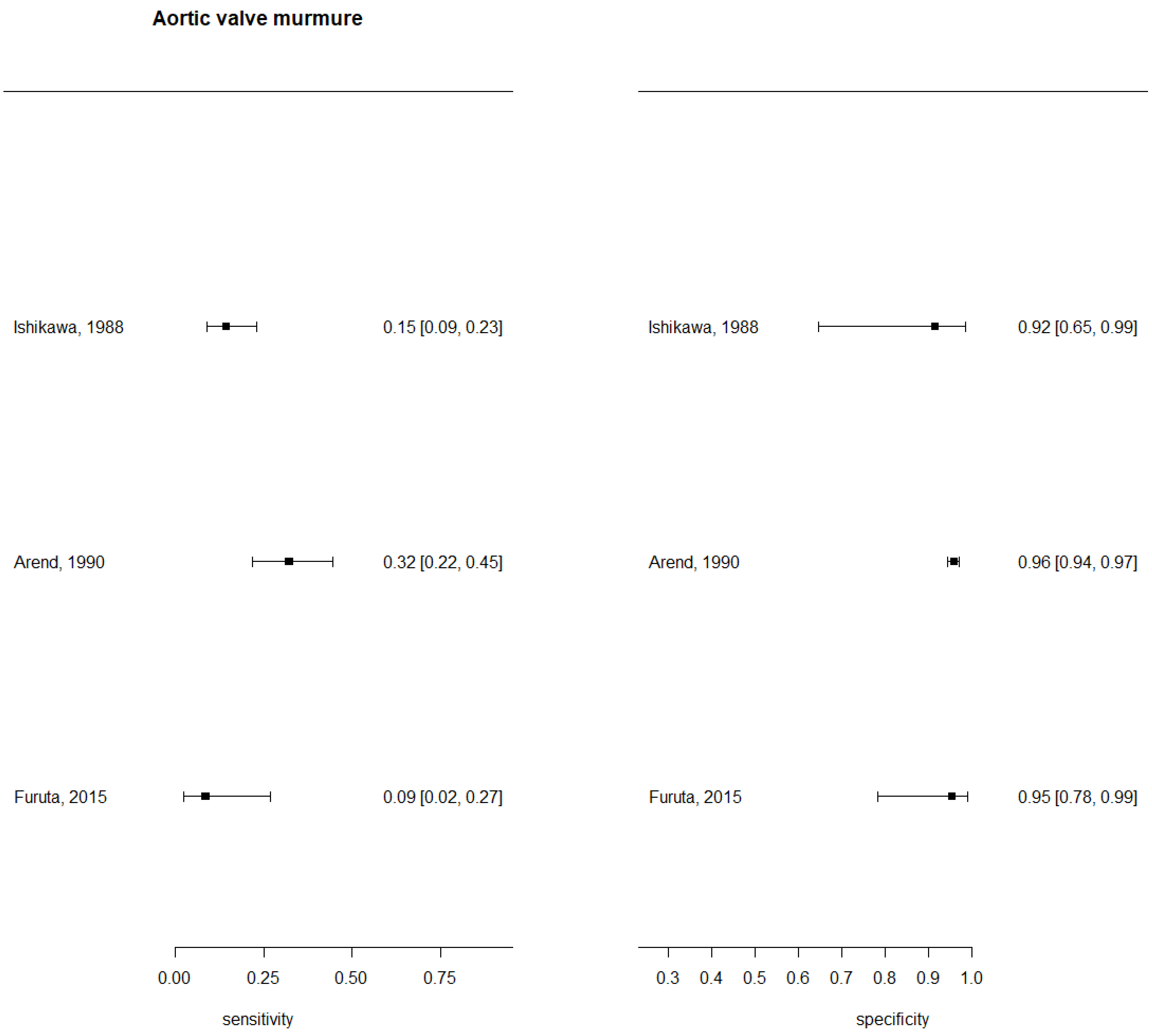
Aortic valve murmure
Ishikawa, 1988
0.15 [0.09, 0.23]
Arend, 1990
0.32 [0.22, 0.45]
Furuta, 2015
0.09 [0.02, 0.27]
0.00
0.25
0.50
0.75
sensitivity
Ishikawa, 1988
0.92 [0.65, 0.99]
Arend, 1990
0.96 [0.94, 0.97]
Furuta, 2015
0.95 [0.78, 0.99]
0.3
0.4
0.5
0.6
0.7
0.8
0.9
1.0
specificity

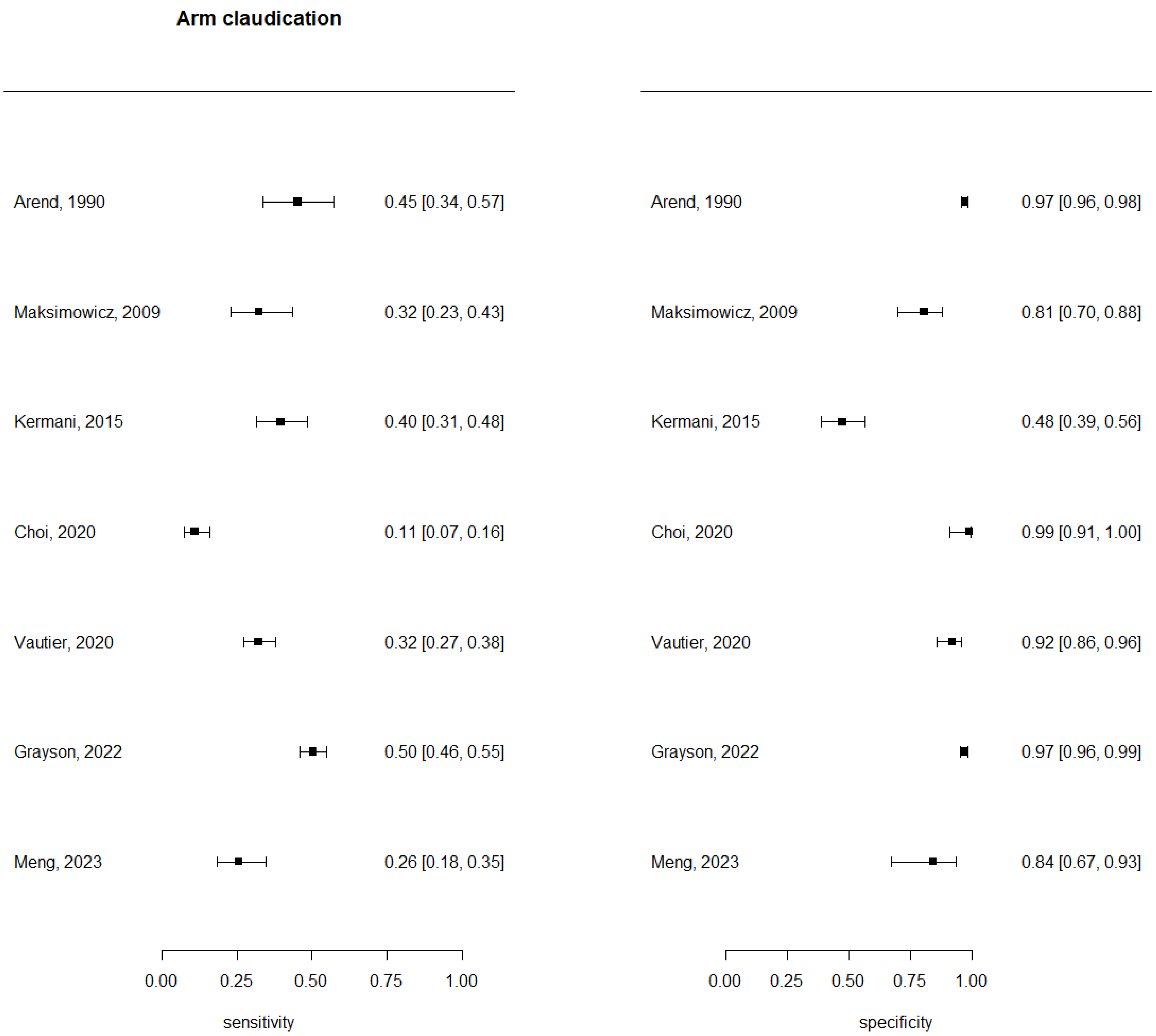

Arm claudication
Arend, 1990
0.45 [0.34, 0.57]
Maksimowicz, 2009
0.32 [0.23, 0.43]
Kermani, 2015
0.40 [0.31, 0.48]
Choi, 2020
0.11 [0.07, 0.16]
Vautier, 2020
0.32 [0.27, 0.38]
Grayson, 2022
0.50 [0.46, 0.55]
Meng, 2023
0.26 [0.18, 0.35]
0.00 0.25 0.50 0.75 1.00
sensitivity
Arend, 1990
0.97 [0.96, 0.98]
Maksimowicz, 2009
0.81 [0.70, 0.88]
Kermani, 2015
0.48 [0.39, 0.56]
Choi, 2020
0.99 [0.91, 1.00]
Vautier, 2020
0.92 [0.86, 0.96]
Grayson, 2022
0.97 [0.96, 0.99]
Meng, 2023
0.84 [0.67, 0.93]
0.00 0.25 0.50 0.75 1.00
specificity

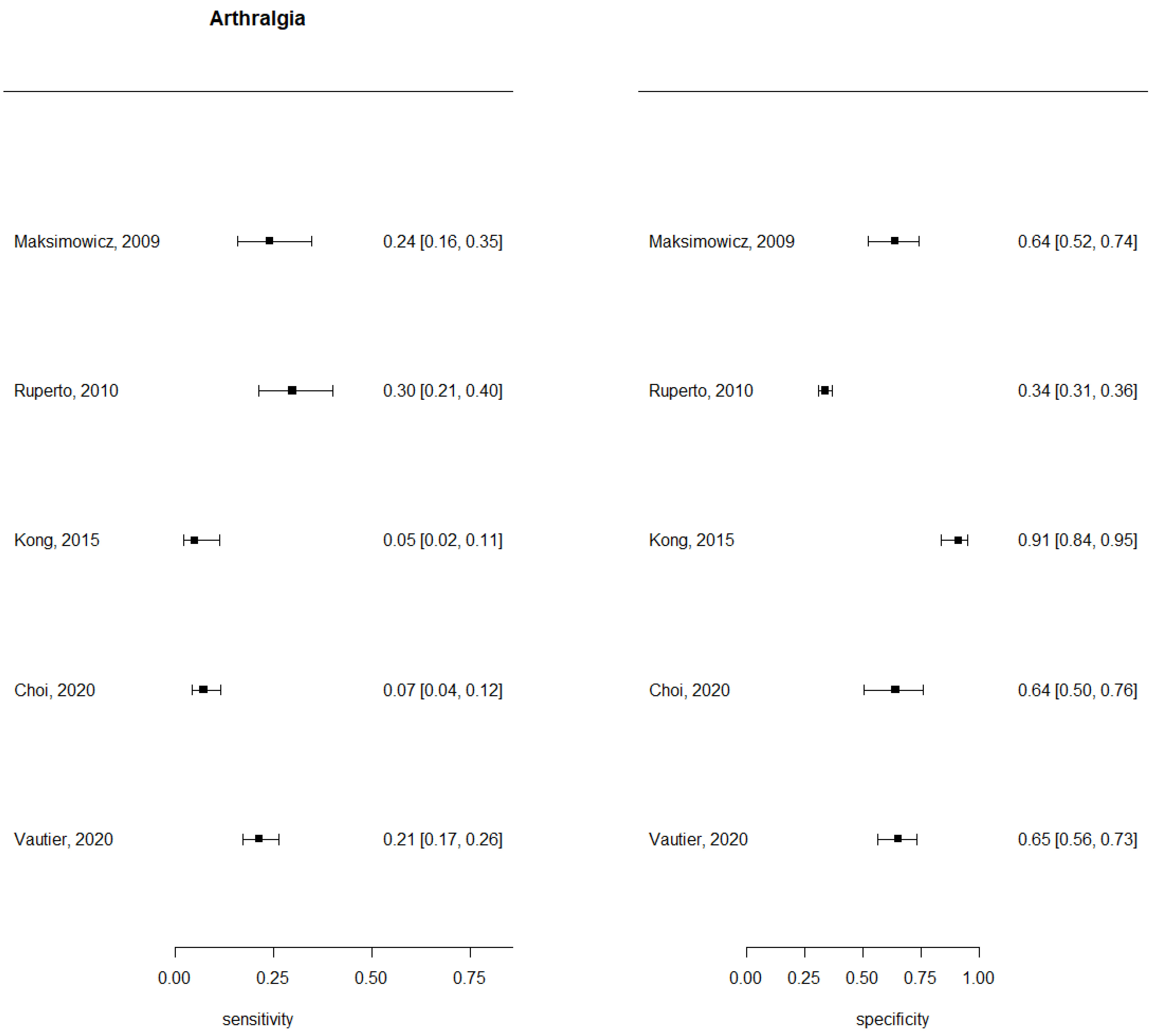

Arthralgia
Maksimowicz, 2009
0.24 [0.16, 0.35]
Ruperto, 2010
0.30 [0.21, 0.40]
Kong, 2015
0.05 [0.02, 0.11]
Choi, 2020
0.07 [0.04, 0.12]
Vautier, 2020
0.21 [0.17, 0.26]
0.00
0.25
0.50
0.75
sensitivity
Maksimowicz, 2009
0.64 [0.52, 0.74]
Ruperto, 2010
0.34 [0.31, 0.36]
Kong, 2015
0.91 [0.84, 0.95]
Choi, 2020
0.64 [0.50, 0.76]
Vautier, 2020
0.65 [0.56, 0.73]
0.00
0.25
0.50
0.75
1.00
specificity

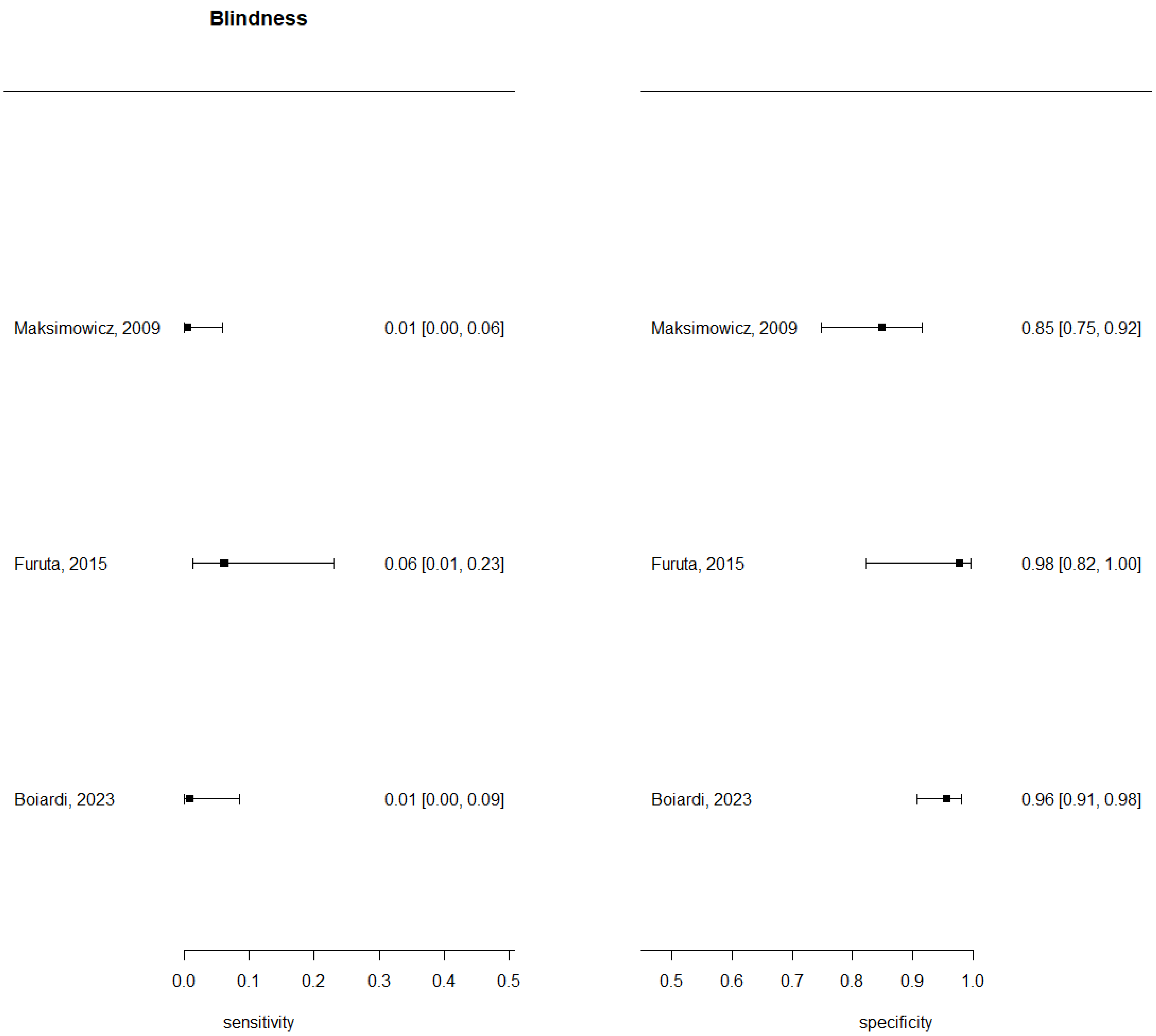
Blindness
Maksimowicz, 2009
0.01 [0.00, 0.06]
Furuta, 2015
0.06 [0.01, 0.23]
Boiardi, 2023
0.01 [0.00, 0.09]
0.0
0.1
0.2
0.3
0.4
0.5
sensitivity
Maksimowicz, 2009
0.85 [0.75, 0.92]
Furuta, 2015
0.98 [0.82, 1.00]
Boiardi, 2023
0.96 [0.91, 0.98]
0.5
0.6
0.7
0.8
0.9
1.0
specificity

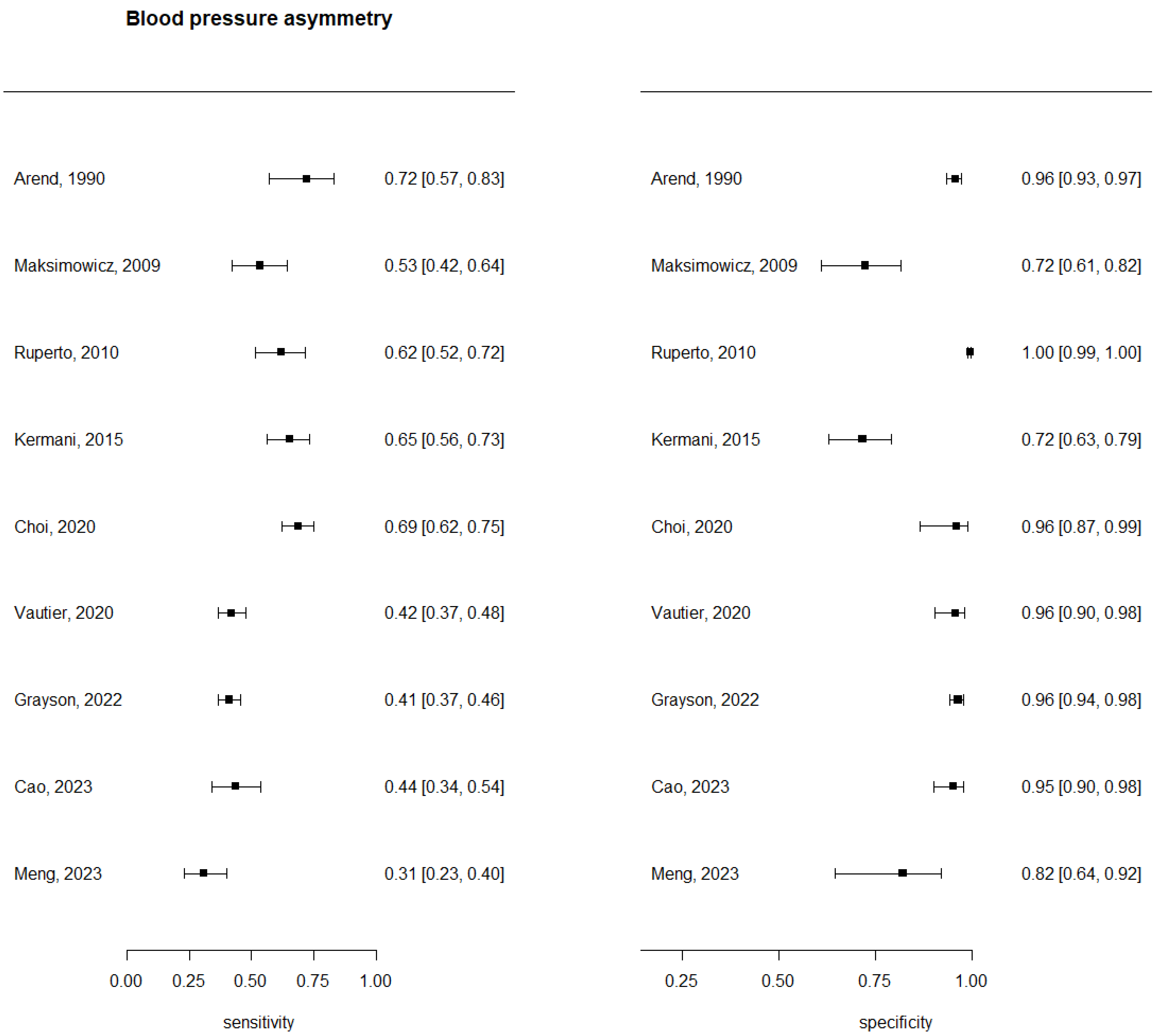
Blood pressure asymmetry
Arend, 1990
0.72 [0.57, 0.83]
Maksimowicz, 2009
0.53 [0.42, 0.64]
Ruperto, 2010
0.62 [0.52, 0.72]
Kermani, 2015
0.65 [0.56, 0.73]
Choi, 2020
0.69 [0.62, 0.75]
Vautier, 2020
0.42 [0.37, 0.48]
Grayson, 2022
0.41 [0.37, 0.46]
Cao, 2023
0.44 [0.34, 0.54]
Meng, 2023
0.31 [0.23, 0.40]
0.00
0.25
0.50
0.75
1.00
sensitivity
Arend, 1990
0.96 [0.93, 0.97]
Maksimowicz, 2009
0.72 [0.61, 0.82]
Ruperto, 2010
1.00 [0.99, 1.00]
Kermani, 2015
0.72 [0.63, 0.79]
Choi, 2020
0.96 [0.87, 0.99]
Vautier, 2020
0.96 [0.90, 0.98]
Grayson, 2022
0.96 [0.94, 0.98]
Cao, 2023
0.95 [0.90, 0.98]
Meng, 2023
0.82 [0.64, 0.92]
0.25
0.50
0.75
1.00
specificity

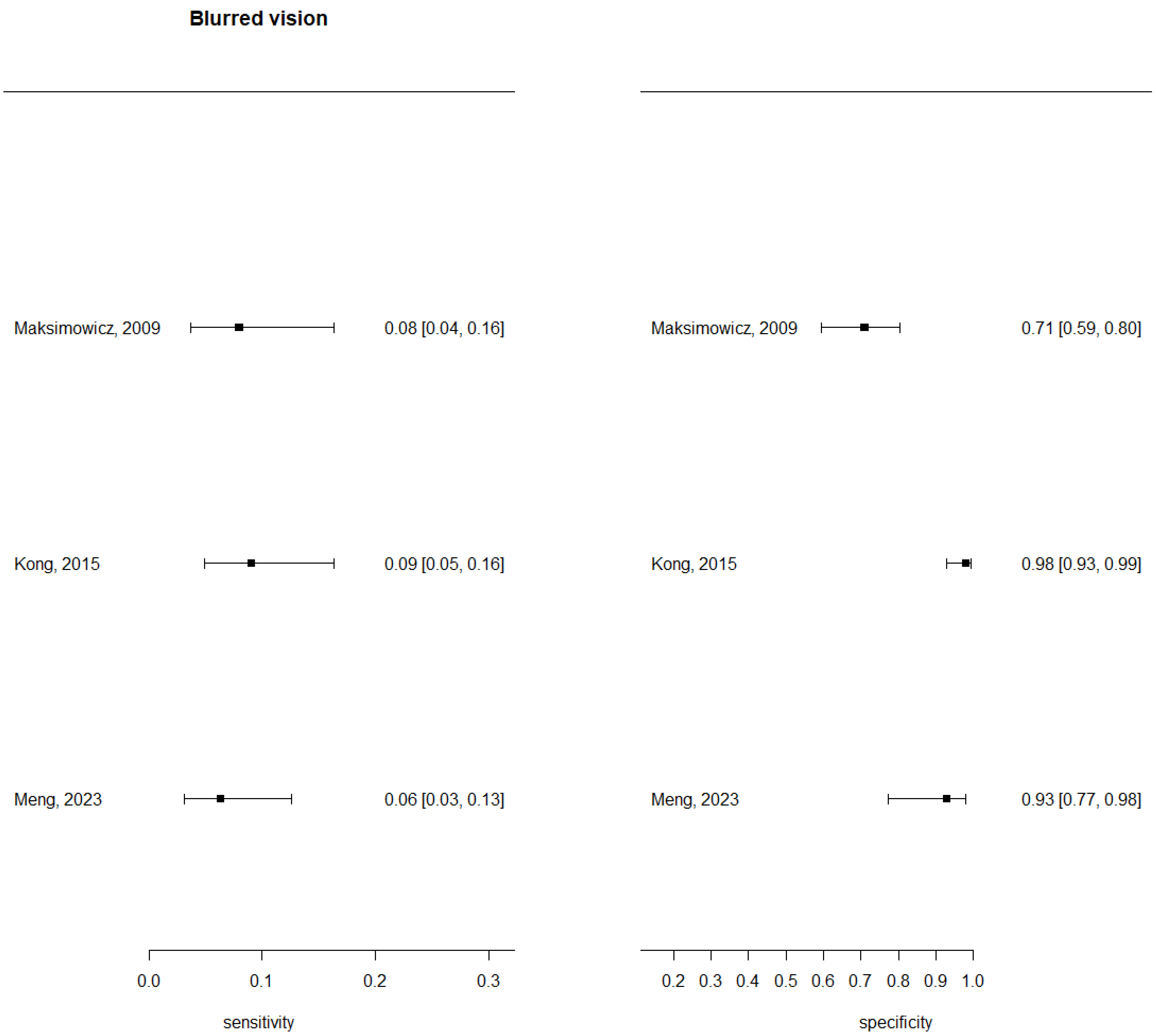
Blurred vision
Maksimowicz, 2009
0.08 [0.04, 0.16]
Kong, 2015
0.09 [0.05, 0.16]
Meng, 2023
0.06 [0.03, 0.13]
0.0
0.1
0.2
0.3
sensitivity
Maksimowicz, 2009
0.71 [0.59, 0.80]
Kong, 2015
0.98 [0.93, 0.99]
Meng, 2023
0.93 [0.77, 0.98]
0.2
0.3
0.4
0.5
0.6
0.7
0.8
0.9
1.0
specificity

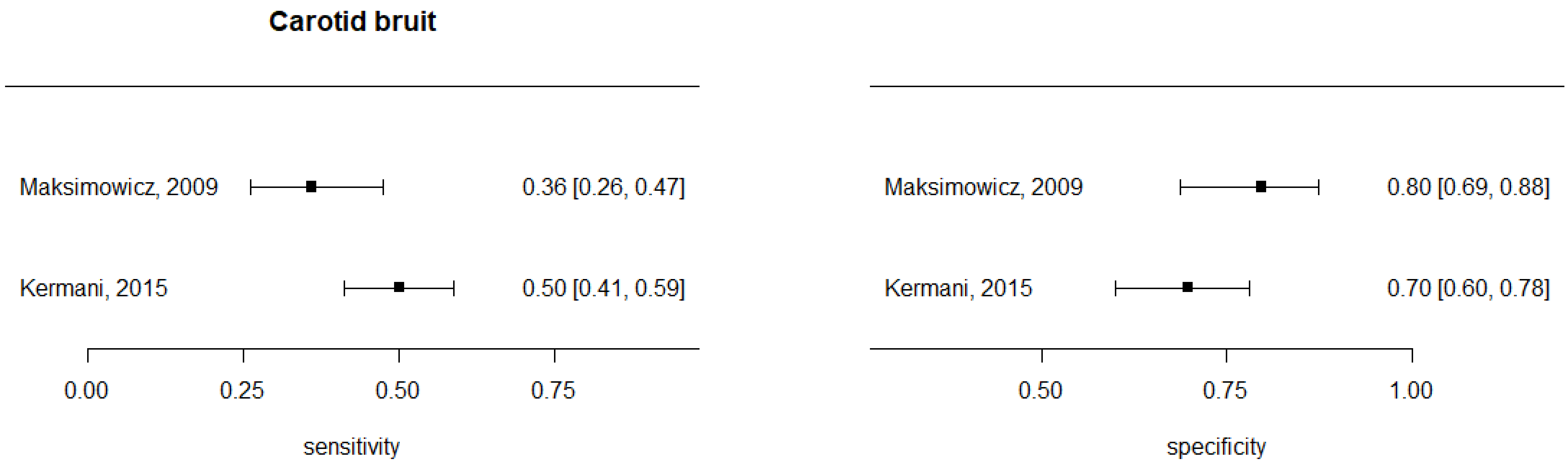

Carotid bruit
Maksimowicz, 2009
0.36 [0.26, 0.47]
Kermani, 2015
0.50 [0.41, 0.59]
0.00
0.25
0.50
0.75
sensitivity
Maksimowicz, 2009
0.80 [0.69, 0.88]
Kermani, 2015
0.70 [0.60, 0.78]
0.50
0.75
1.00
specificity

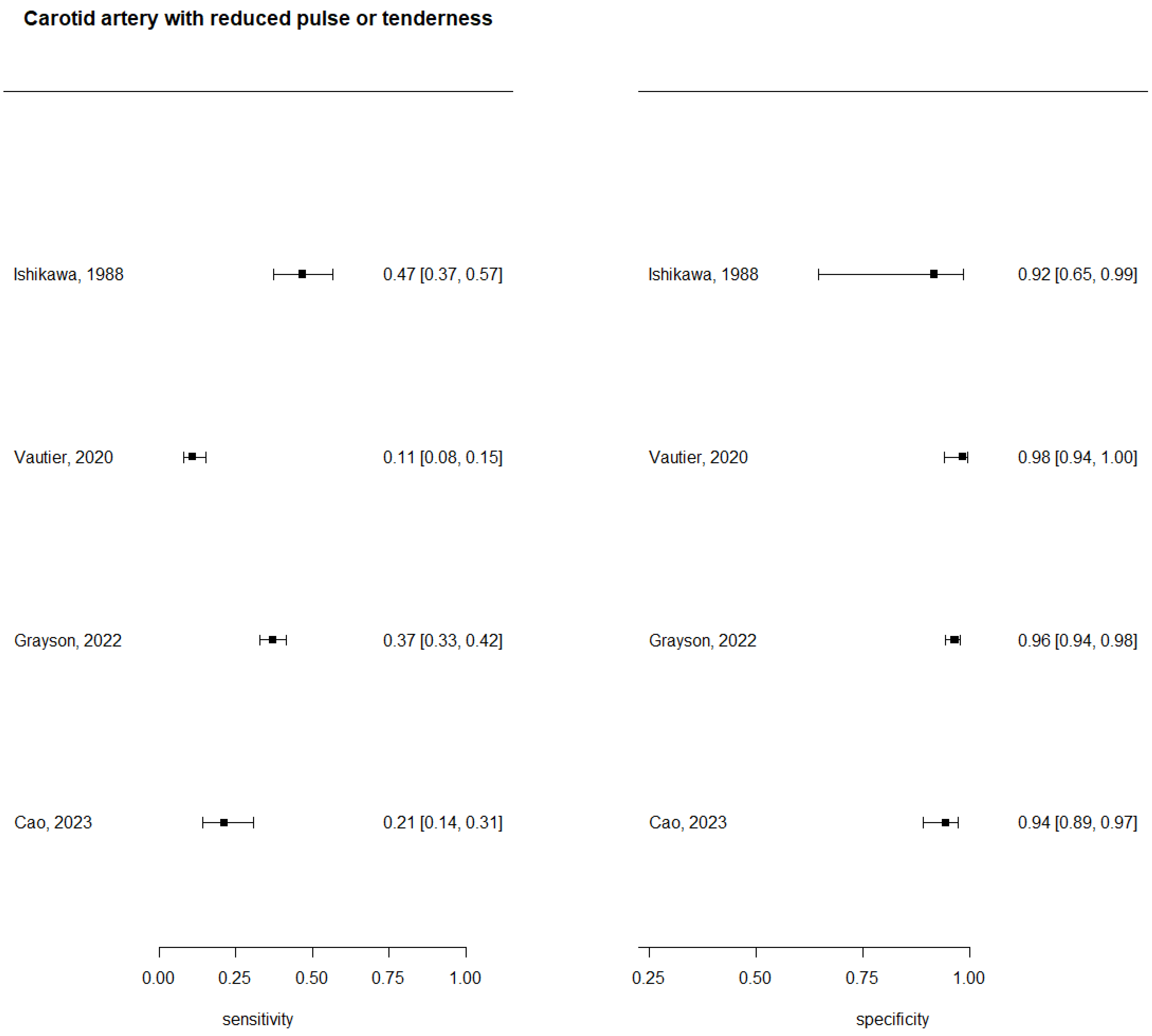
Carotid artery with reduced pulse or tenderness
Ishikawa, 1988
0.47 [0.37, 0.57]
Vautier, 2020
0.11 [0.08, 0.15]
Grayson, 2022
0.37 [0.33, 0.42]
Cao, 2023
0.21 [0.14, 0.31]
0.00
0.25
0.50
0.75
1.00
sensitivity
Ishikawa, 1988
0.92 [0.65, 0.99]
Vautier, 2020
0.98 [0.94, 1.00]
Grayson, 2022
0.96 [0.94, 0.98]
Cao, 2023
0.94 [0.89, 0.97]
0.25
0.50
0.75
1.00
specificity

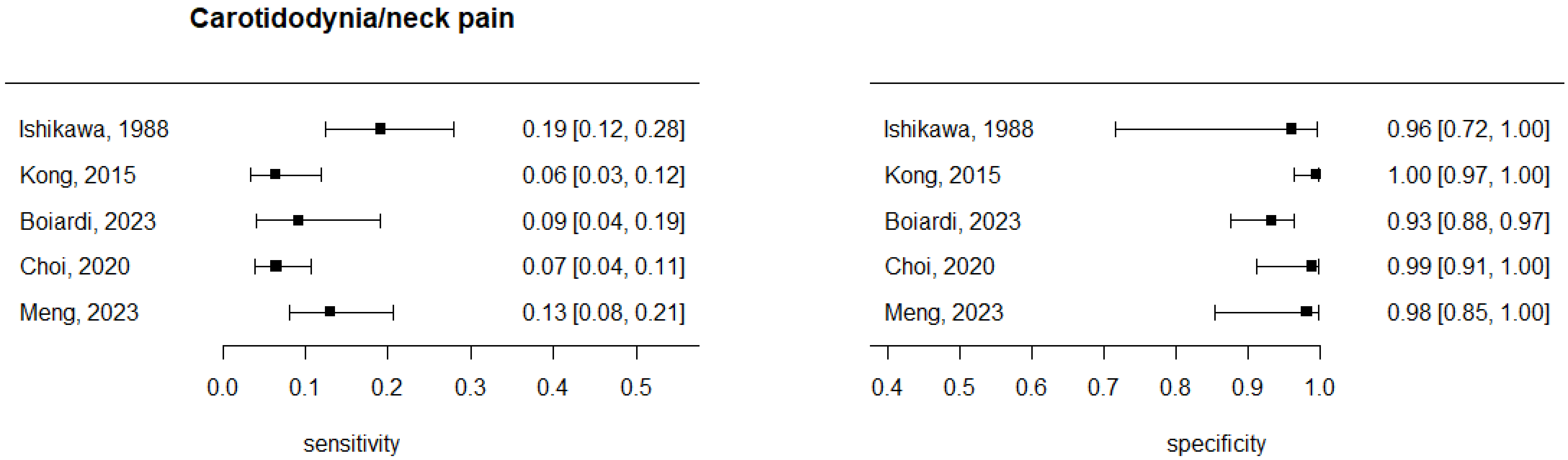
Carotidodynia/neck pain
Ishikawa, 1988
0.19 [0.12, 0.28]
Kong, 2015
0.06 [0.03, 0.12]
Boiardi, 2023
0.09 [0.04, 0.19]
Choi, 2020
0.07 [0.04, 0.11]
Meng, 2023
0.13 [0.08, 0.21]
0.0
0.1
0.2
0.3
0.4
0.5
sensitivity
Ishikawa, 1988
0.96 [0.72, 1.00]
Kong, 2015
1.00 [0.97, 1.00]
Boiardi, 2023
0.93 [0.88, 0.97]
Choi, 2020
0.99 [0.91, 1.00]
Meng, 2023
0.98 [0.85, 1.00]
0.4
0.5
0.6
0.7
0.8
0.9
1.0
specificity

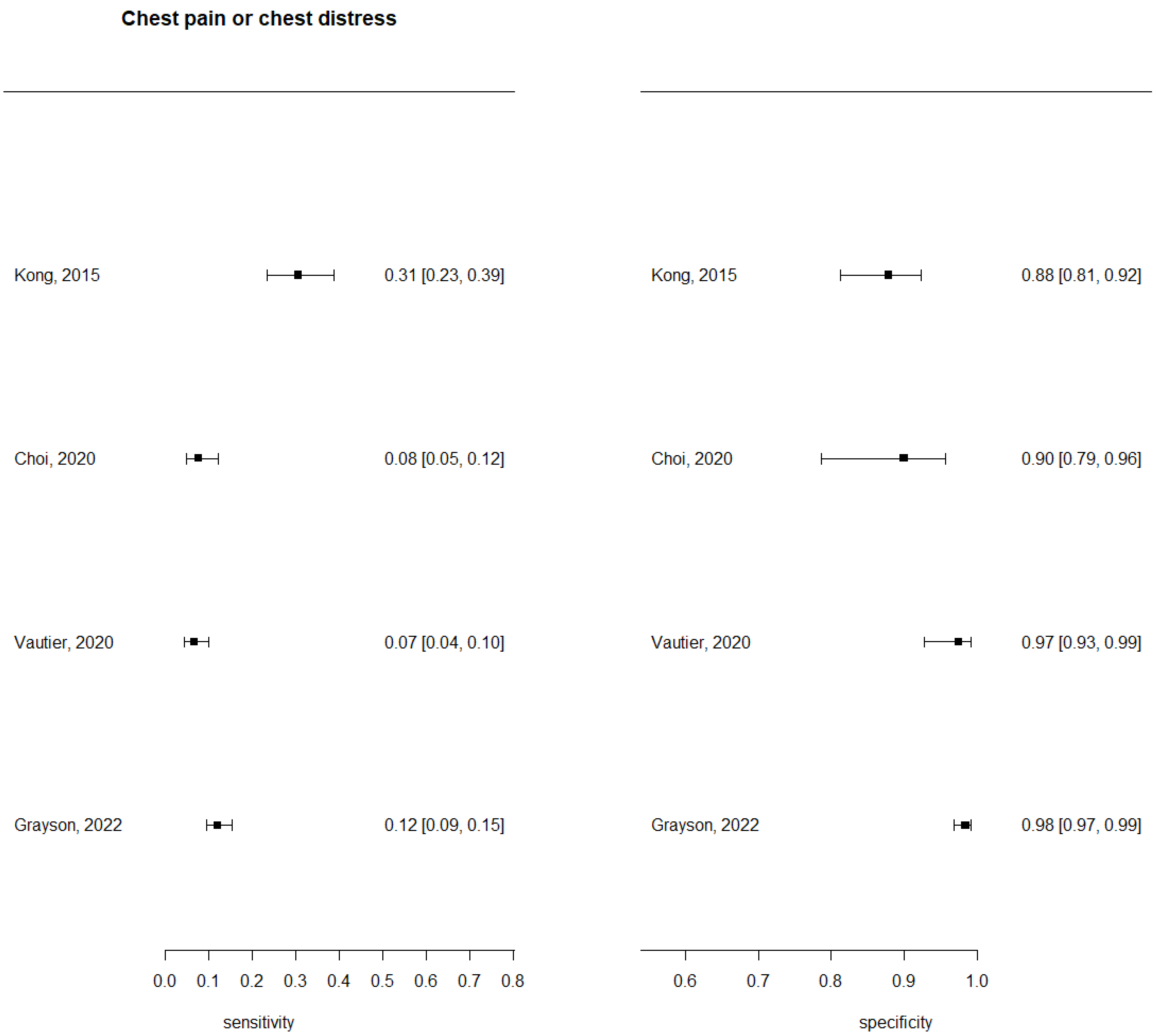
Chest pain or chest distress
Kong, 2015
0.31 [0.23, 0.39]
Choi, 2020
0.08 [0.05, 0.12]
Vautier, 2020
0.07 [0.04, 0.10]
Grayson, 2022
0.12 [0.09, 0.15]
0.0 0.1 0.2 0.3 0.4 0.5 0.6 0.7 0.8
sensitivity
Kong, 2015
0.88 [0.81, 0.92]
Choi, 2020
0.90 [0.79, 0.96]
Vautier, 2020
0.97 [0.93, 0.99]
Grayson, 2022
0.98 [0.97, 0.99]
0.6 0.7 0.8 0.9 1.0
specificity

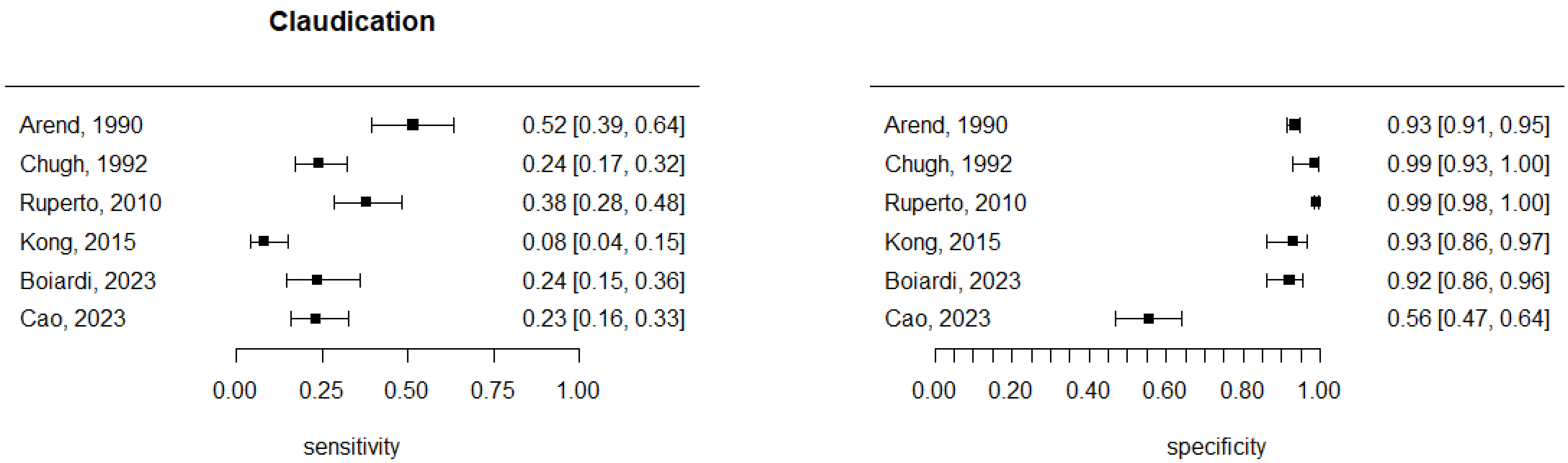
Claudication
Arend, 1990
0.52 [0.39, 0.64]
Chugh, 1992
0.24 [0.17, 0.32]
Ruperto, 2010
0.38 [0.28, 0.48]
Kong, 2015
0.08 [0.04, 0.15]
Boiardi, 2023
0.24 [0.15, 0.36]
Cao, 2023
0.23 [0.16, 0.33]
0.00
0.25
0.50
0.75
1.00
sensitivity
Arend, 1990
0.93 [0.91, 0.95]
Chugh, 1992
0.99 [0.93, 1.00]
Ruperto, 2010
0.99 [0.98, 1.00]
Kong, 2015
0.93 [0.86, 0.97]
Boiardi, 2023
0.92 [0.86, 0.96]
Cao, 2023
0.56 [0.47, 0.64]
0.00
0.20
0.40
0.60
0.80
1.00
specificity

Decreased or absent pulse

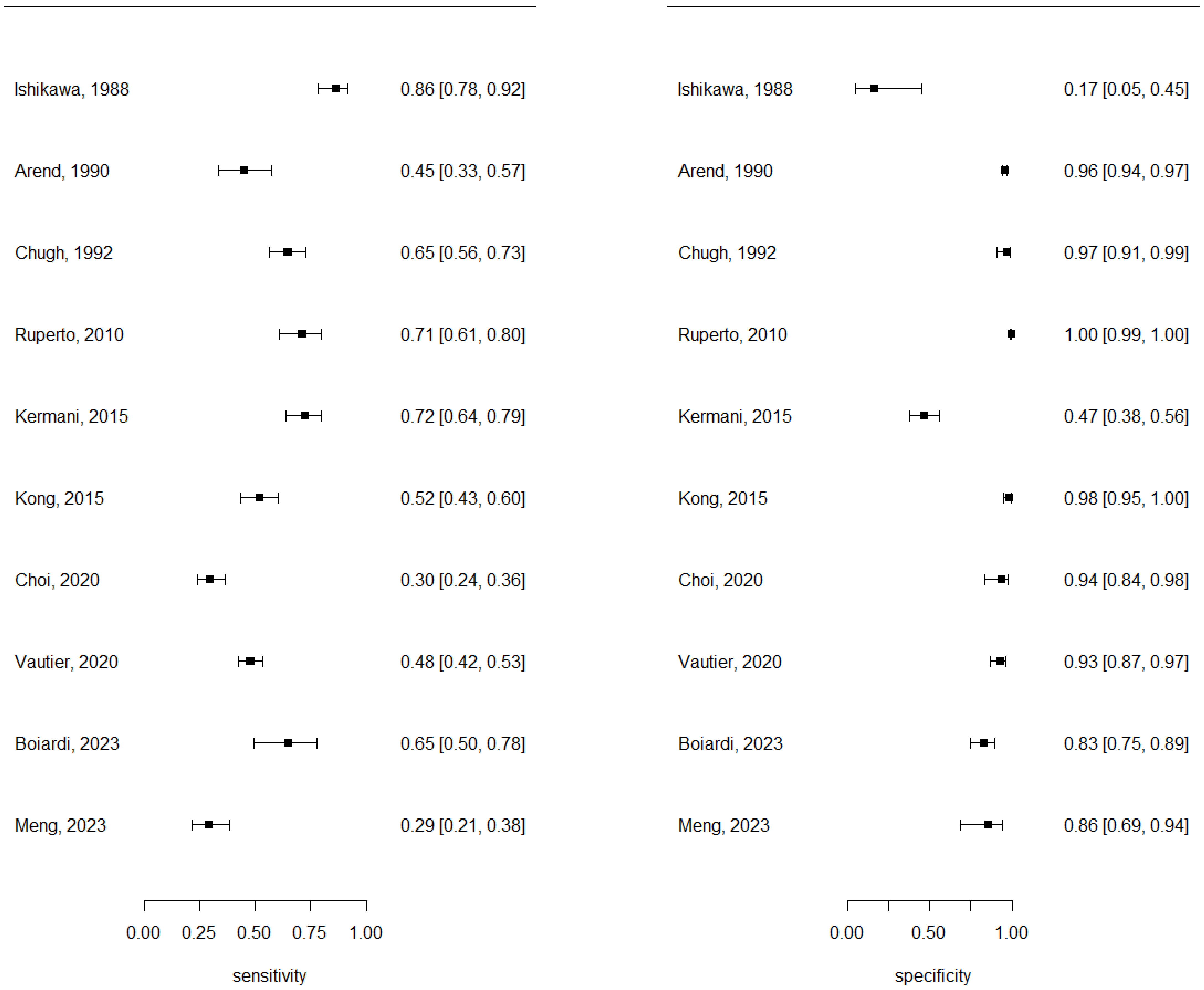

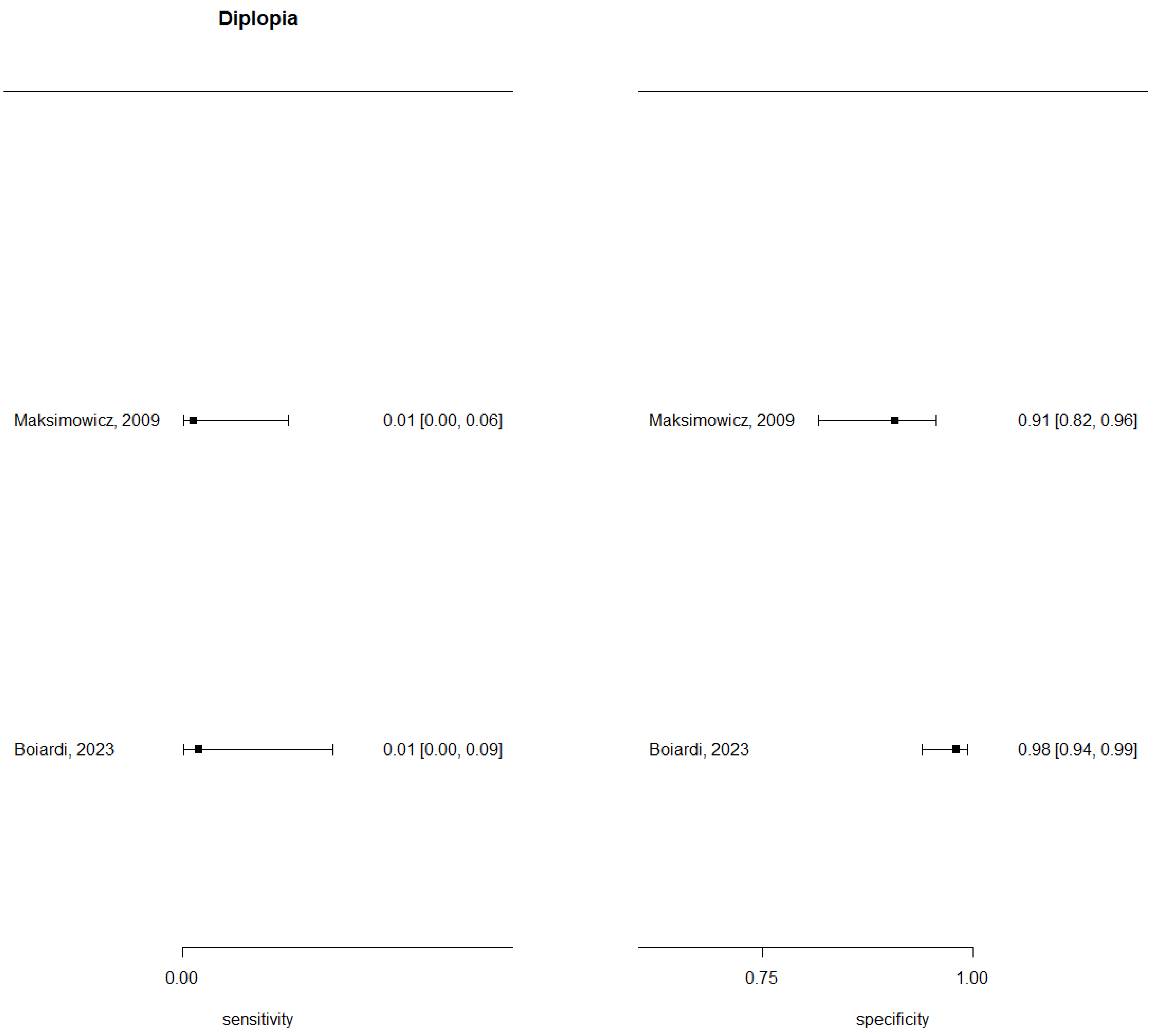
Diplopia
Maksimowicz, 2009
0.01 [0.00, 0.06]
Boiardi, 2023
0.01 [0.00, 0.09]
0.00
sensitivity
Maksimowicz, 2009
0.91 [0.82, 0.96]
Boiardi, 2023
0.98 [0.94, 0.99]
0.75
1.00
specificity

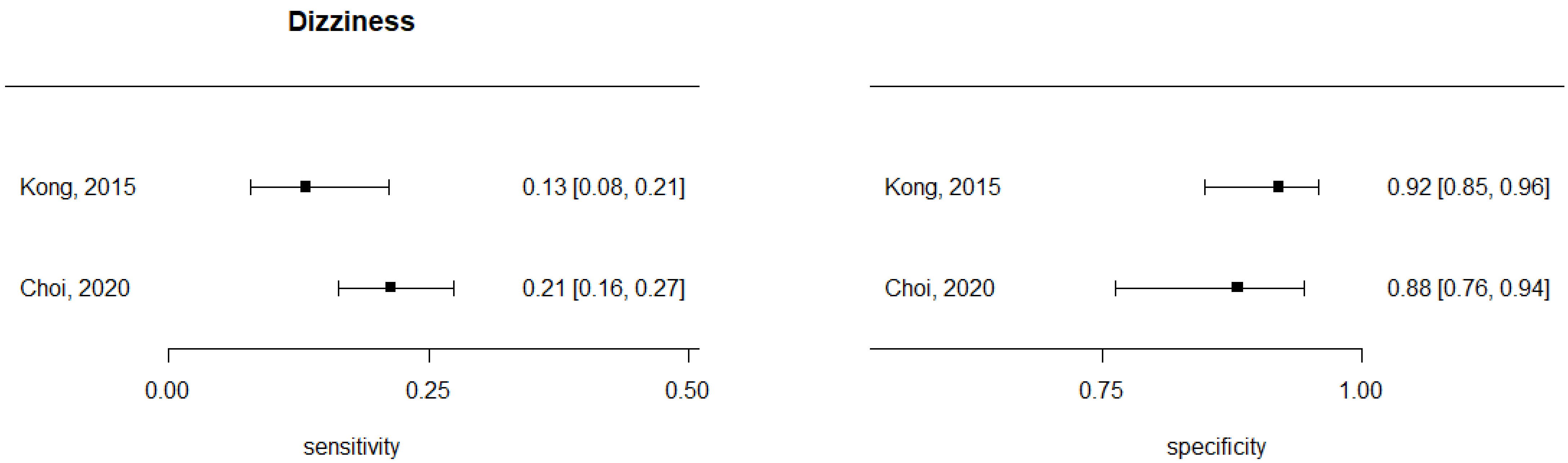

Dizziness
Kong, 2015
0.13 [0.08, 0.21]
Choi, 2020
0.21 [0.16, 0.27]
0.00
0.25
0.50
sensitivity
Kong, 2015
0.92 [0.85, 0.96]
Choi, 2020
0.88 [0.76, 0.94]
0.75
1.00
specificity

Faintness

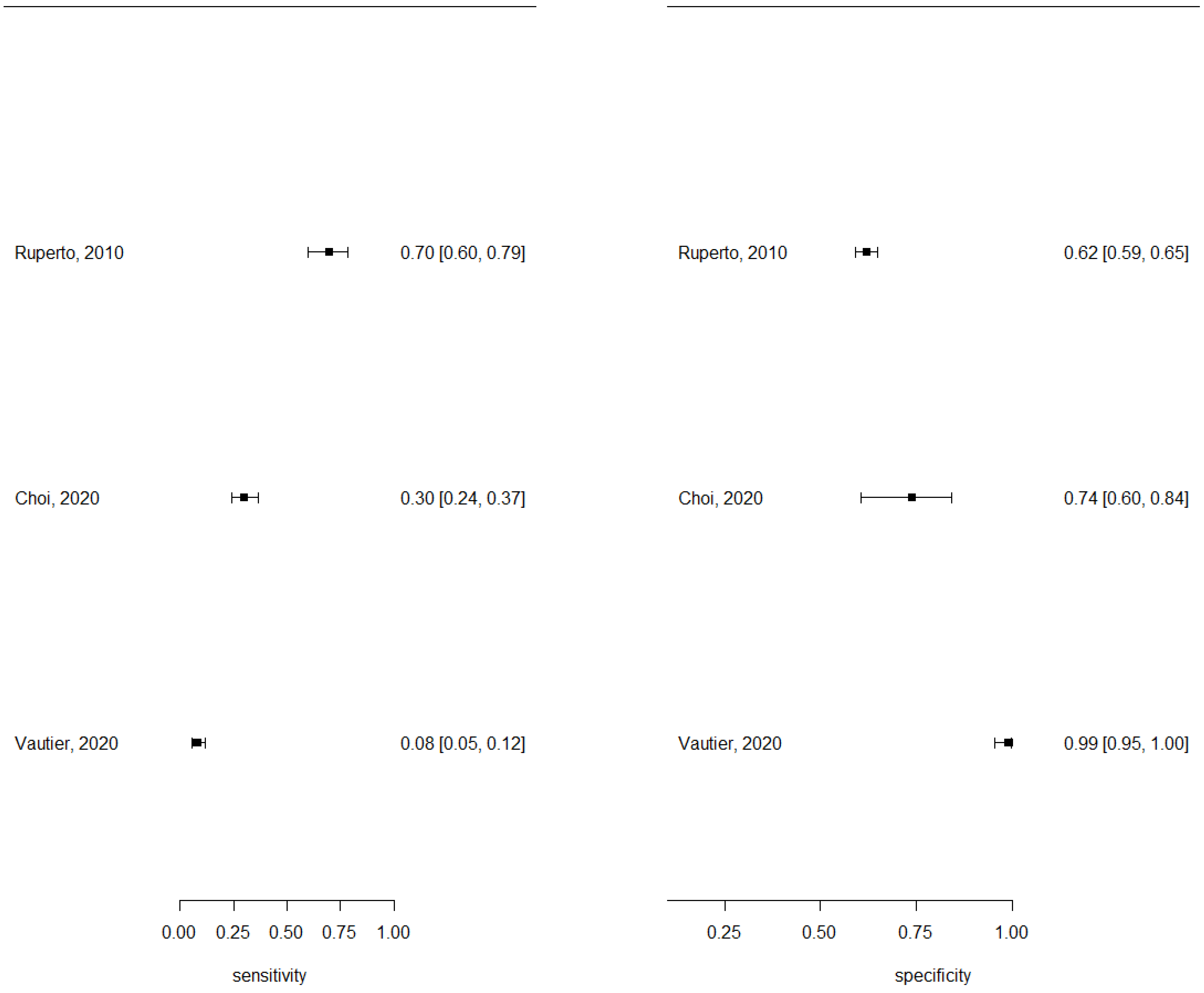
Ruperto, 2010
0.70 [0.60, 0.79]
Choi, 2020
0.30 [0.24, 0.37]
Vautier, 2020
0.08 [0.05, 0.12]
0.00
0.25
0.50
0.75
1.00
sensitivity
Ruperto, 2010
0.62 [0.59, 0.65]
Choi, 2020
0.74 [0.60, 0.84]
Vautier, 2020
0.99 [0.95, 1.00]
0.25
0.50
0.75
1.00
specificity

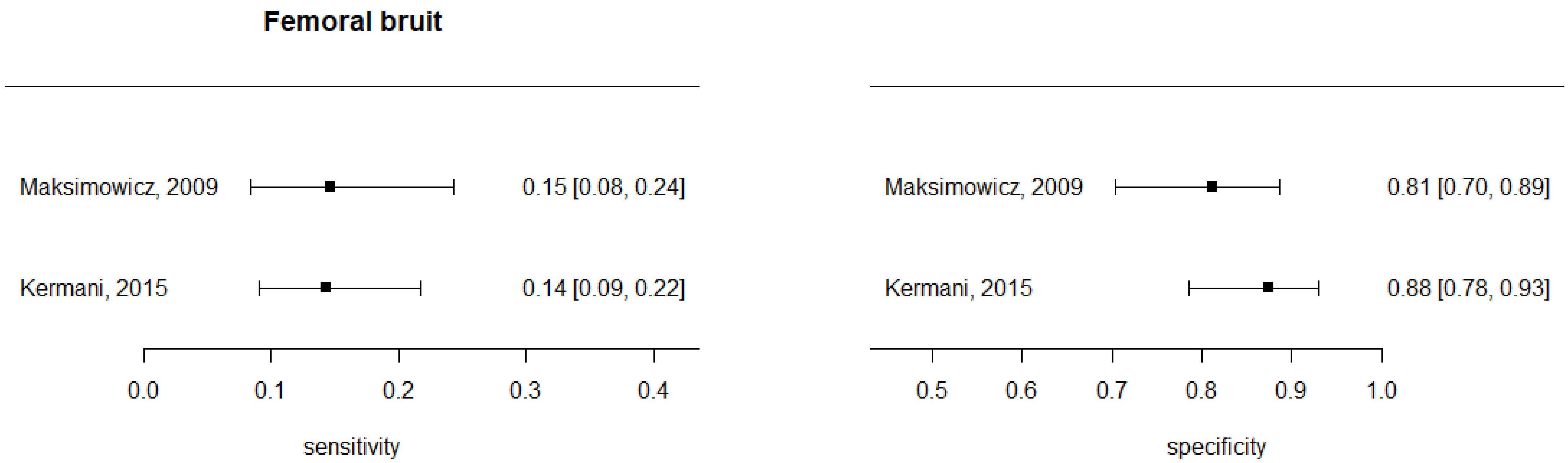
Femoral bruit
Maksimowicz, 2009
0.15 [0.08, 0.24]
Kermani, 2015
0.14 [0.09, 0.22]
0.0
0.1
0.2
0.3
0.4
sensitivity
Maksimowicz, 2009
0.81 [0.70, 0.89]
Kermani, 2015
0.88 [0.78, 0.93]
0.5
0.6
0.7
0.8
0.9
1.0
specificity

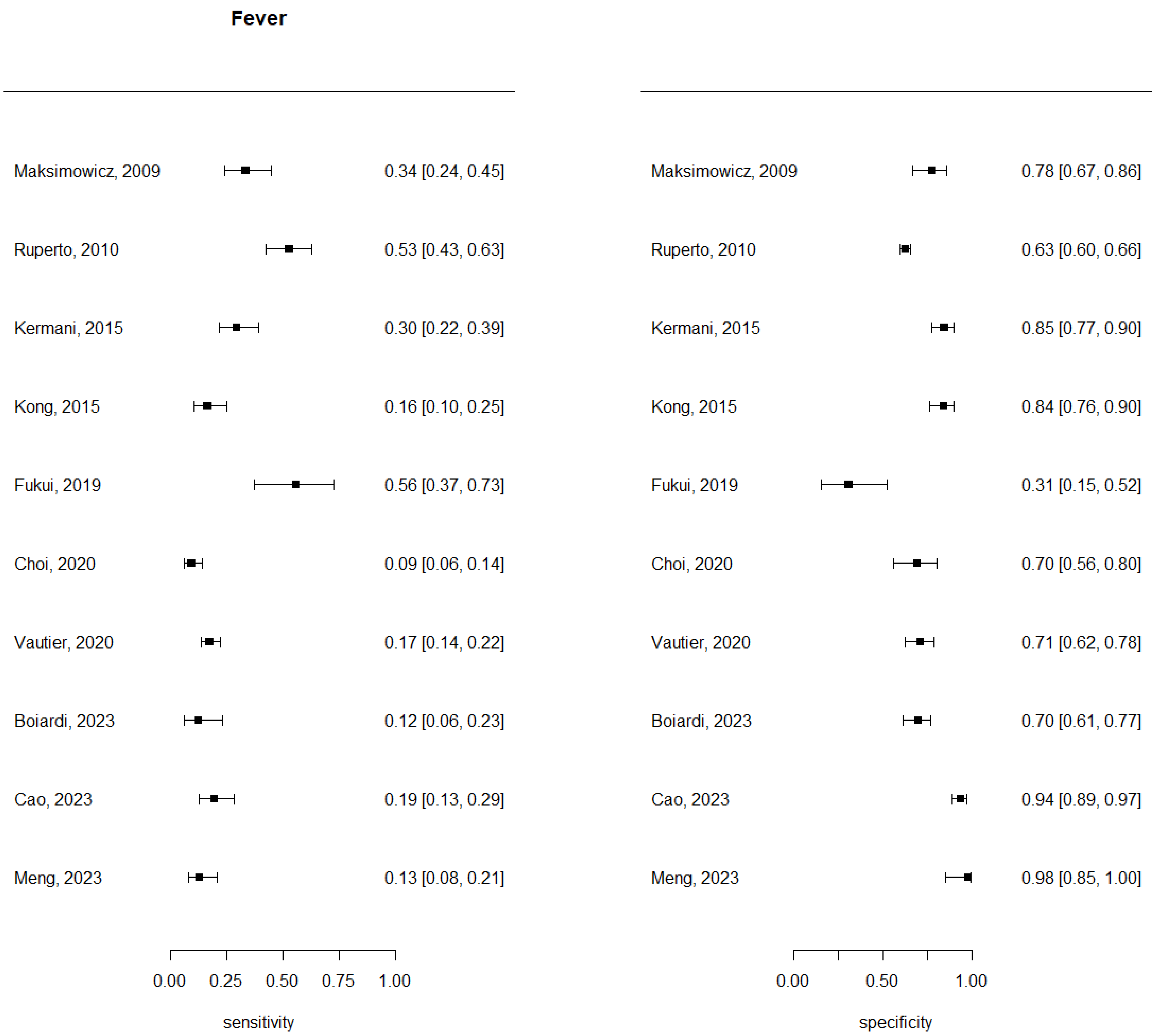
Fever
Maksimowicz, 2009
0.34 [0.24, 0.45]
Ruperto, 2010
0.53 [0.43, 0.63]
Kermani, 2015
0.30 [0.22, 0.39]
Kong, 2015
0.16 [0.10, 0.25]
Fukui, 2019
0.56 [0.37, 0.73]
Choi, 2020
0.09 [0.06, 0.14]
Vautier, 2020
0.17 [0.14, 0.22]
Boiardi, 2023
0.12 [0.06, 0.23]
Cao, 2023
0.19 [0.13, 0.29]
Meng, 2023
0.13 [0.08, 0.21]
0.00
0.25
0.50
0.75
1.00
sensitivity
Maksimowicz, 2009
0.78 [0.67, 0.86]
Ruperto, 2010
0.63 [0.60, 0.66]
Kermani, 2015
0.85 [0.77, 0.90]
Kong, 2015
0.84 [0.76, 0.90]
Fukui, 2019
0.31 [0.15, 0.52]
Choi, 2020
0.70 [0.56, 0.80]
Vautier, 2020
0.71 [0.62, 0.78]
Boiardi, 2023
0.70 [0.61, 0.77]
Cao, 2023
0.94 [0.89, 0.97]
Meng, 2023
0.98 [0.85, 1.00]
0.00
0.50
1.00
specificity

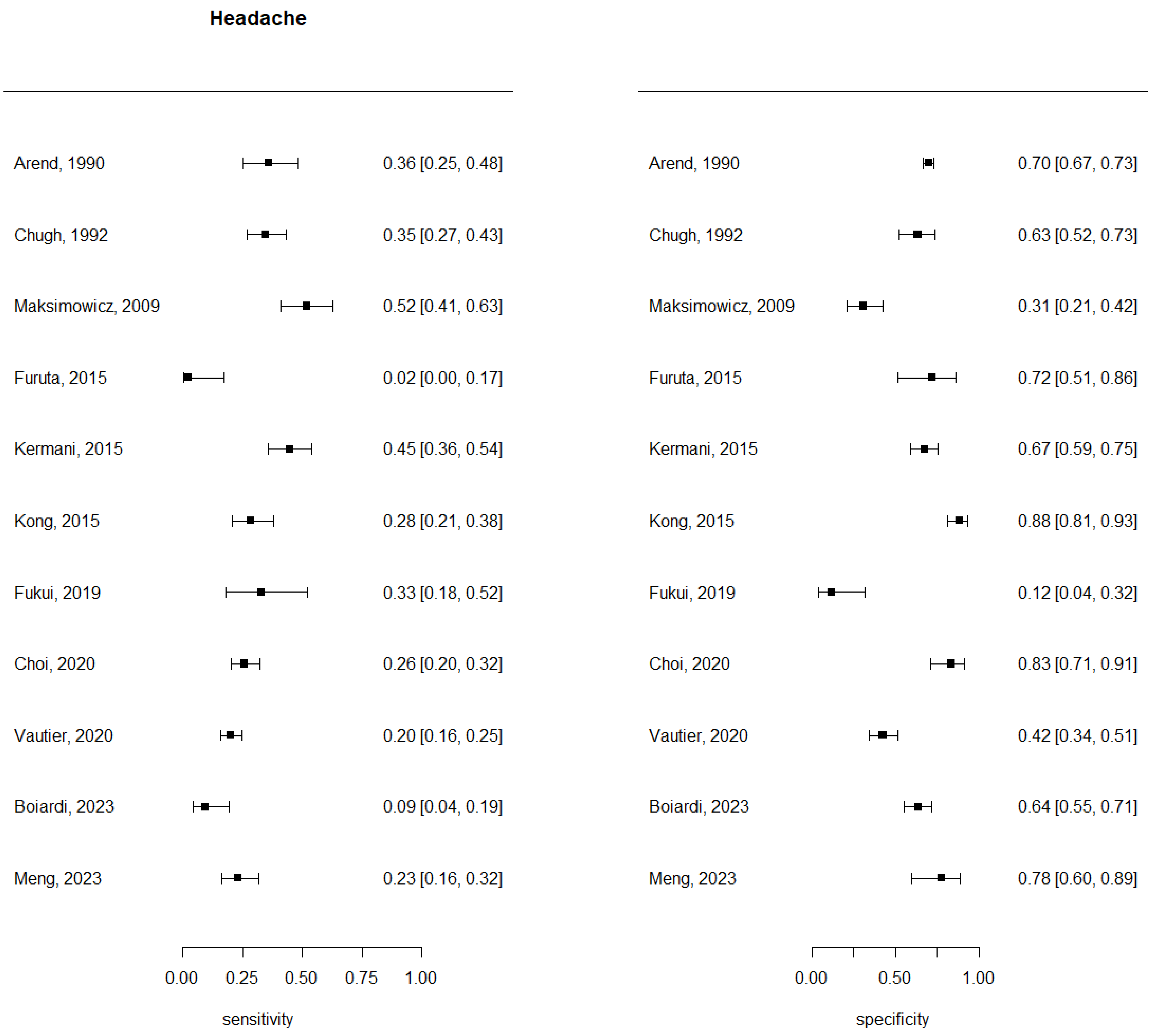
Headache
Arend, 1990
0.36 [0.25, 0.48]
Chugh, 1992
0.35 [0.27, 0.43]
Maksimowicz, 2009
0.52 [0.41, 0.63]
Furuta, 2015
0.02 [0.00, 0.17]
Kermani, 2015
0.45 [0.36, 0.54]
Kong, 2015
0.28 [0.21, 0.38]
Fukui, 2019
0.33 [0.18, 0.52]
Choi, 2020
0.26 [0.20, 0.32]
Vautier, 2020
0.20 [0.16, 0.25]
Boiardi, 2023
0.09 [0.04, 0.19]
Meng, 2023
0.23 [0.16, 0.32]
0.00
0.25
0.50
0.75
1.00
sensitivity
Arend, 1990
0.70 [0.67, 0.73]
Chugh, 1992
0.63 [0.52, 0.73]
Maksimowicz, 2009
0.31 [0.21, 0.42]
Furuta, 2015
0.72 [0.51, 0.86]
Kermani, 2015
0.67 [0.59, 0.75]
Kong, 2015
0.88 [0.81, 0.93]
Fukui, 2019
0.12 [0.04, 0.32]
Choi, 2020
0.83 [0.71, 0.91]
Vautier, 2020
0.42 [0.34, 0.51]
Boiardi, 2023
0.64 [0.55, 0.71]
Meng, 2023
0.78 [0.60, 0.89]
0.00
0.50
1.00
specificity

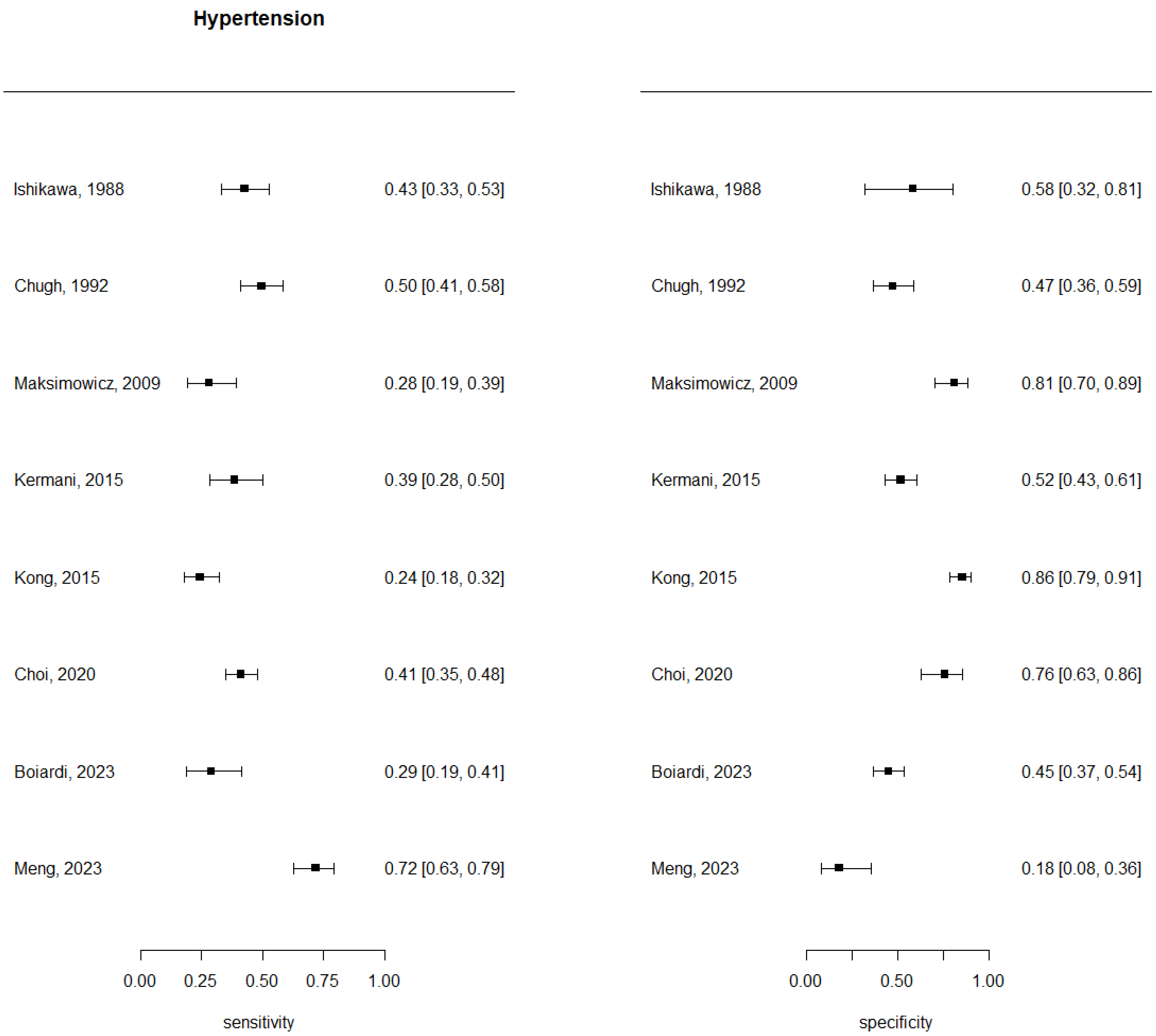

Hypertension
Ishikawa, 1988
0.43 [0.33, 0.53]
Chugh, 1992
0.50 [0.41, 0.58]
Maksimowicz, 2009
0.28 [0.19, 0.39]
Kermani, 2015
0.39 [0.28, 0.50]
Kong, 2015
0.24 [0.18, 0.32]
Choi, 2020
0.41 [0.35, 0.48]
Boiardi, 2023
0.29 [0.19, 0.41]
Meng, 2023
0.72 [0.63, 0.79]
0.00
0.25
0.50
0.75
1.00
sensitivity
Ishikawa, 1988
0.58 [0.32, 0.81]
Chugh, 1992
0.47 [0.36, 0.59]
Maksimowicz, 2009
0.81 [0.70, 0.89]
Kermani, 2015
0.52 [0.43, 0.61]
Kong, 2015
0.86 [0.79, 0.91]
Choi, 2020
0.76 [0.63, 0.86]
Boiardi, 2023
0.45 [0.37, 0.54]
Meng, 2023
0.18 [0.08, 0.36]
0.00
0.50
1.00
specificity

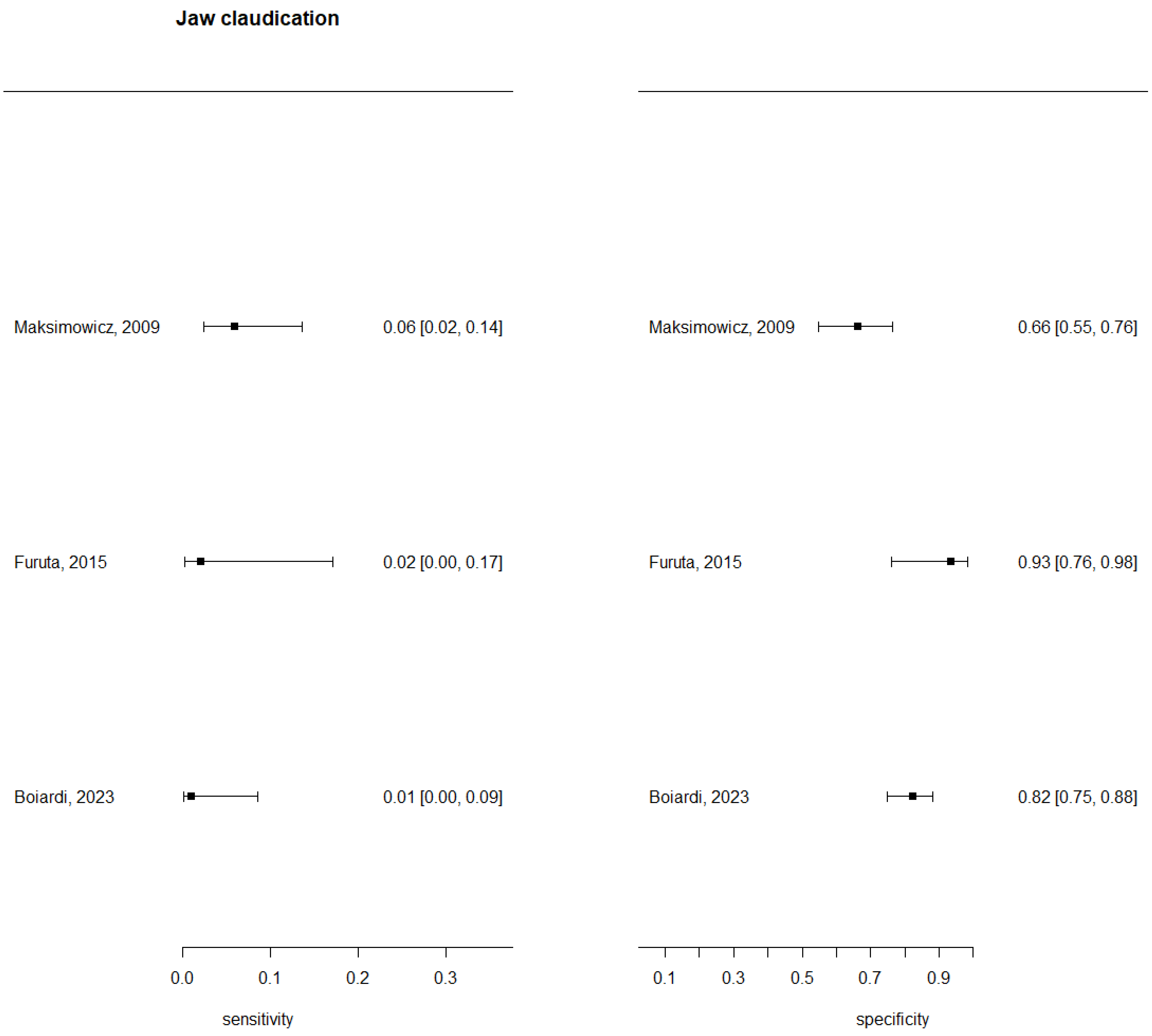
Jaw claudication
Maksimowicz, 2009
0.06 [0.02, 0.14]
Furuta, 2015
0.02 [0.00, 0.17]
Boiardi, 2023
0.01 [0.00, 0.09]
0.0
0.1
0.2
0.3
sensitivity
Maksimowicz, 2009
0.66 [0.55, 0.76]
Furuta, 2015
0.93 [0.76, 0.98]
Boiardi, 2023
0.82 [0.75, 0.88]
0.1
0.3
0.5
0.7
0.9
specificity

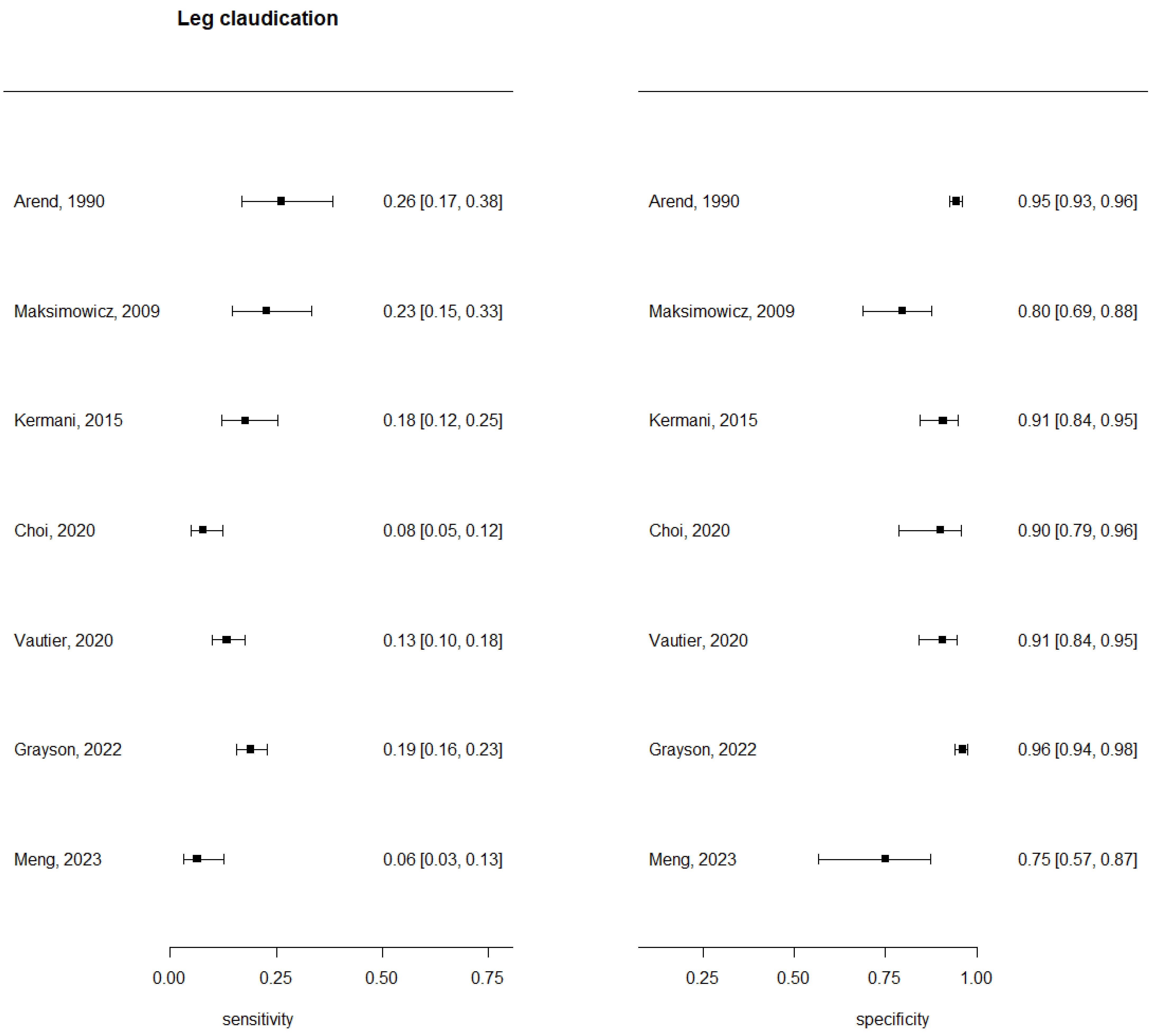
Leg claudication
Arend, 1990
0.26 [0.17, 0.38]
Maksimowicz, 2009
0.23 [0.15, 0.33]
Kermani, 2015
0.18 [0.12, 0.25]
Choi, 2020
0.08 [0.05, 0.12]
Vautier, 2020
0.13 [0.10, 0.18]
Grayson, 2022
0.19 [0.16, 0.23]
Meng, 2023
0.06 [0.03, 0.13]
0.00
0.25
0.50
0.75
sensitivity
Arend, 1990
0.95 [0.93, 0.96]
Maksimowicz, 2009
0.80 [0.69, 0.88]
Kermani, 2015
0.91 [0.84, 0.95]
Choi, 2020
0.90 [0.79, 0.96]
Vautier, 2020
0.91 [0.84, 0.95]
Grayson, 2022
0.96 [0.94, 0.98]
Meng, 2023
0.75 [0.57, 0.87]
0.25
0.50
0.75
1.00
specificity

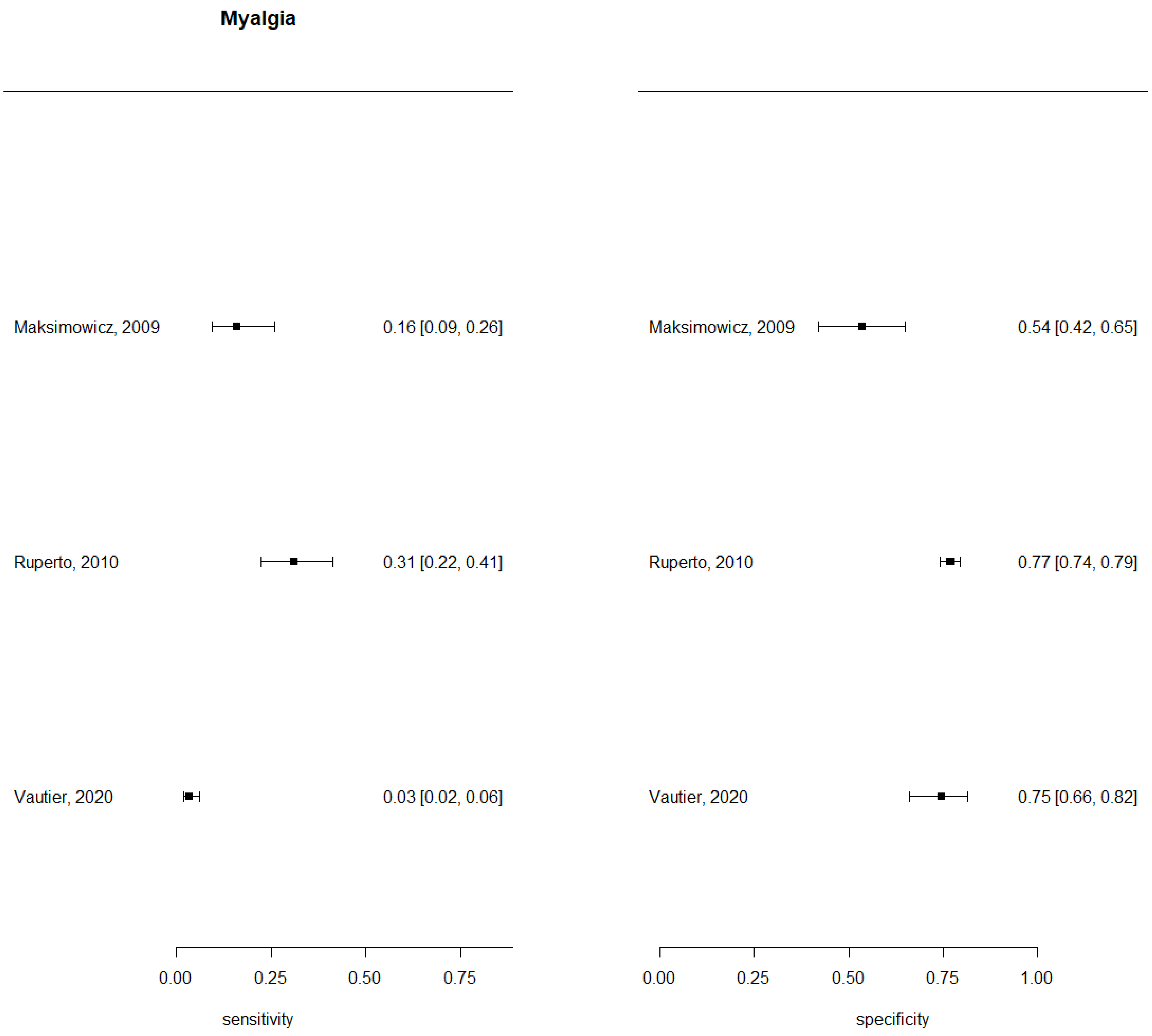

Myalgia
Maksimowicz, 2009
0.16 [0.09, 0.26]
Ruperto, 2010
0.31 [0.22, 0.41]
Vautier, 2020
0.03 [0.02, 0.06]
0.00
0.25
0.50
0.75
sensitivity
Maksimowicz, 2009
0.54 [0.42, 0.65]
Ruperto, 2010
0.77 [0.74, 0.79]
Vautier, 2020
0.75 [0.66, 0.82]
0.00
0.25
0.50
0.75
1.00
specificity

Myocardial infarction

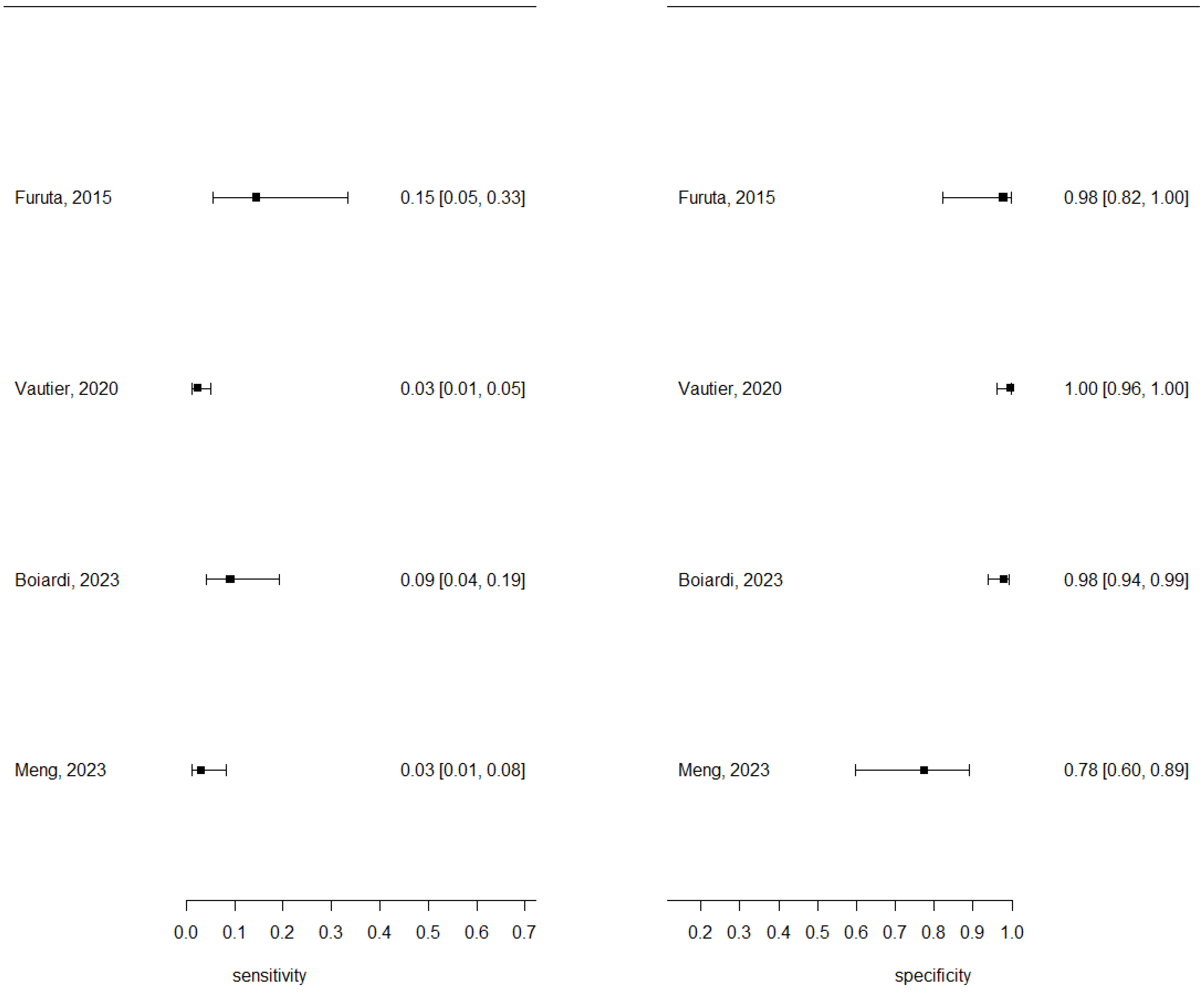

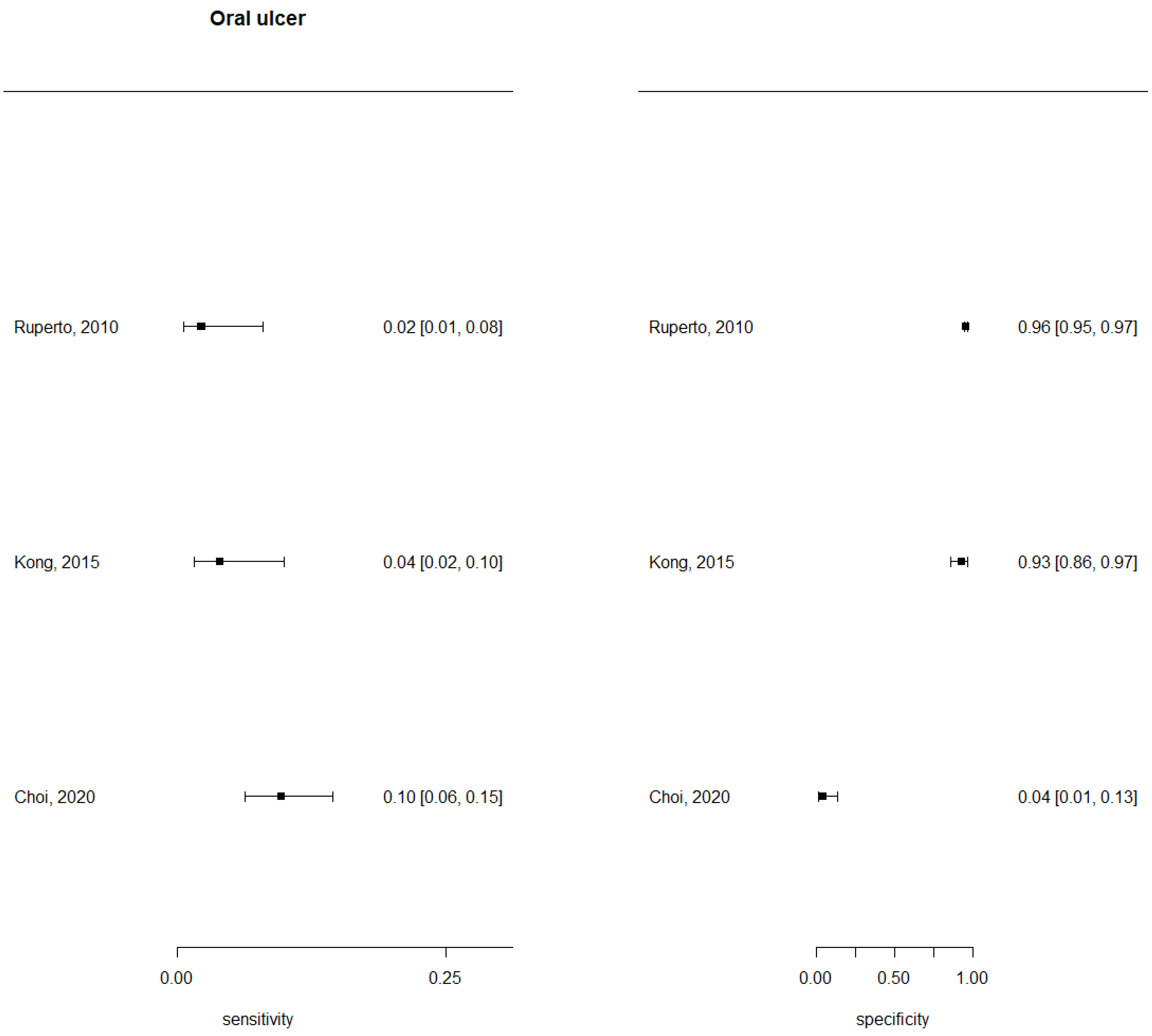

Oral ulcer
Ruperto, 2010
0.02 [0.01, 0.08]
Kong, 2015
0.04 [0.02, 0.10]
Choi, 2020
0.10 [0.06, 0.15]
0.00
0.25
sensitivity
Ruperto, 2010
0.96 [0.95, 0.97]
Kong, 2015
0.93 [0.86, 0.97]
Choi, 2020
0.04 [0.01, 0.13]
0.00
0.50
1.00
specificity

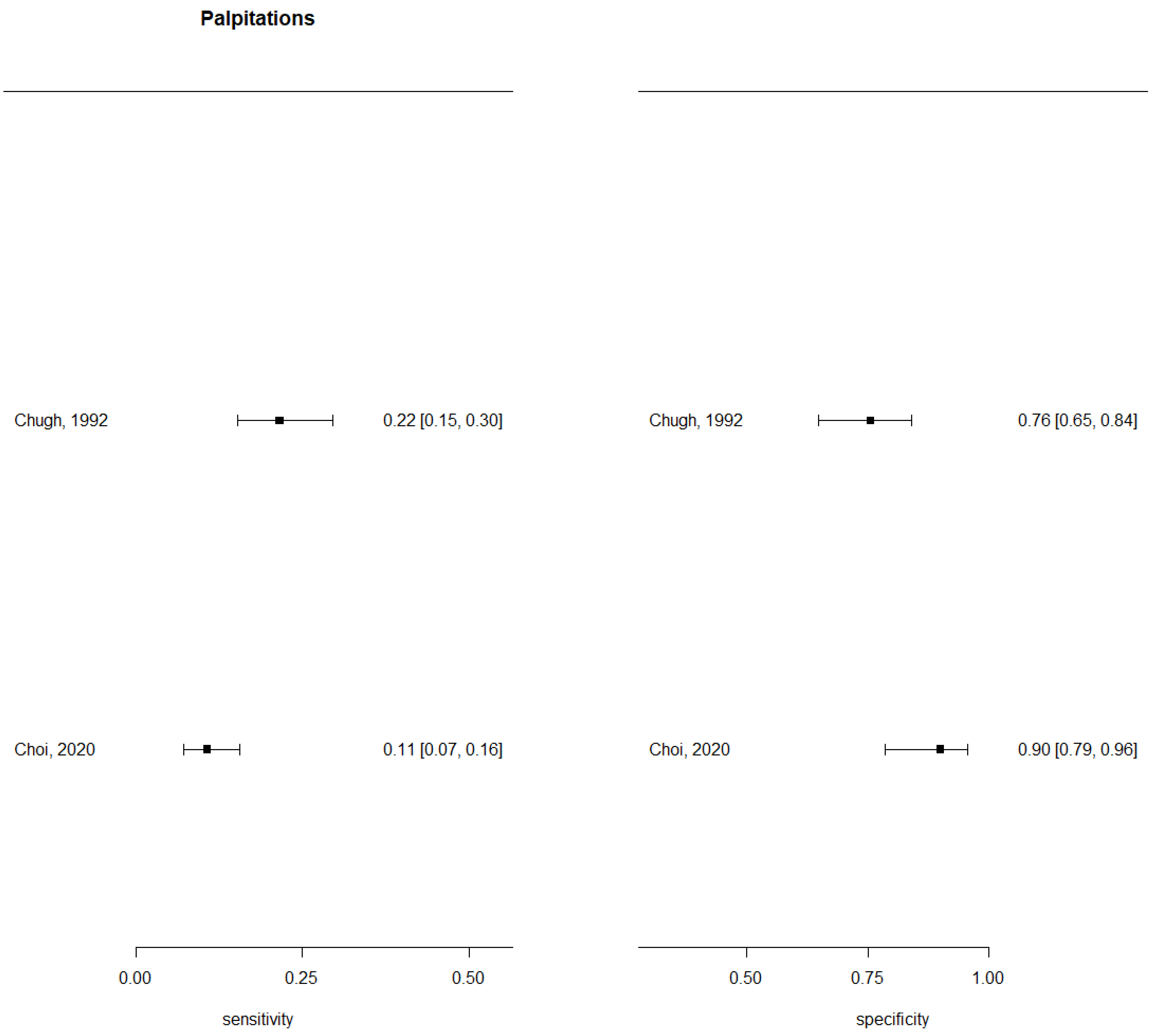
Palpitations
Chugh, 1992
0.22 [0.15, 0.30]
Choi, 2020
0.11 [0.07, 0.16]
0.00
0.25
0.50
sensitivity
Chugh, 1992
0.76 [0.65, 0.84]
Choi, 2020
0.90 [0.79, 0.96]
0.50
0.75
1.00
specificity

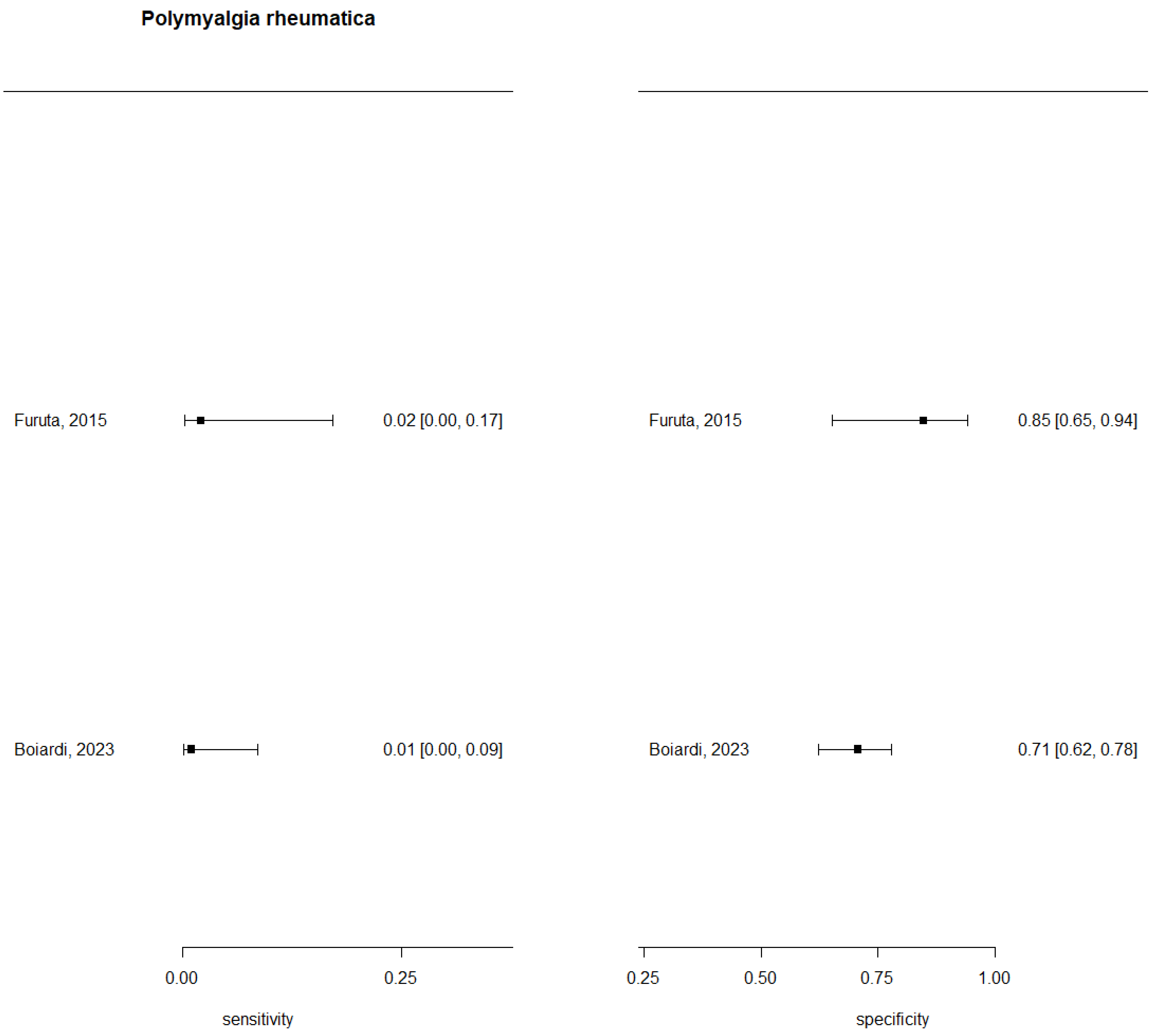

Polymyalgia rheumatica
Furuta, 2015
0.02 [0.00, 0.17]
Boiardi, 2023
0.01 [0.00, 0.09]
0.00
0.25
sensitivity
Furuta, 2015
0.85 [0.65, 0.94]
Boiardi, 2023
0.71 [0.62, 0.78]
0.25
0.50
0.75
1.00
specificity

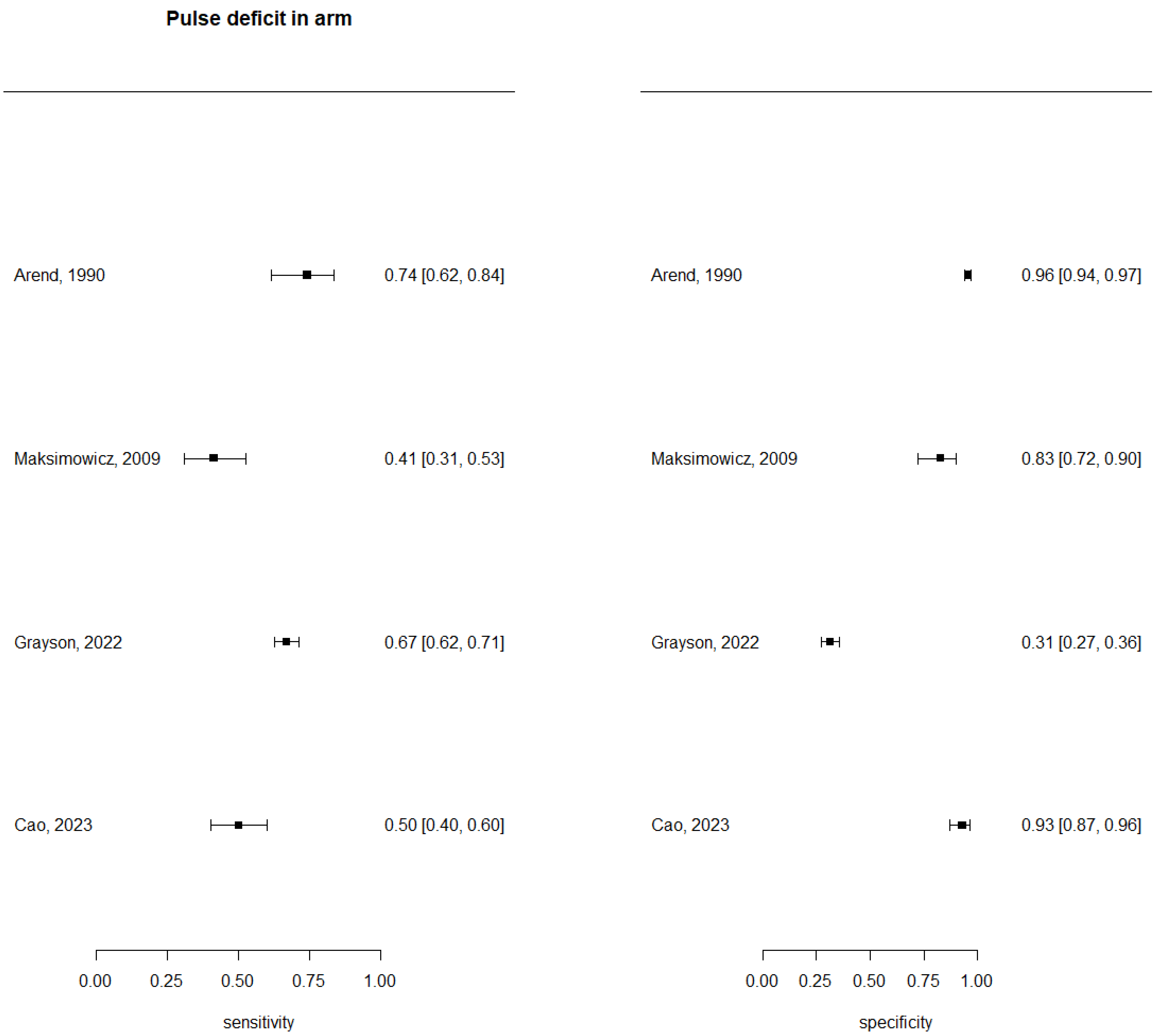
Pulse deficit in arm
Arend, 1990
0.74 [0.62, 0.84]
Maksimowicz, 2009
0.41 [0.31, 0.53]
Grayson, 2022
0.67 [0.62, 0.71]
Cao, 2023
0.50 [0.40, 0.60]
0.00
0.25
0.50
0.75
1.00
sensitivity
Arend, 1990
0.96 [0.94, 0.97]
Maksimowicz, 2009
0.83 [0.72, 0.90]
Grayson, 2022
0.31 [0.27, 0.36]
Cao, 2023
0.93 [0.87, 0.96]
0.00
0.25
0.50
0.75
1.00
specificity

Scalp tenderness

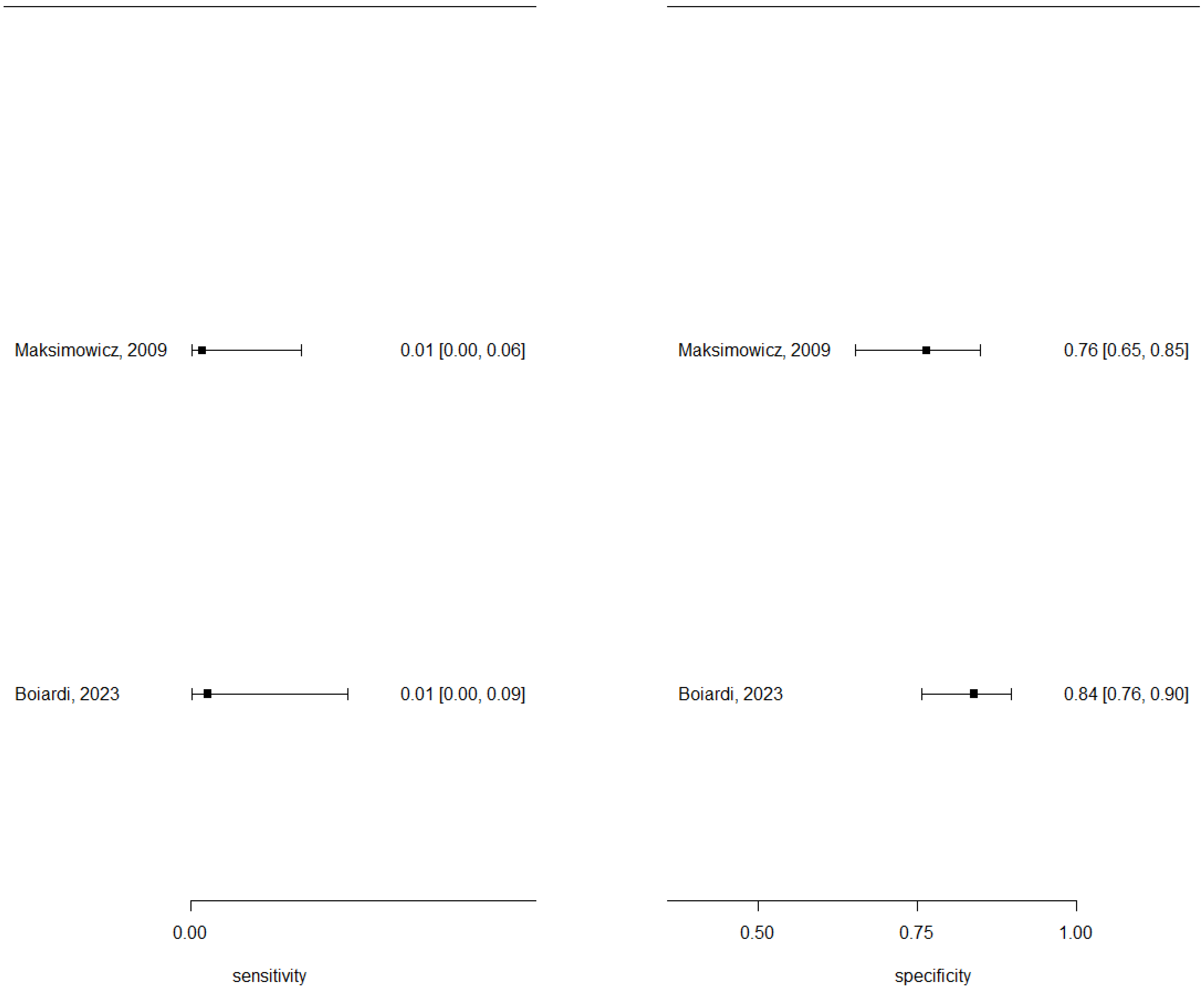

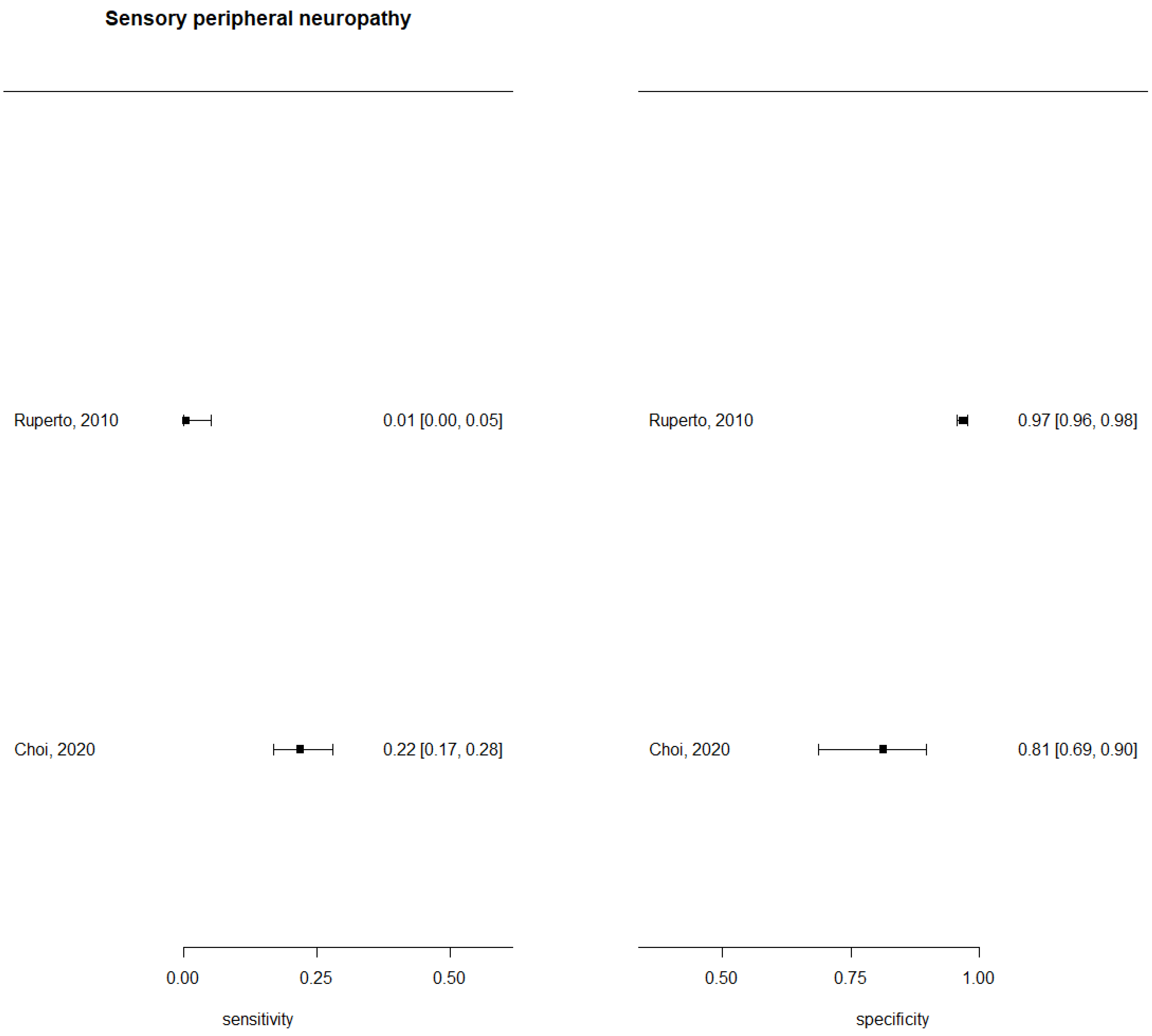
Sensory peripheral neuropathy
Ruperto, 2010
0.01 [0.00, 0.05]
Choi, 2020
0.22 [0.17, 0.28]
0.00
0.25
0.50
sensitivity
Ruperto, 2010
0.97 [0.96, 0.98]
Choi, 2020
0.81 [0.69, 0.90]
0.50
0.75
1.00
specificity

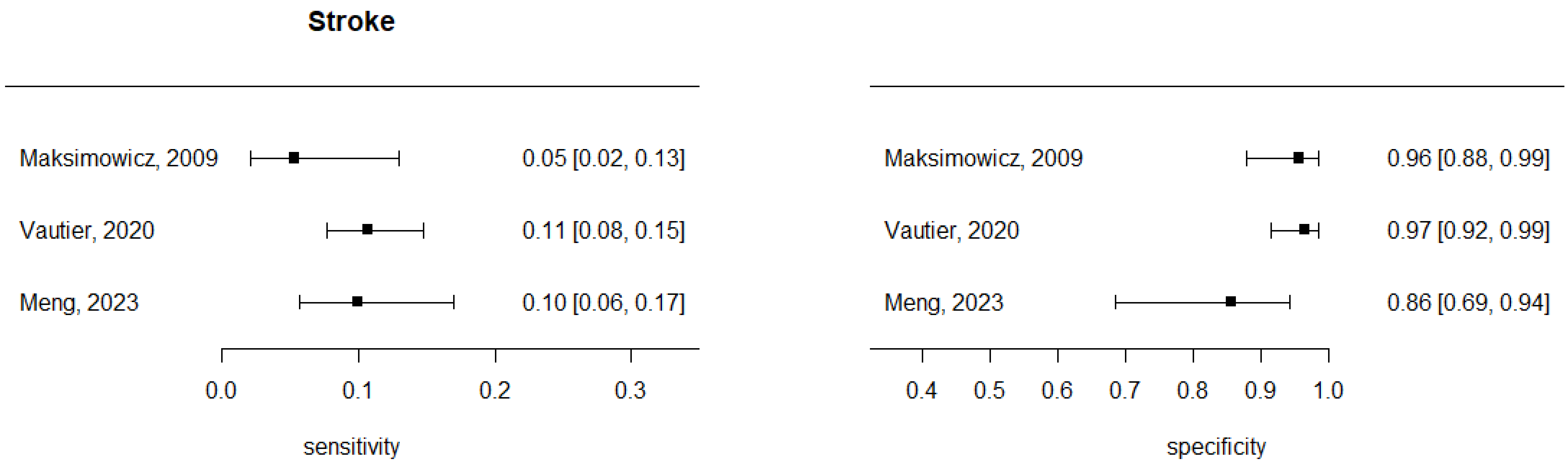
Stroke
Maksimowicz, 2009
Vautier, 2020
Meng, 2023
0.05 [0.02, 0.13]
0.11 [0.08, 0.15]
0.10 [0.06, 0.17]
0.0
0.1
0.2
0.3
sensitivity
Maksimowicz, 2009
Vautier, 2020
Meng, 2023
0.96 [0.88, 0.99]
0.97 [0.92, 0.99]
0.86 [0.69, 0.94]
0.4
0.5
0.6
0.7
0.8
0.9
1.0
specificity

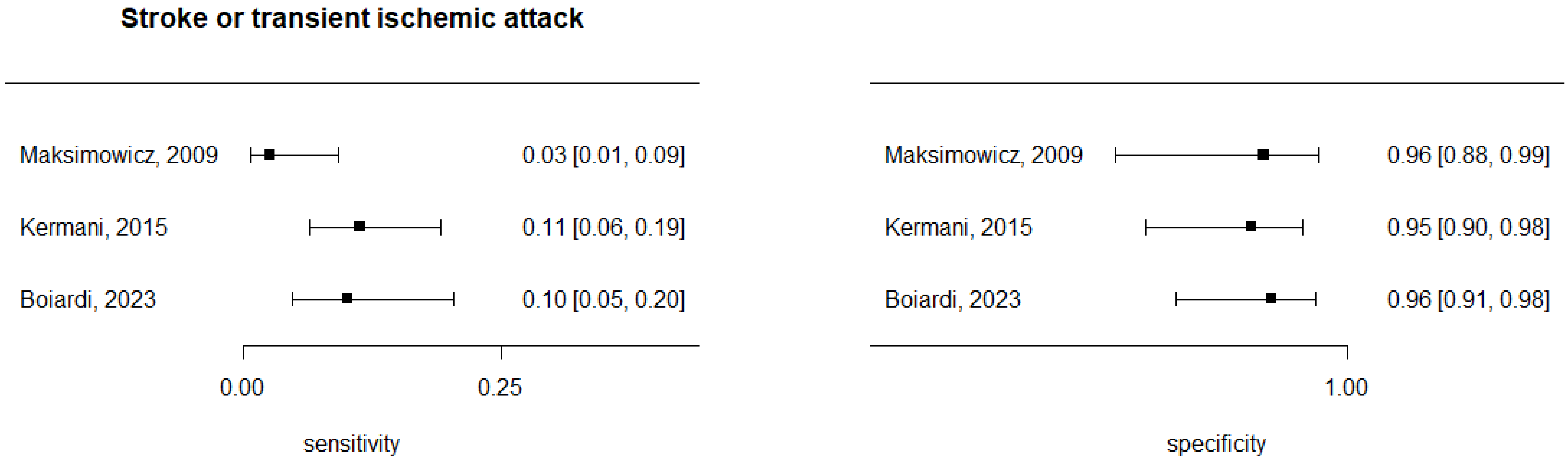
Stroke or transient ischemic attack
Maksimowicz, 2009
Kermani, 2015
Boiardi, 2023
0.03 [0.01, 0.09]
0.11 [0.06, 0.19]
0.10 [0.05, 0.20]
0.00
0.25
sensitivity
Maksimowicz, 2009
Kermani, 2015
Boiardi, 2023
0.96 [0.88, 0.99]
0.95 [0.90, 0.98]
0.96 [0.91, 0.98]
1.00
specificity

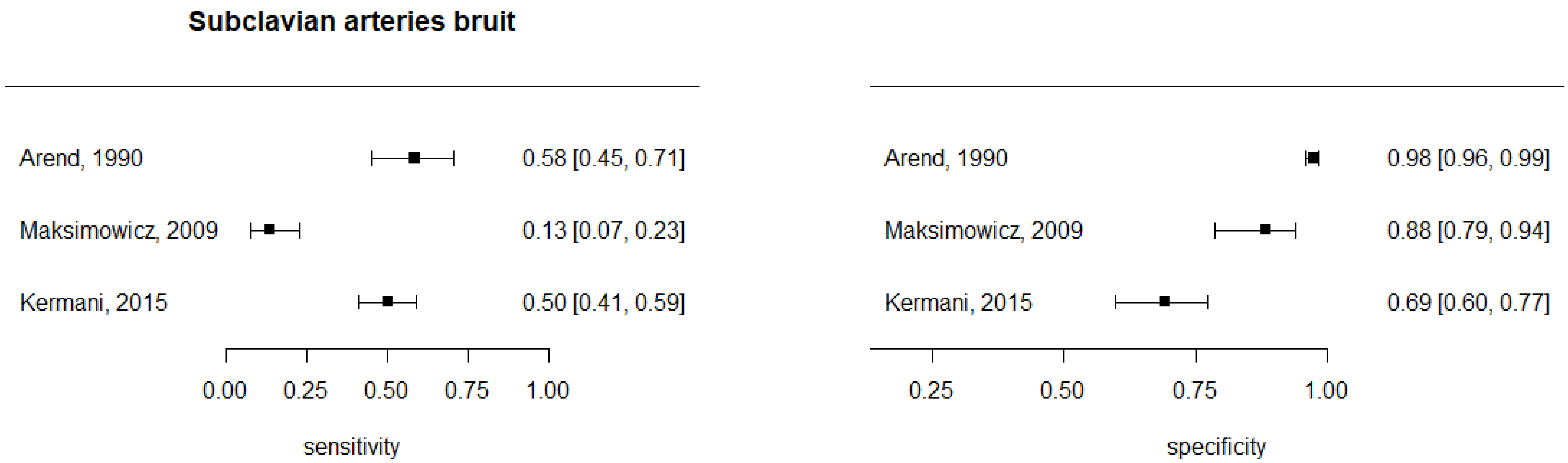

Subclavian arteries bruit
Arend, 1990
0.58 [0.45, 0.71]
Maksimowicz, 2009
0.13 [0.07, 0.23]
Kermani, 2015
0.50 [0.41, 0.59]
0.00
0.25
0.50
0.75
1.00
sensitivity
Arend, 1990
0.98 [0.96, 0.99]
Maksimowicz, 2009
0.88 [0.79, 0.94]
Kermani, 2015
0.69 [0.60, 0.77]
0.25
0.50
0.75
1.00
specificity

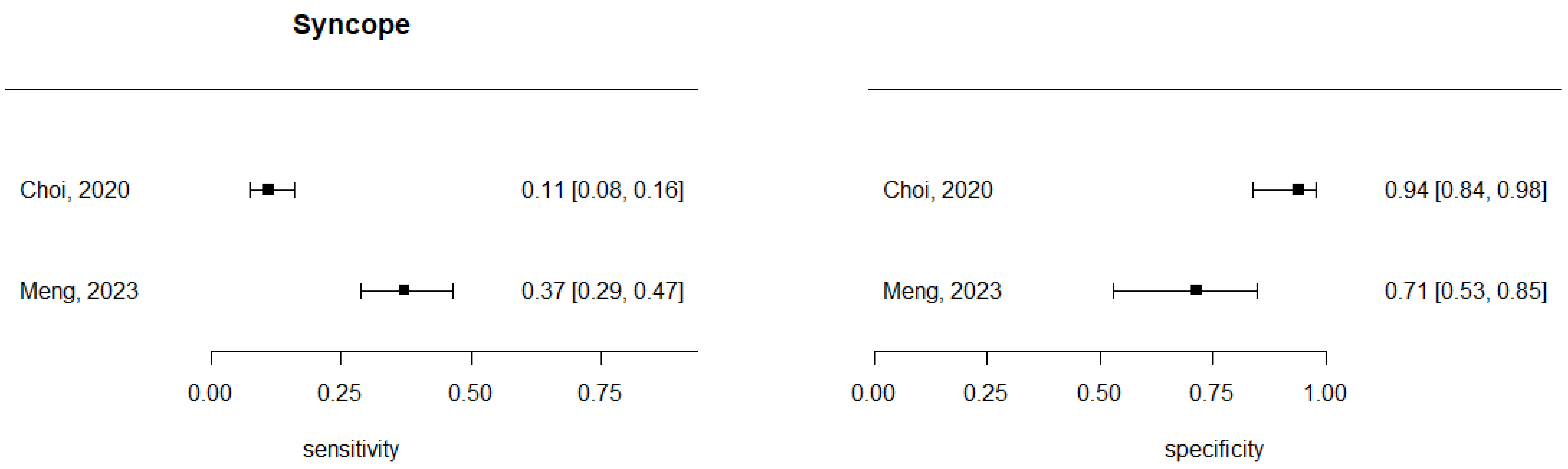
Syncope
Choi, 2020
0.11 [0.08, 0.16]
Meng, 2023
0.37 [0.29, 0.47]
0.00
0.25
0.50
0.75
sensitivity
Choi, 2020
0.94 [0.84, 0.98]
Meng, 2023
0.71 [0.53, 0.85]
0.00
0.25
0.50
0.75
1.00
specificity

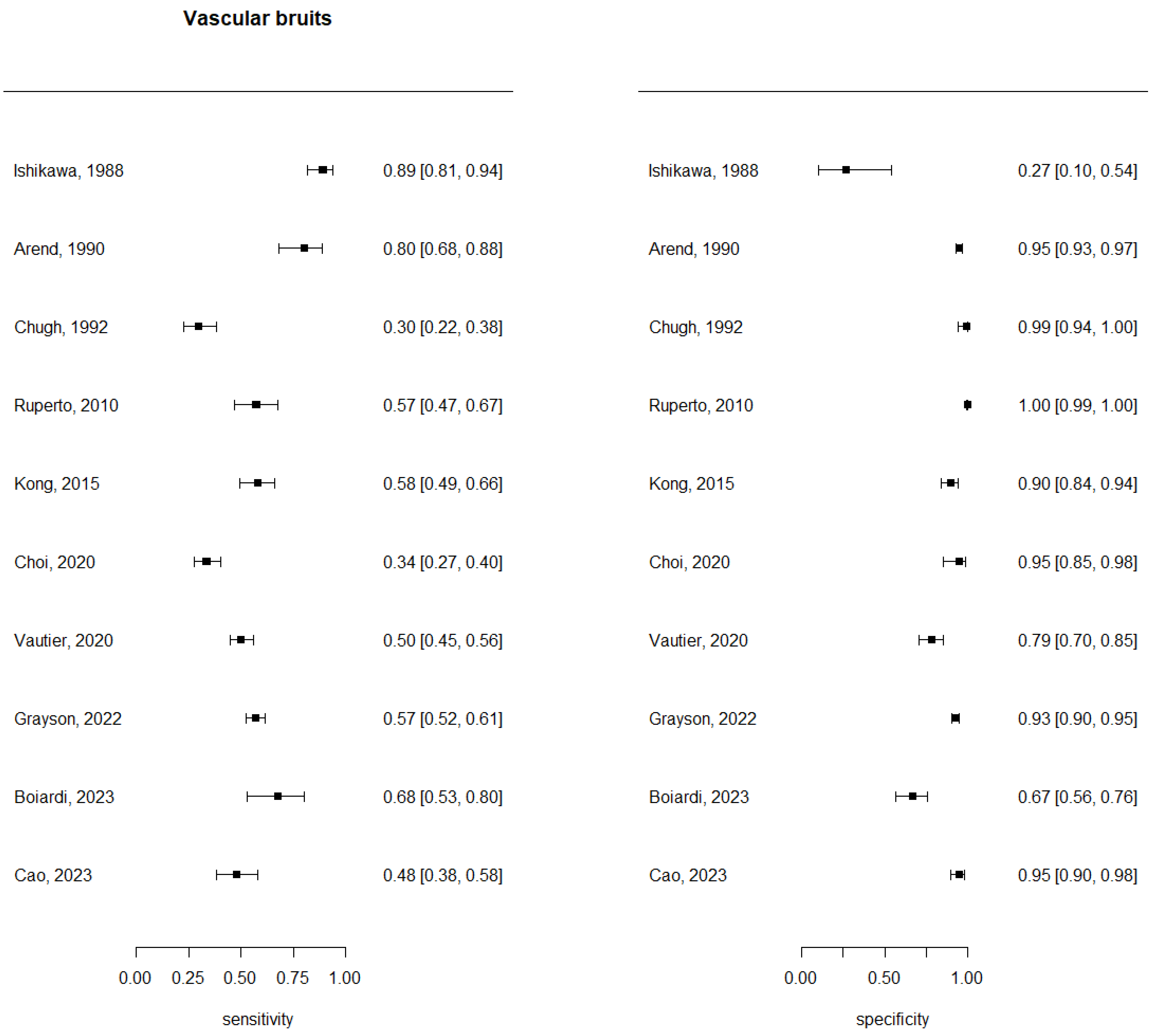
Vascular bruits
Ishikawa, 1988
0.89 [0.81, 0.94]
Arend, 1990
0.80 [0.68, 0.88]
Chugh, 1992
0.30 [0.22, 0.38]
Ruperto, 2010
0.57 [0.47, 0.67]
Kong, 2015
0.58 [0.49, 0.66]
Choi, 2020
0.34 [0.27, 0.40]
Vautier, 2020
0.50 [0.45, 0.56]
Grayson, 2022
0.57 [0.52, 0.61]
Boiardi, 2023
0.68 [0.53, 0.80]
Cao, 2023
0.48 [0.38, 0.58]
0.00
0.25
0.50
0.75
1.00
sensitivity
Ishikawa, 1988
0.27 [0.10, 0.54]
Arend, 1990
0.95 [0.93, 0.97]
Chugh, 1992
0.99 [0.94, 1.00]
Ruperto, 2010
1.00 [0.99, 1.00]
Kong, 2015
0.90 [0.84, 0.94]
Choi, 2020
0.95 [0.85, 0.98]
Vautier, 2020
0.79 [0.70, 0.85]
Grayson, 2022
0.93 [0.90, 0.95]
Boiardi, 2023
0.67 [0.56, 0.76]
Cao, 2023
0.95 [0.90, 0.98]
0.00
0.50
1.00
specificity

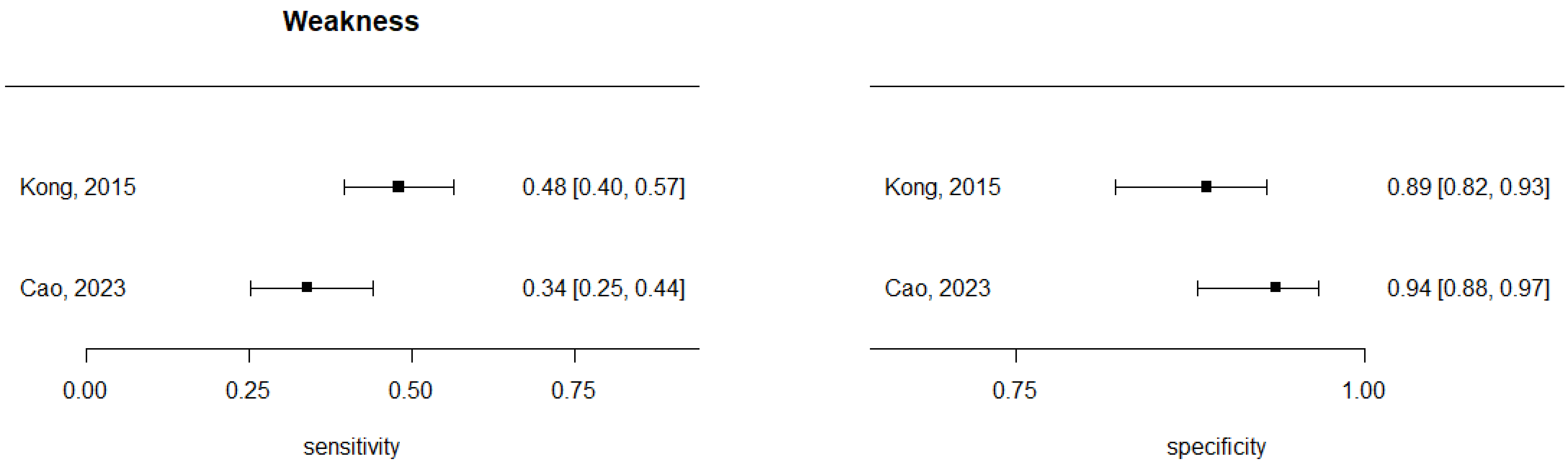
Weakness
Kong, 2015
0.48 [0.40, 0.57]
Cao, 2023
0.34 [0.25, 0.44]
0.00
0.25
0.50
0.75
sensitivity
Kong, 2015
0.89 [0.82, 0.93]
Cao, 2023
0.94 [0.88, 0.97]
0.75
1.00
specificity

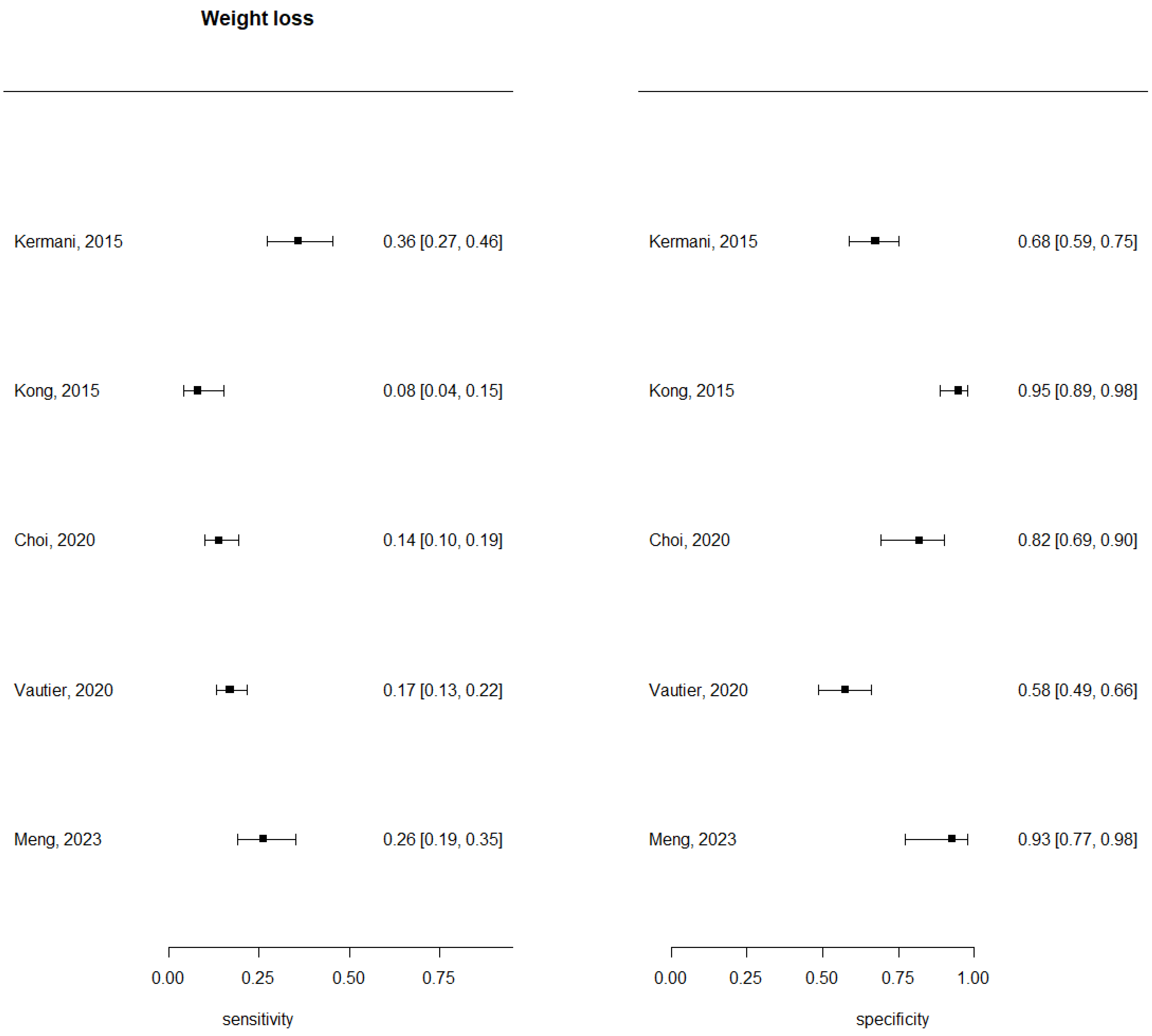
Weight loss
Kermani, 2015
0.36 [0.27, 0.46]
Kong, 2015
0.08 [0.04, 0.15]
Choi, 2020
0.14 [0.10, 0.19]
Vautier, 2020
0.17 [0.13, 0.22]
Meng, 2023
0.26 [0.19, 0.35]
0.00
0.25
0.50
0.75
sensitivity
Kermani, 2015
0.68 [0.59, 0.75]
Kong, 2015
0.95 [0.89, 0.98]
Choi, 2020
0.82 [0.69, 0.90]
Vautier, 2020
0.58 [0.49, 0.66]
Meng, 2023
0.93 [0.77, 0.98]
0.00
0.25
0.50
0.75
1.00
specificity
