# Supplementary material for: Diagnostic Accuracy of Clinical Findings for Takayasu Arteritis: A Rapid Review and Meta-Analysis
Source: Int J Vasc Med. 2025 Sep 9;2025:6092362. doi: 10.1155/ijvm/6092362 (PMC12440657; doi:10.1155/ijvm/6092362)
Supplement: Supporting Information 3 — Figure S2: Summary receiver operating characteristics (SROC) curve for each symptom, physical sign, demographic feature, and complications listed in alphabetical order. False positive rate is equal to 1 minus specificity. Sensitivity (Se) and specificity (Sp) of the summary estimates are indicated on each plot. CI, confidence intervals. [file 6092362.f3.pdf]

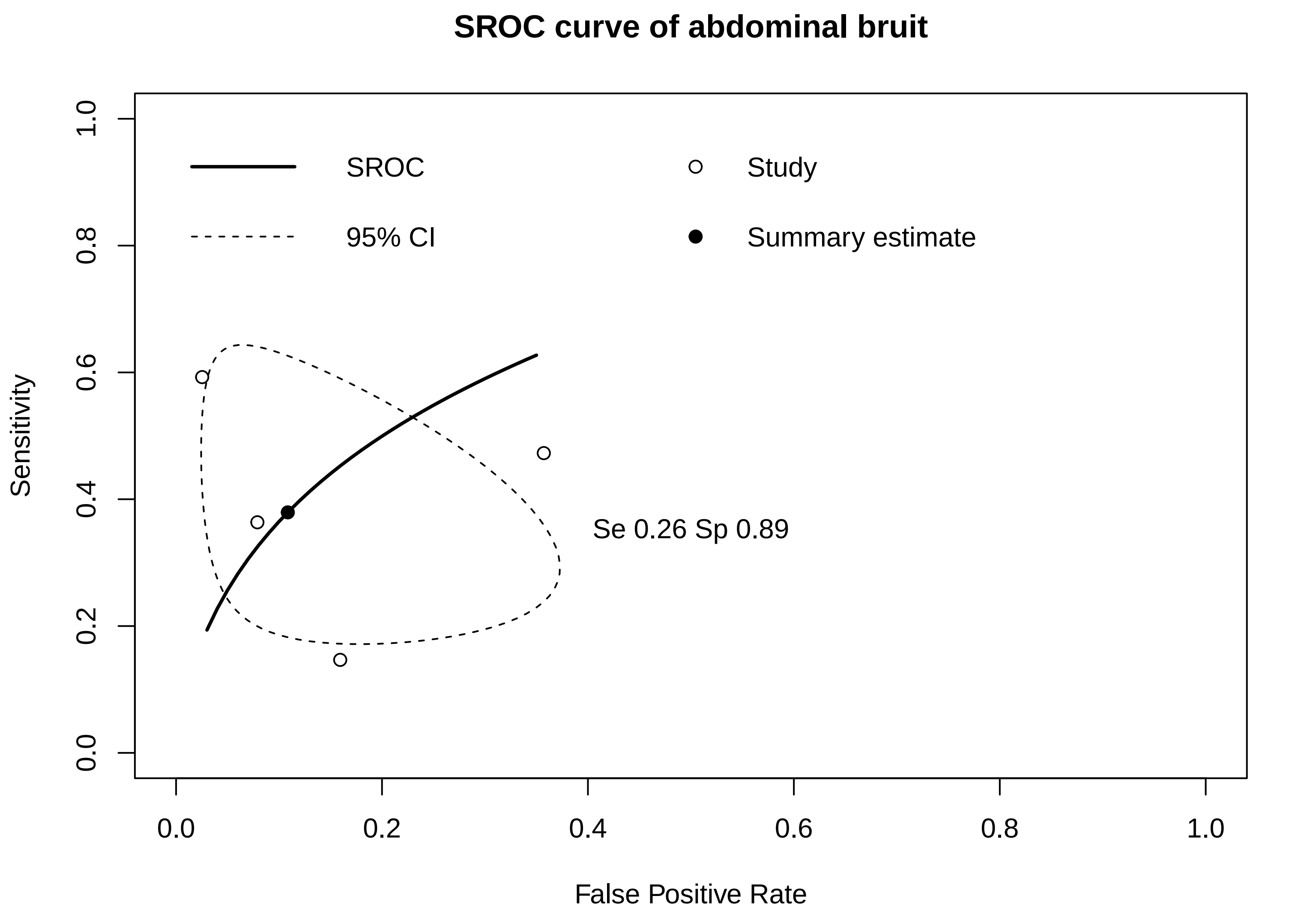

SROC curve of abdominal bruit
SROC
Study
95% CI
Summary estimate
Se 0.26 Sp 0.89
Sensitivity
False Positive Rate
0.0
0.2
0.4
0.6
0.8
1.0

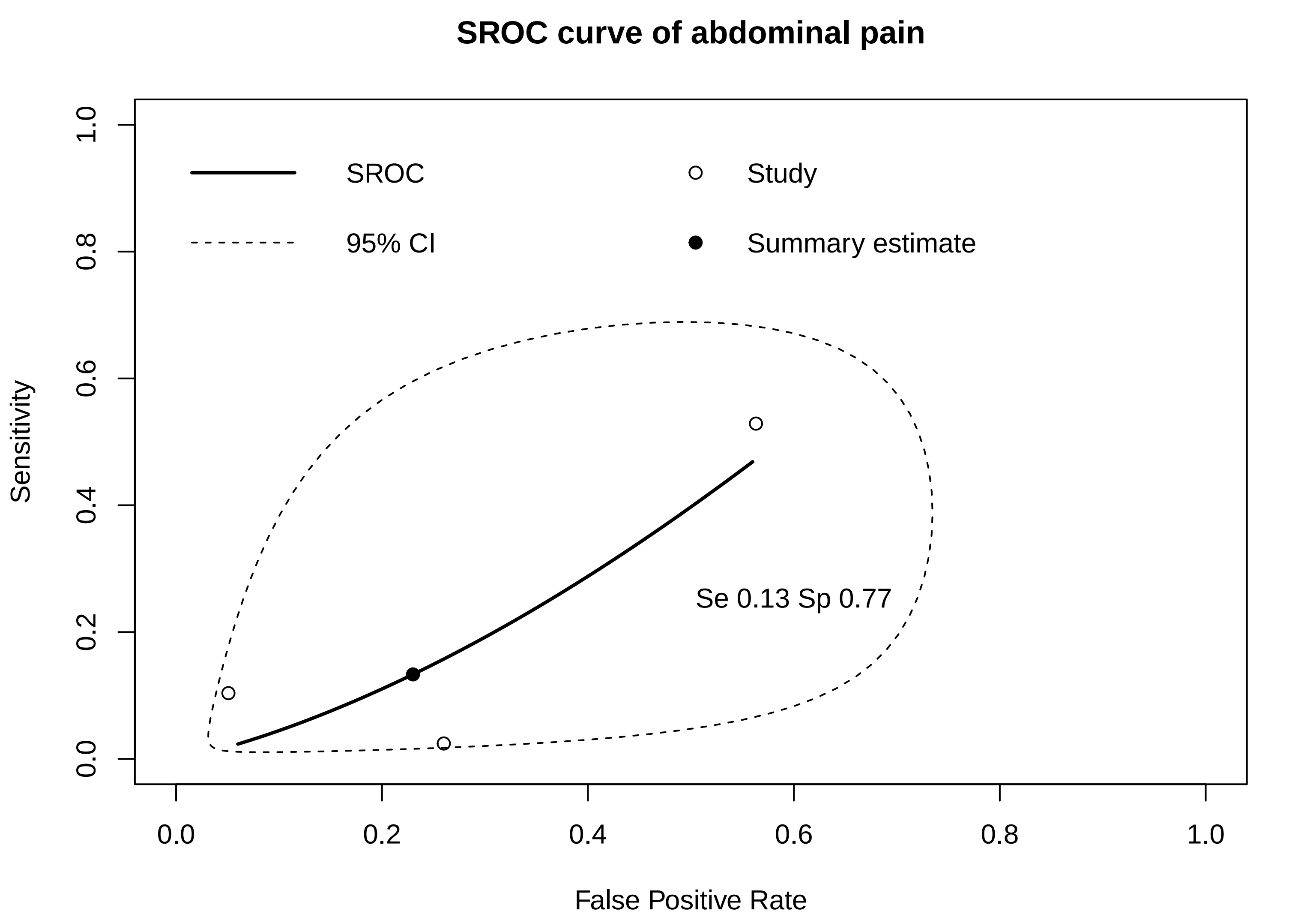
SROC curve of abdominal pain
SROC
95% CI
Study
Summary estimate
Se 0.13 Sp 0.77
Sensitivity
0.0
0.2
0.4
0.6
0.8
1.0
False Positive Rate
0.0
0.2
0.4
0.6
0.8
1.0

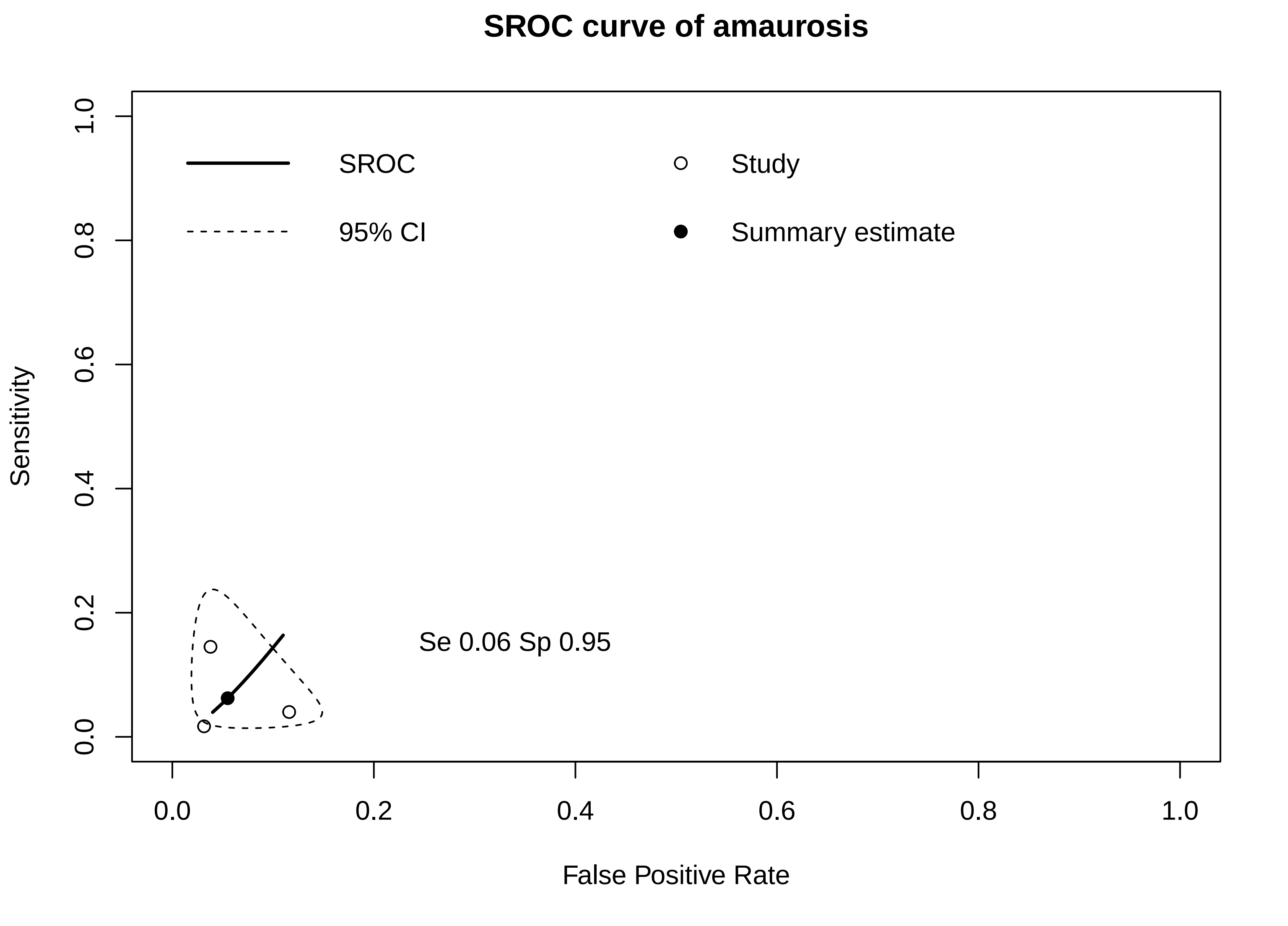

SROC curve of amaurosis
SROC
Study
95% CI
Summary estimate
Se 0.06 Sp 0.95
Sensitivity
0.0
0.2
0.4
0.6
0.8
1.0
False Positive Rate

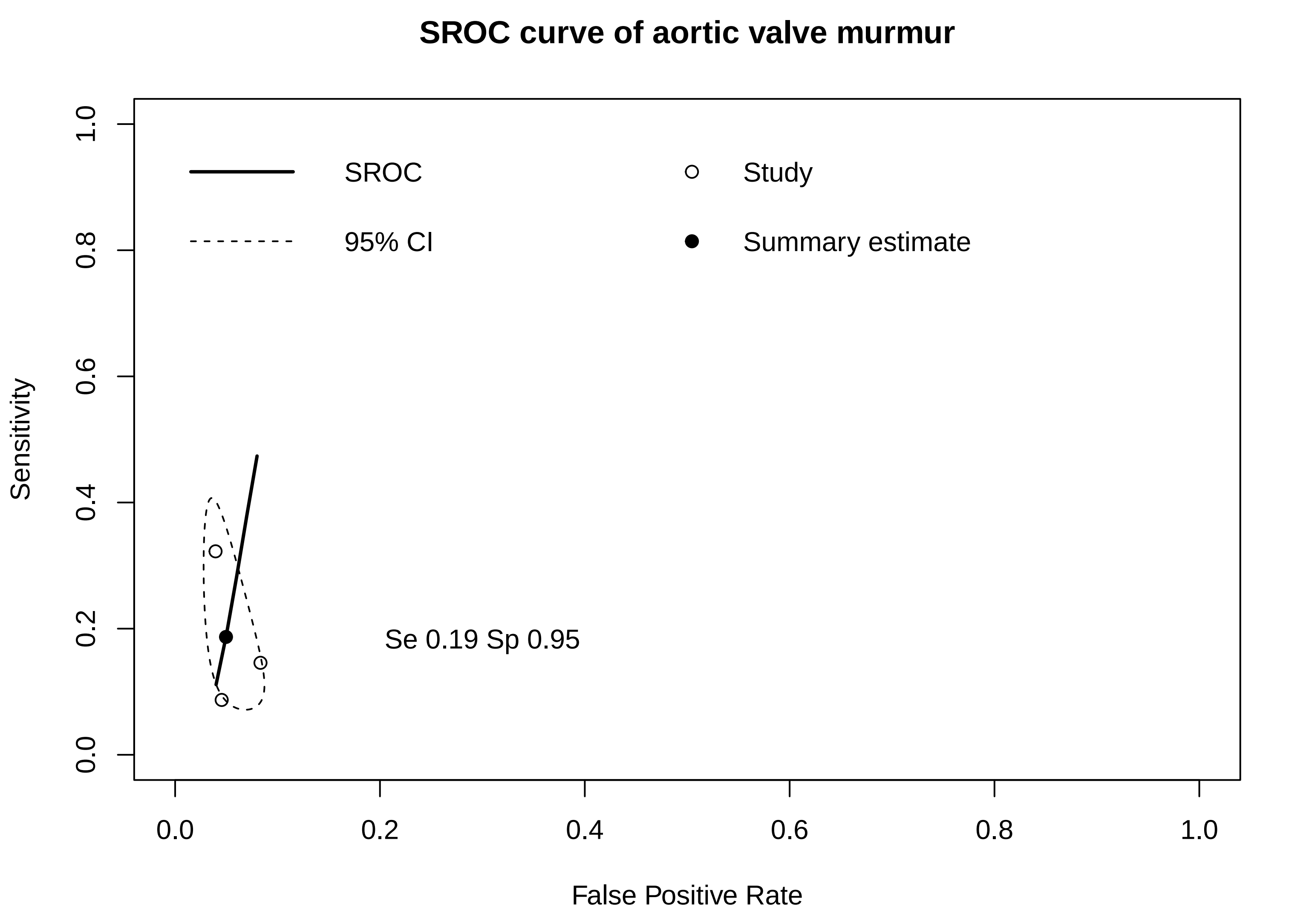

SROC curve of aortic valve murmur
SROC
95% CI
Study
Summary estimate
Se 0.19 Sp 0.95
Sensitivity
False Positive Rate
0.0
0.2
0.4
0.6
0.8
1.0

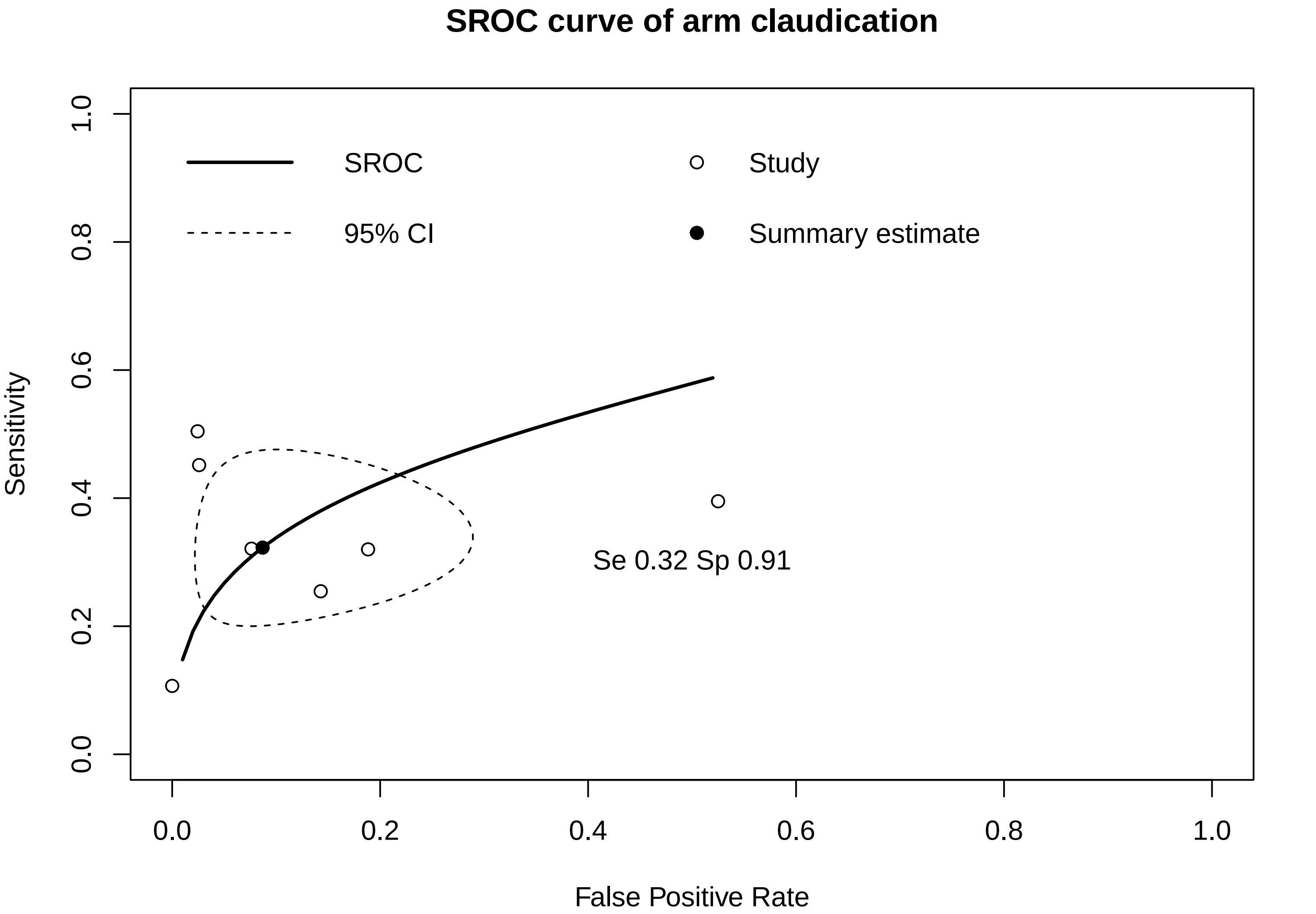
SROC curve of arm claudication
SROC
95% CI
Study
Summary estimate
Se 0.32 Sp 0.91
Sensitivity
False Positive Rate
0.0
0.2
0.4
0.6
0.8
1.0

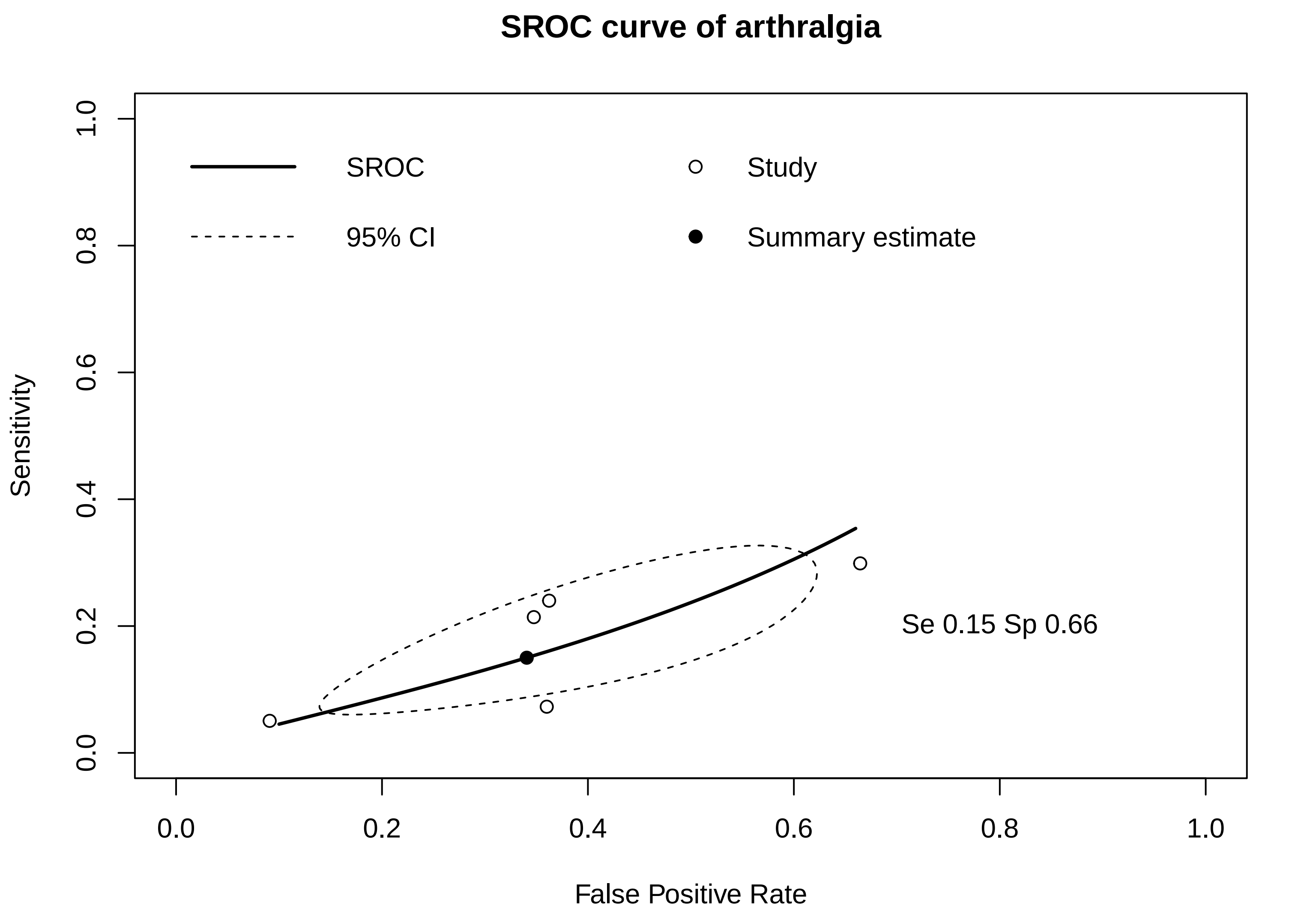
SROC curve of arthralgia
SROC
Study
95% CI
Summary estimate
Se 0.15 Sp 0.66
Sensitivity
0.0
0.2
0.4
0.6
0.8
1.0
False Positive Rate

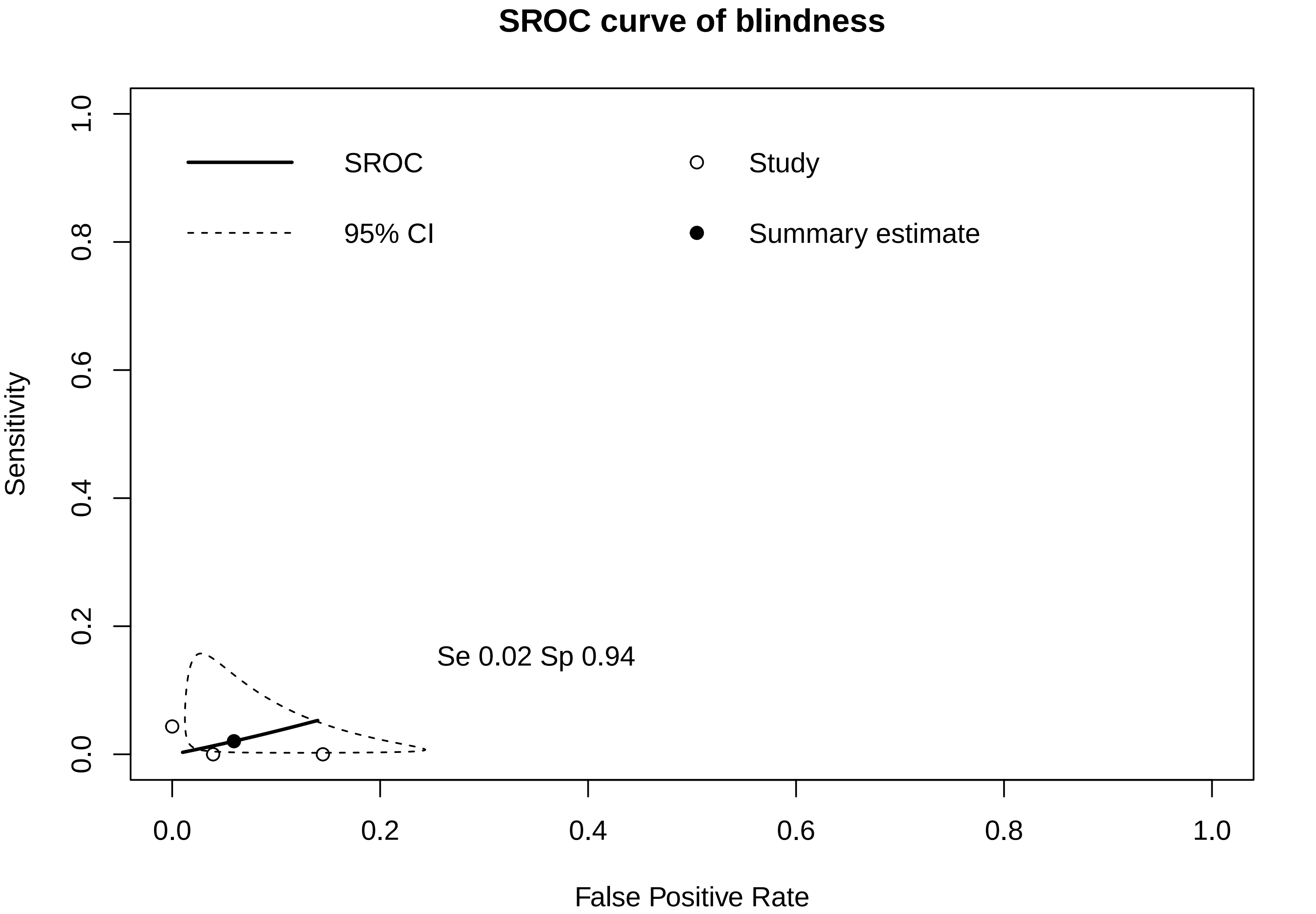

SROC curve of blindness
SROC
95% CI
Study
Summary estimate
Se 0.02 Sp 0.94
Sensitivity
0.0
0.2
0.4
0.6
0.8
1.0
False Positive Rate
0.0
0.2
0.4
0.6
0.8
1.0

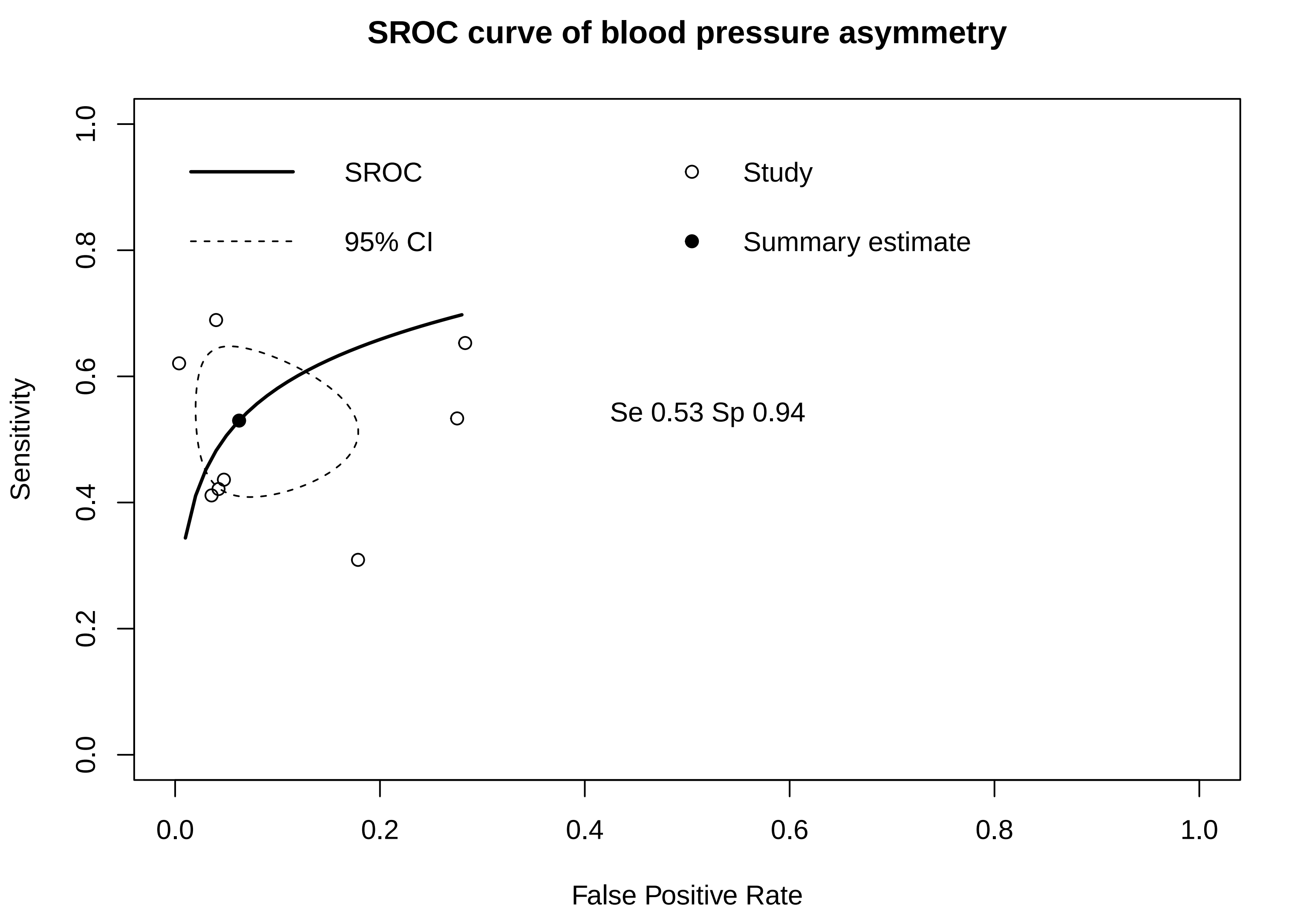
SROC curve of blood pressure asymmetry
SROC
95% CI
Study
Summary estimate
Se 0.53 Sp 0.94
Sensitivity
False Positive Rate
0.0
0.2
0.4
0.6
0.8
1.0

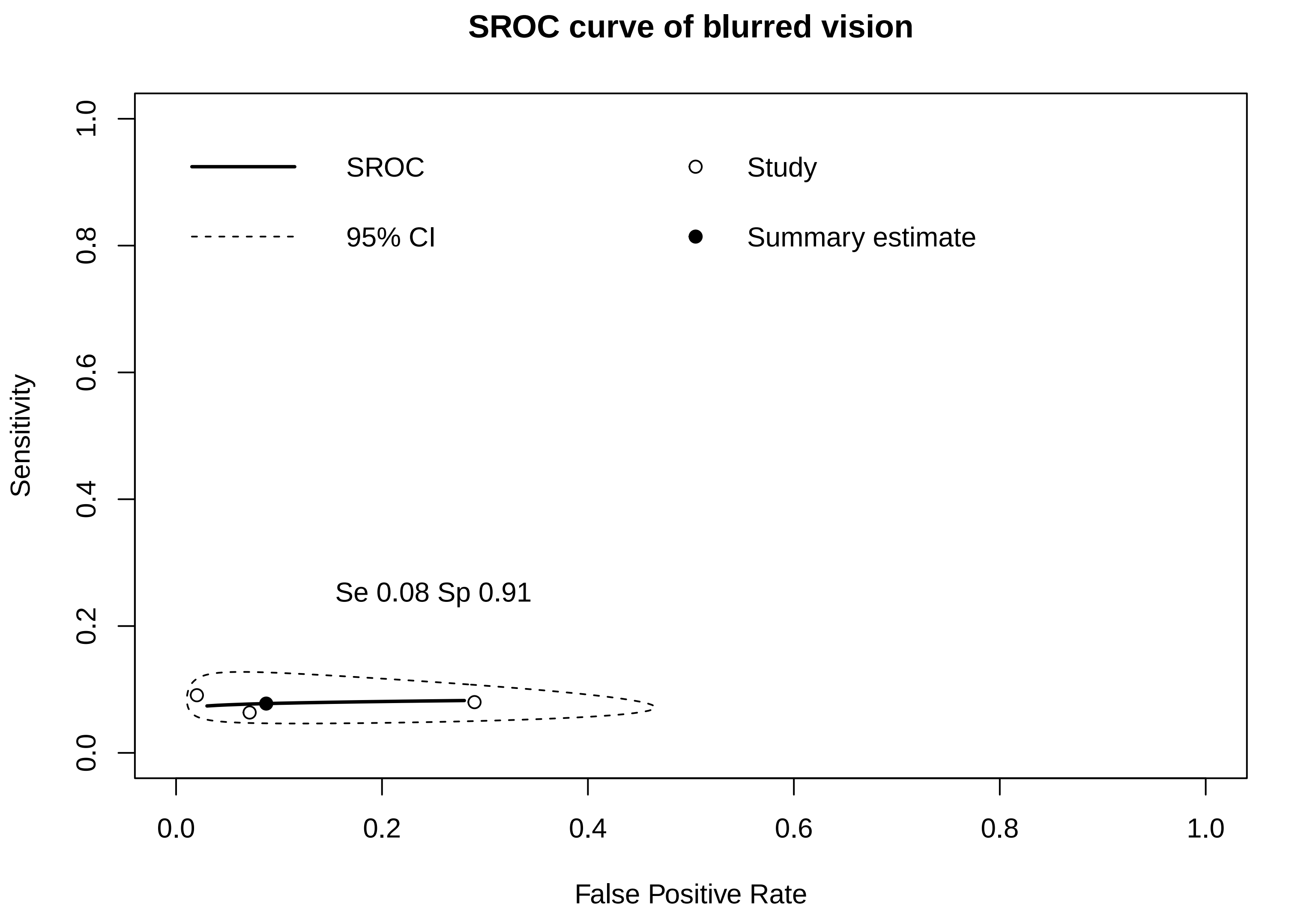
SROC curve of blurred vision
SROC
95% CI
Study
Summary estimate
Se 0.08 Sp 0.91
Sensitivity
0.0
0.2
0.4
0.6
0.8
1.0
False Positive Rate

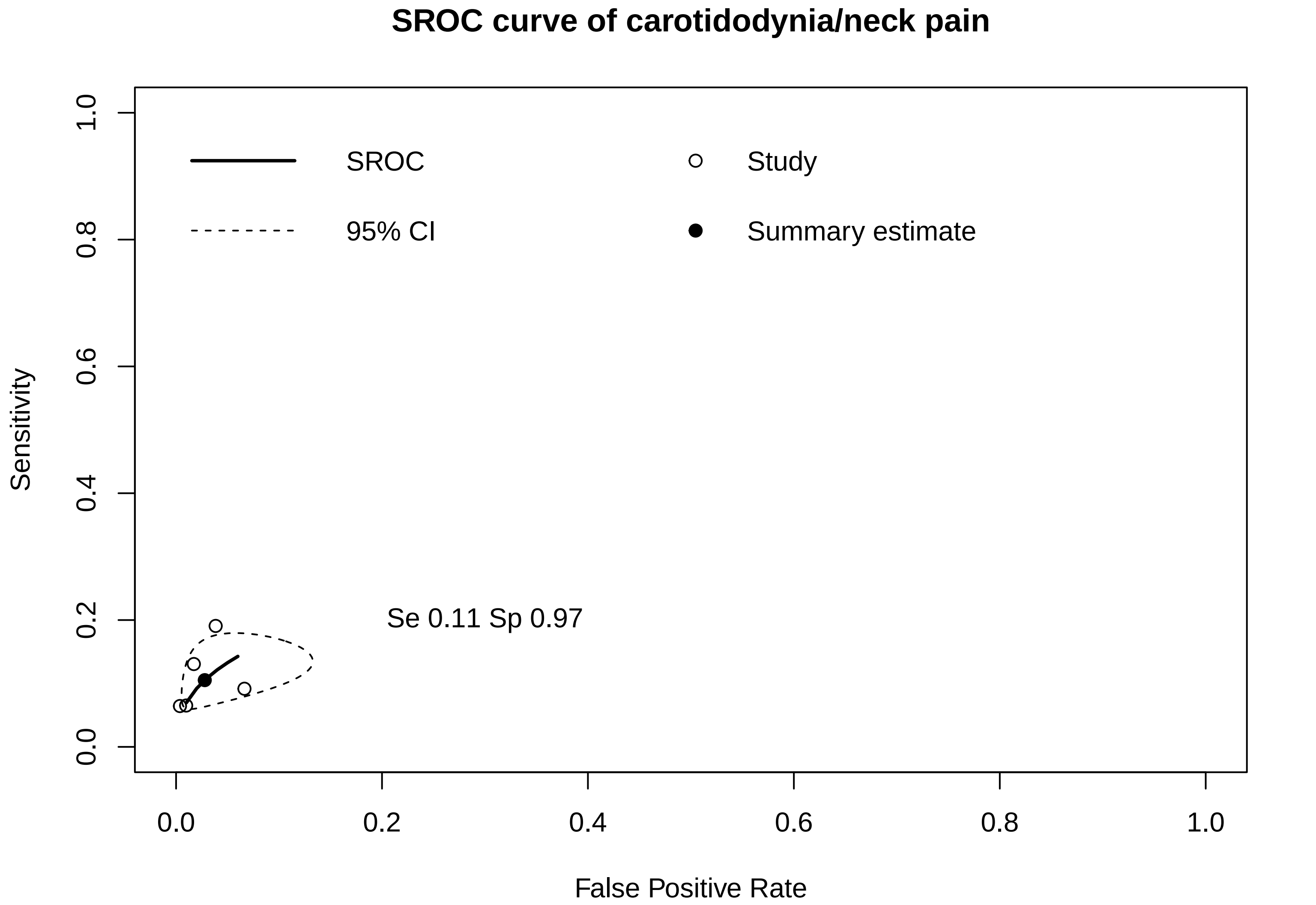

SROC curve of carotidodynia/neck pain
SROC
95% CI
Study
Summary estimate
Se 0.11 Sp 0.97
Sensitivity
0.0
0.2
0.4
0.6
0.8
1.0
False Positive Rate
0.0
0.2
0.4
0.6
0.8
1.0

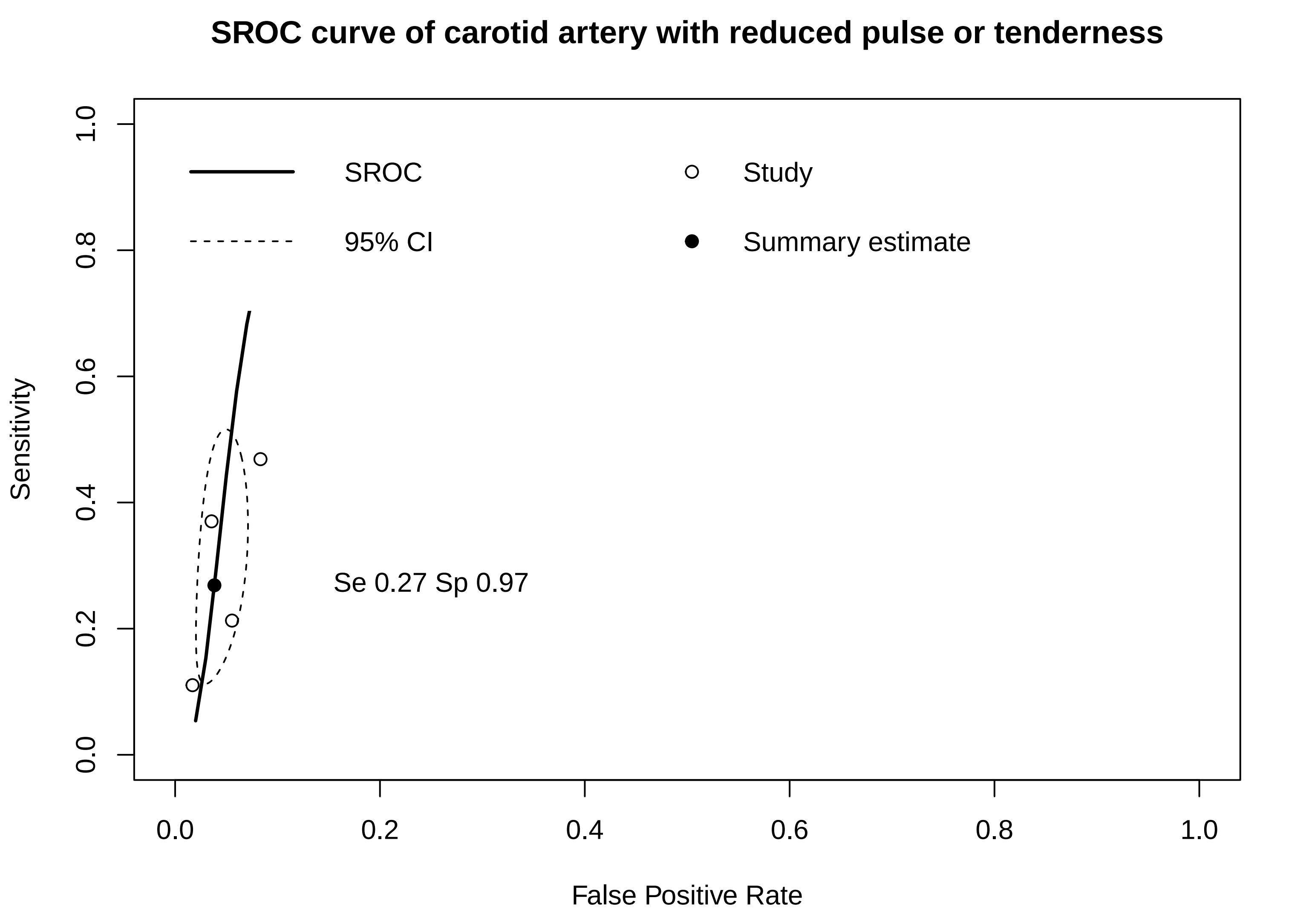

SROC curve of carotid artery with reduced pulse or tenderness
SROC
95% CI
Study
Summary estimate
Se 0.27 Sp 0.97
Sensitivity
False Positive Rate
0.0
0.2
0.4
0.6
0.8
1.0

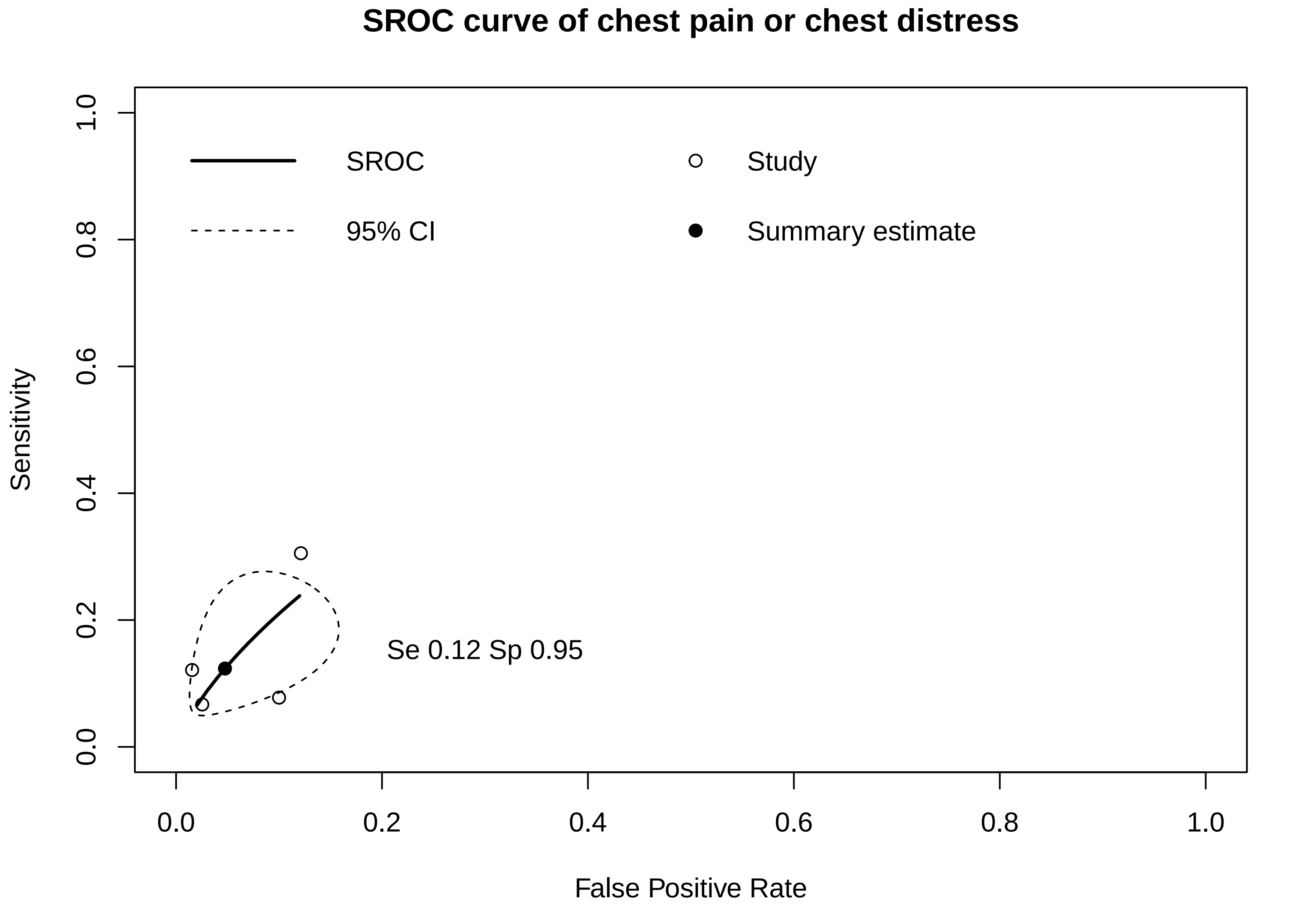
SROC curve of chest pain or chest distress
SROC
95% CI
Study
Summary estimate
Se 0.12 Sp 0.95
Sensitivity
False Positive Rate
0.0
0.2
0.4
0.6
0.8
1.0

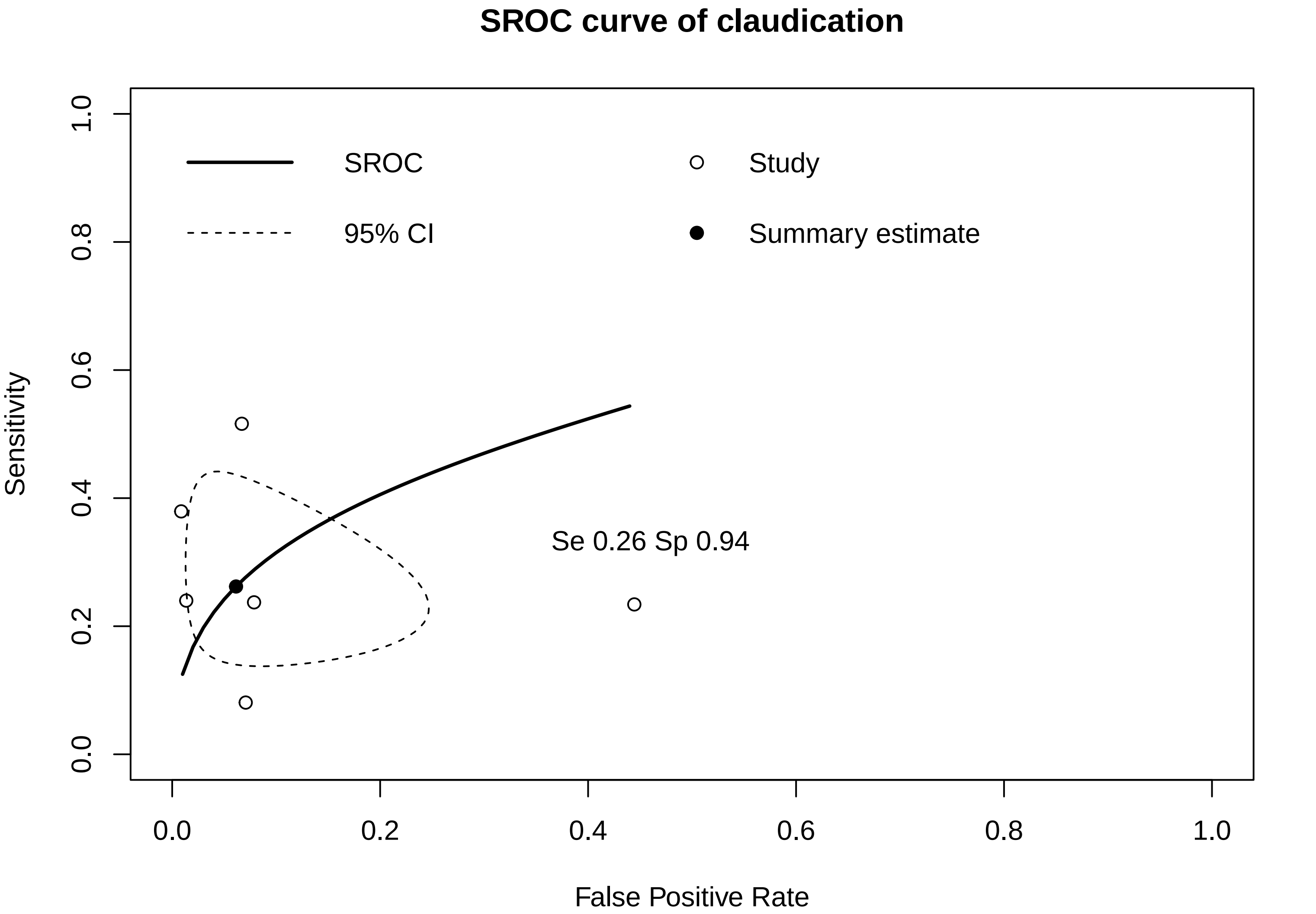
SROC curve of claudication
SROC
Study
95% CI
Summary estimate
Se 0.26 Sp 0.94
Sensitivity
False Positive Rate
0.0
0.2
0.4
0.6
0.8
1.0

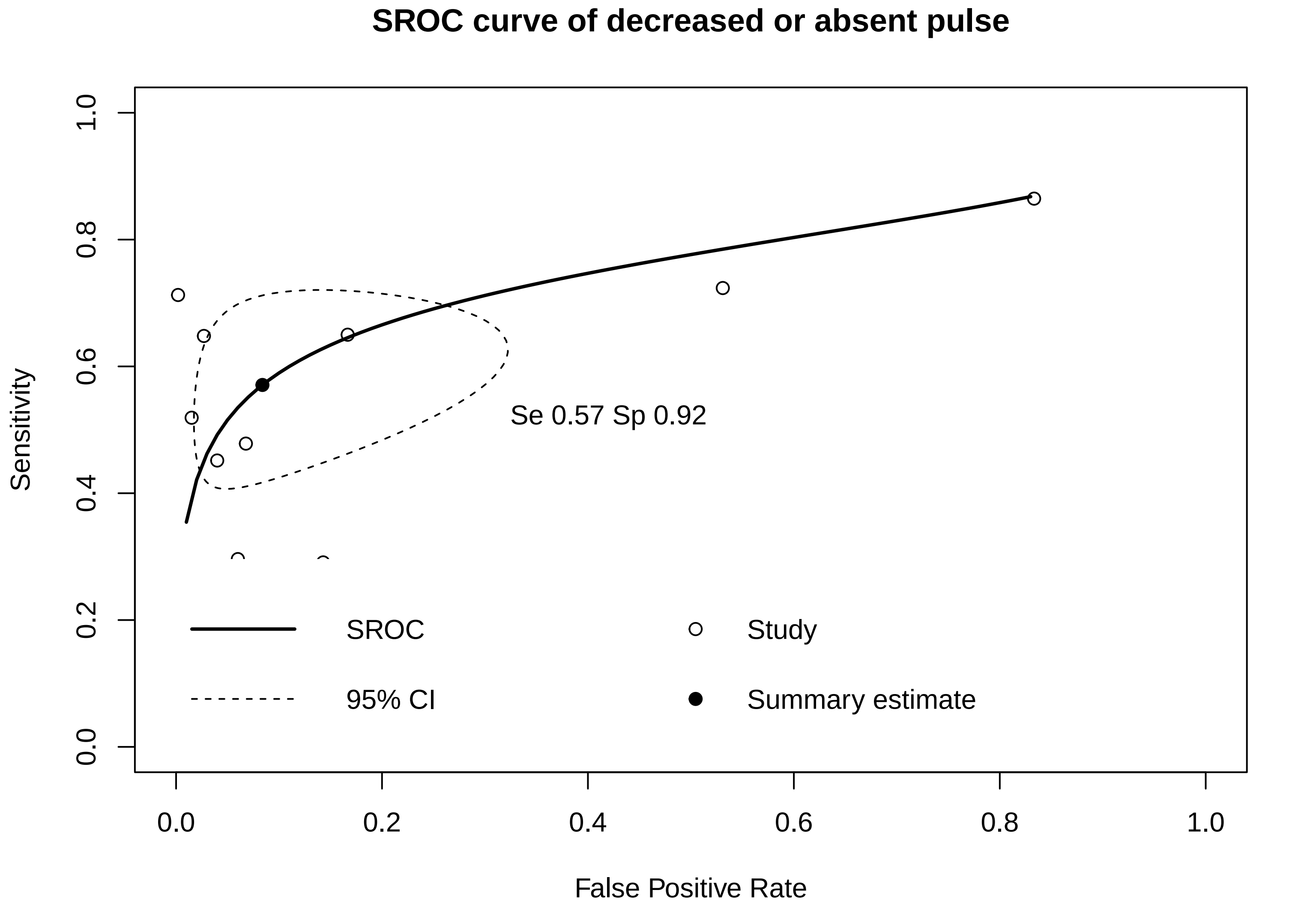
SROC curve of decreased or absent pulse
Sensitivity
False Positive Rate
0.0
0.2
0.4
0.6
0.8
1.0
Se 0.57 Sp 0.92
SROC
95% CI
Study
Summary estimate

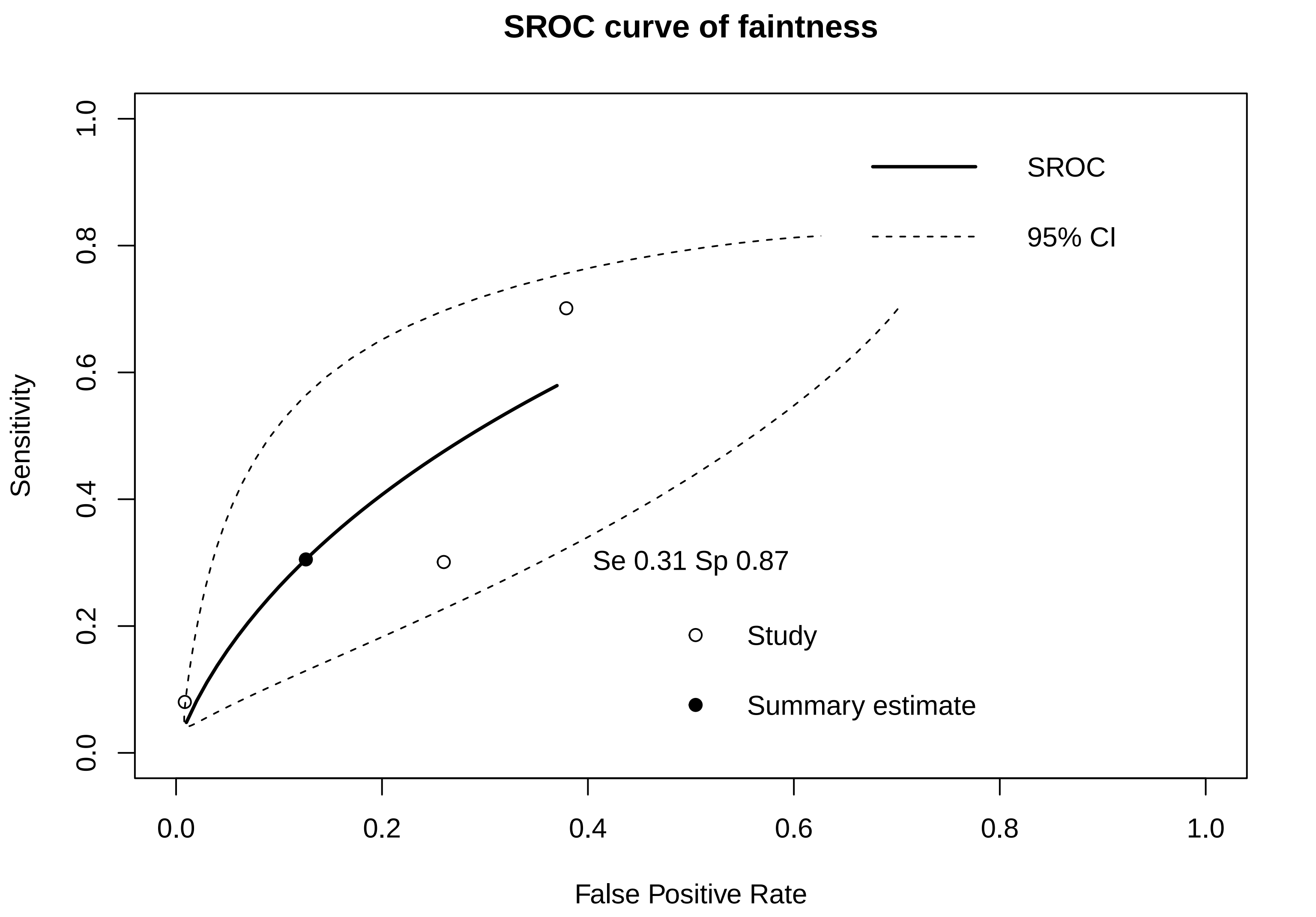
SROC curve of faintness
SROC
95% CI
Se 0.31 Sp 0.87
Study
Summary estimate
Sensitivity
0.0
0.2
0.4
0.6
0.8
1.0
False Positive Rate
0.0
0.2
0.4
0.6
0.8
1.0

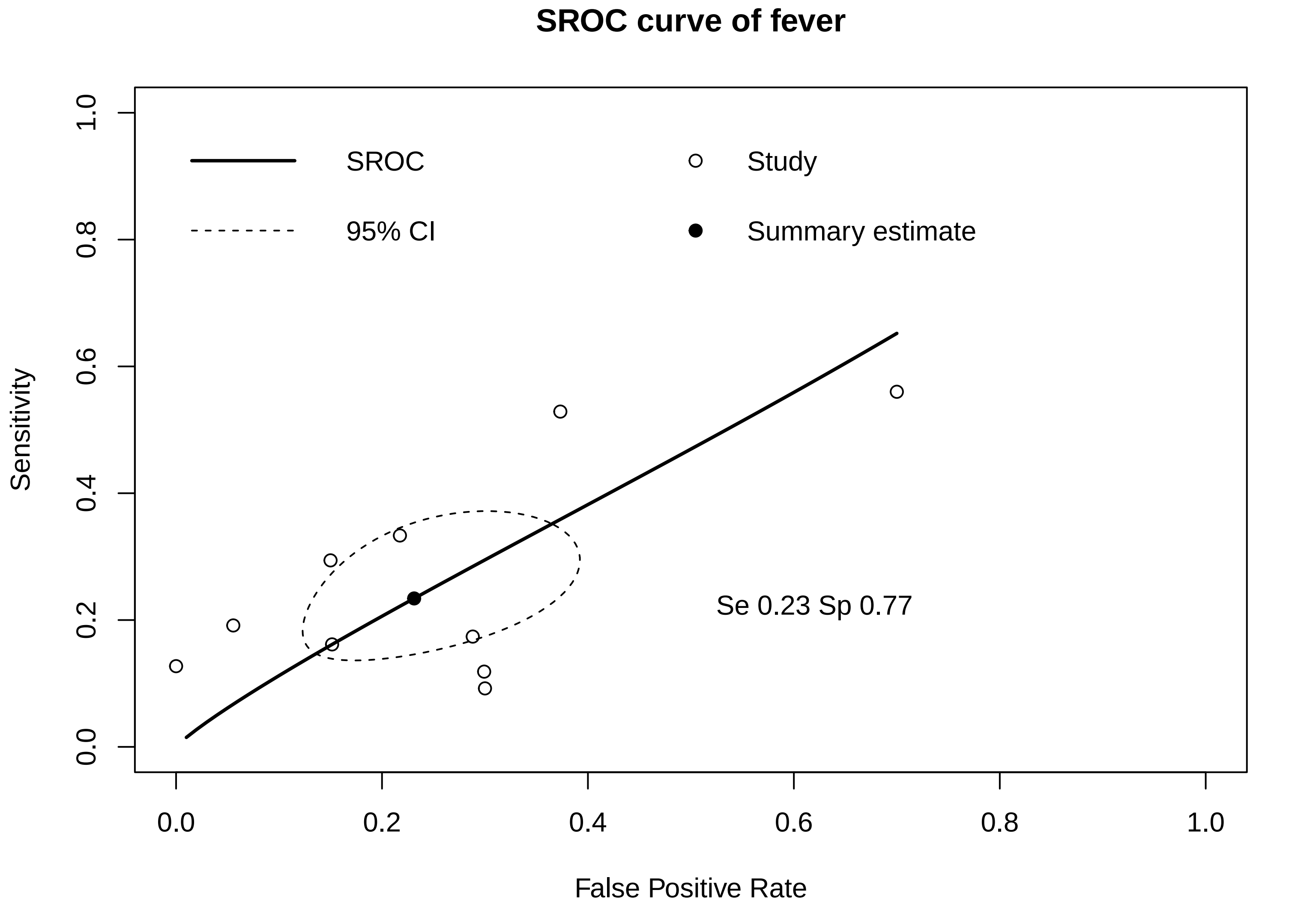

SROC curve of fever
SROC
95% CI
Study
Summary estimate
Se 0.23 Sp 0.77
Sensitivity
False Positive Rate
0.0
0.2
0.4
0.6
0.8
1.0

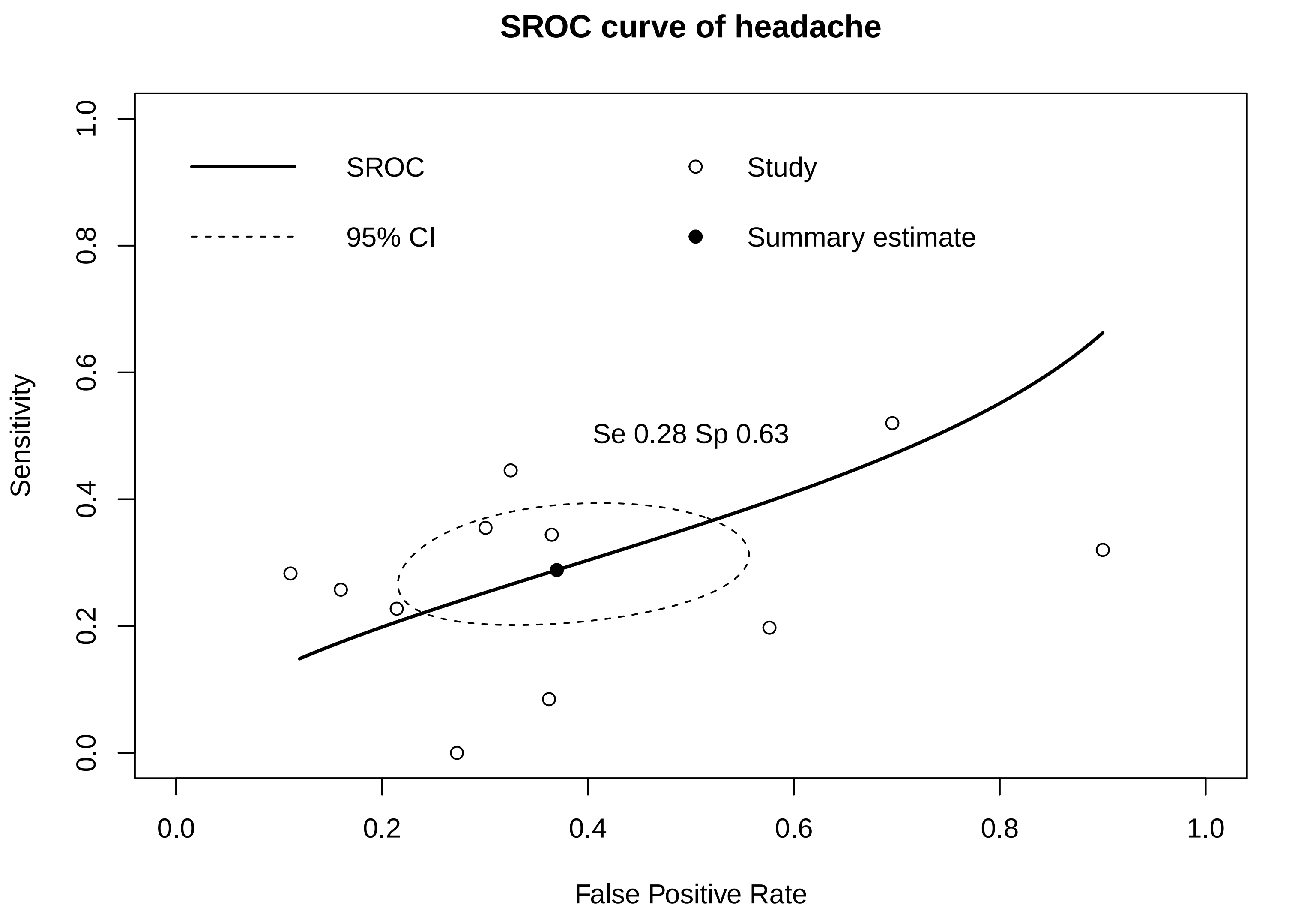
SROC curve of headache
SROC
Study
95% CI
Summary estimate
Se 0.28 Sp 0.63
Sensitivity
False Positive Rate
0.0
0.2
0.4
0.6
0.8
1.0

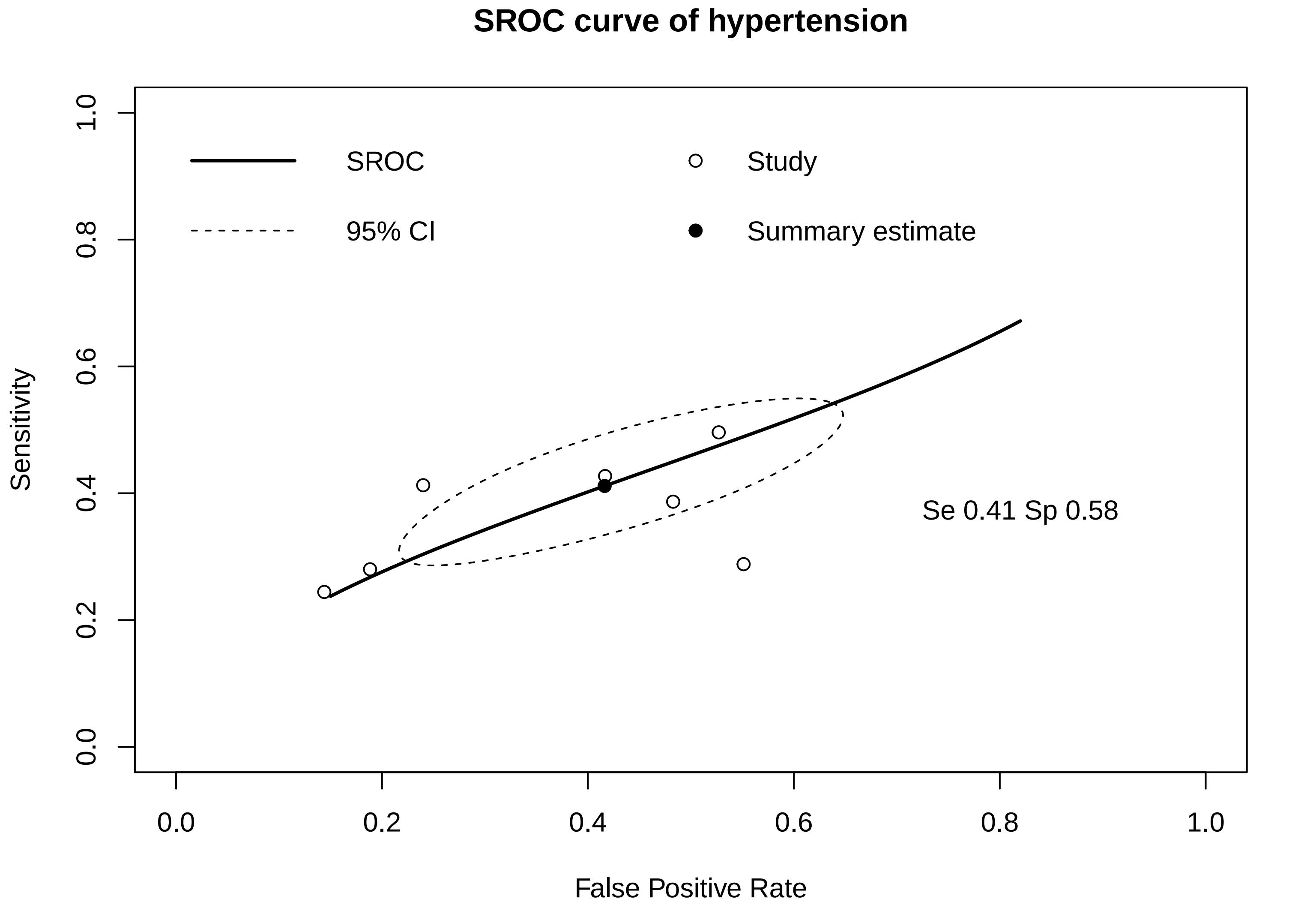

SROC curve of hypertension
SROC
Study
95% CI
Summary estimate
Se 0.41 Sp 0.58
Sensitivity
False Positive Rate
0.0
0.2
0.4
0.6
0.8
1.0

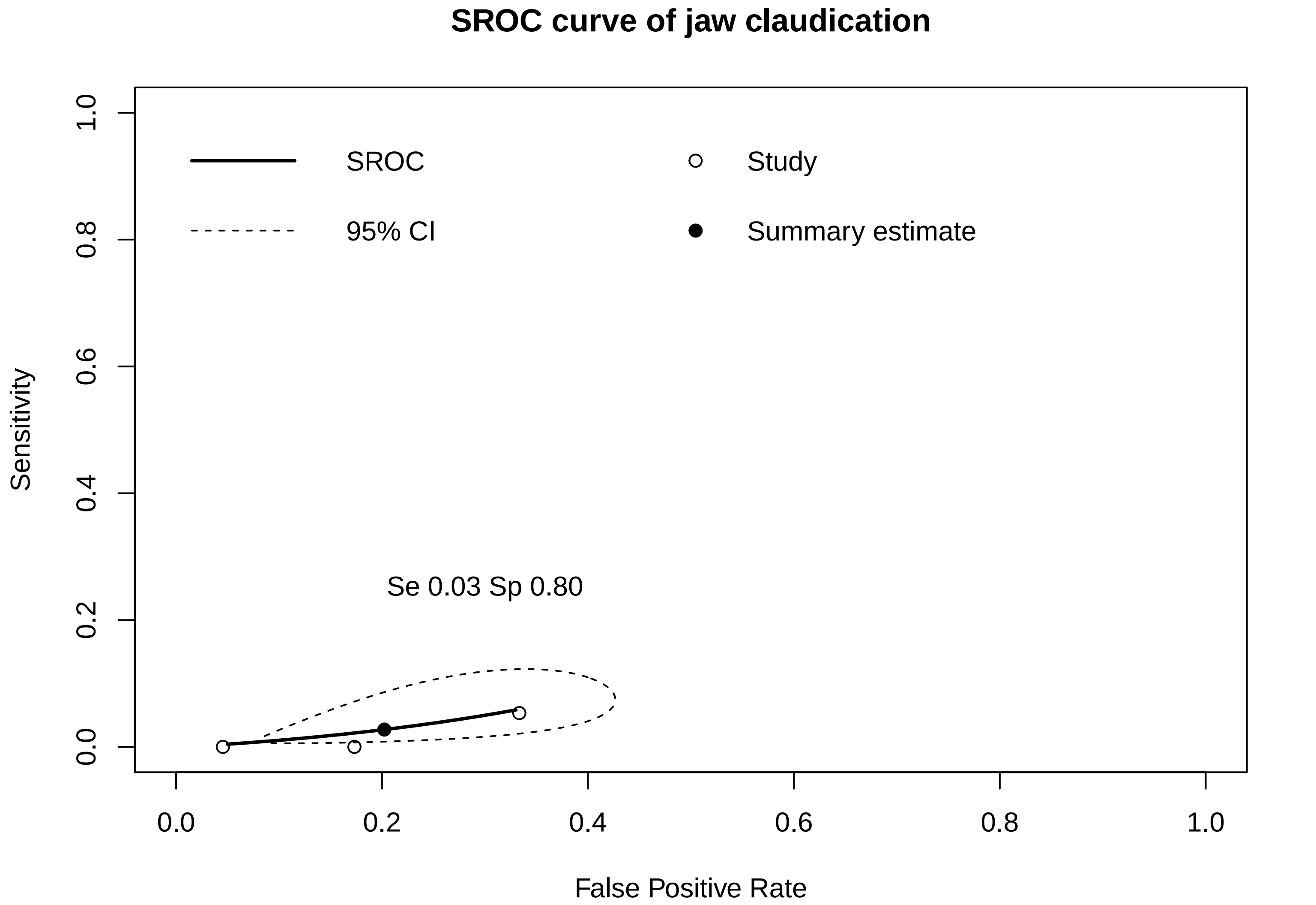
SROC curve of jaw claudication
SROC
95% CI
Study
Summary estimate
Se 0.03 Sp 0.80
Sensitivity
False Positive Rate
0.0
0.2
0.4
0.6
0.8
1.0

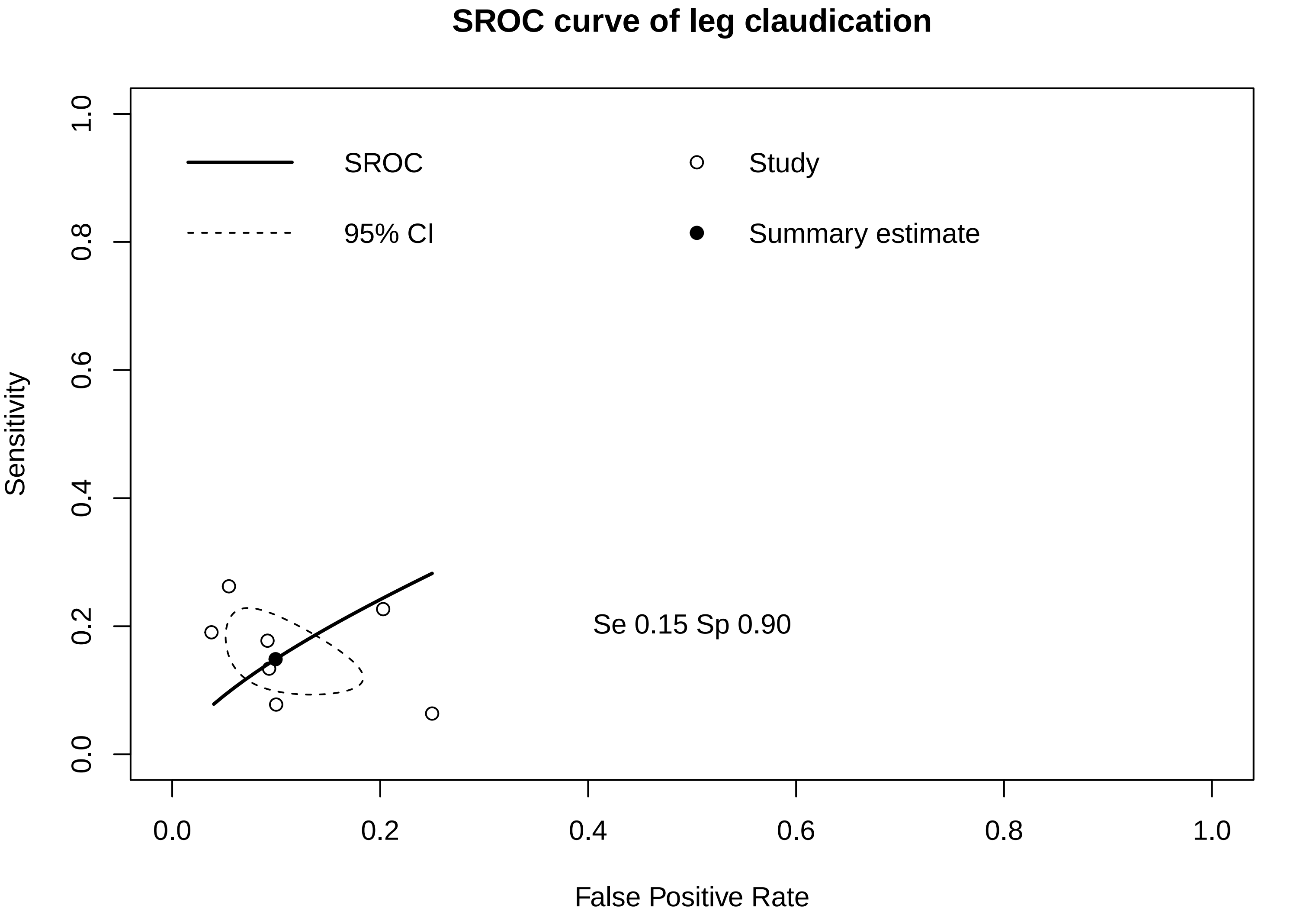
SROC curve of leg claudication
Sensitivity
False Positive Rate
0.0
0.2
0.4
0.6
0.8
1.0
SROC
95% CI
Study
Summary estimate
Se 0.15 Sp 0.90

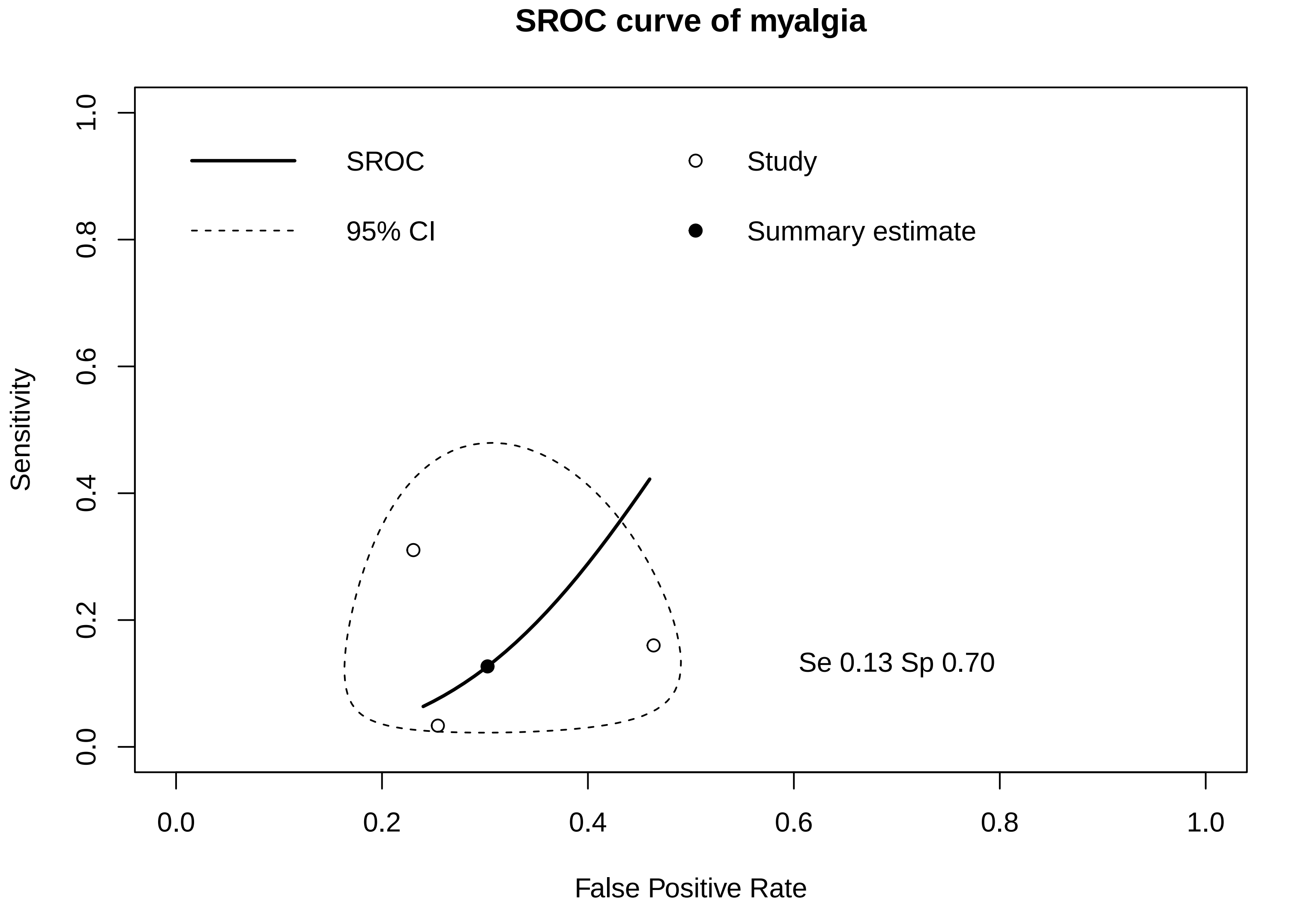

SROC curve of myalgia
SROC
Study
95% CI
Summary estimate
Se 0.13 Sp 0.70
Sensitivity
0.0
0.2
0.4
0.6
0.8
1.0
False Positive Rate
0.0
0.2
0.4
0.6
0.8
1.0

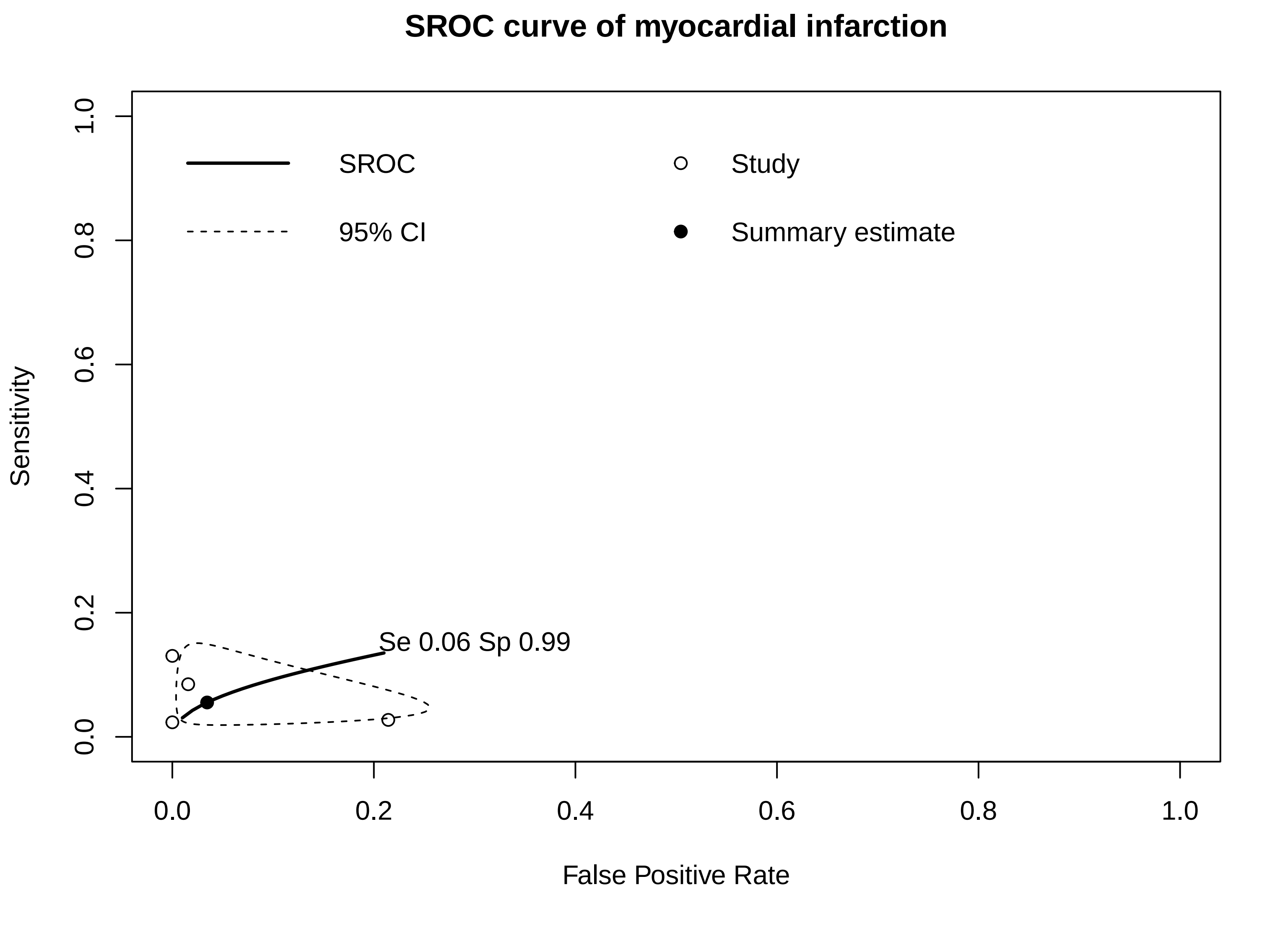
SROC curve of myocardial infarction
SROC
95% CI
Study
Summary estimate
Se 0.06 Sp 0.99
Sensitivity
False Positive Rate
0.0
0.2
0.4
0.6
0.8
1.0

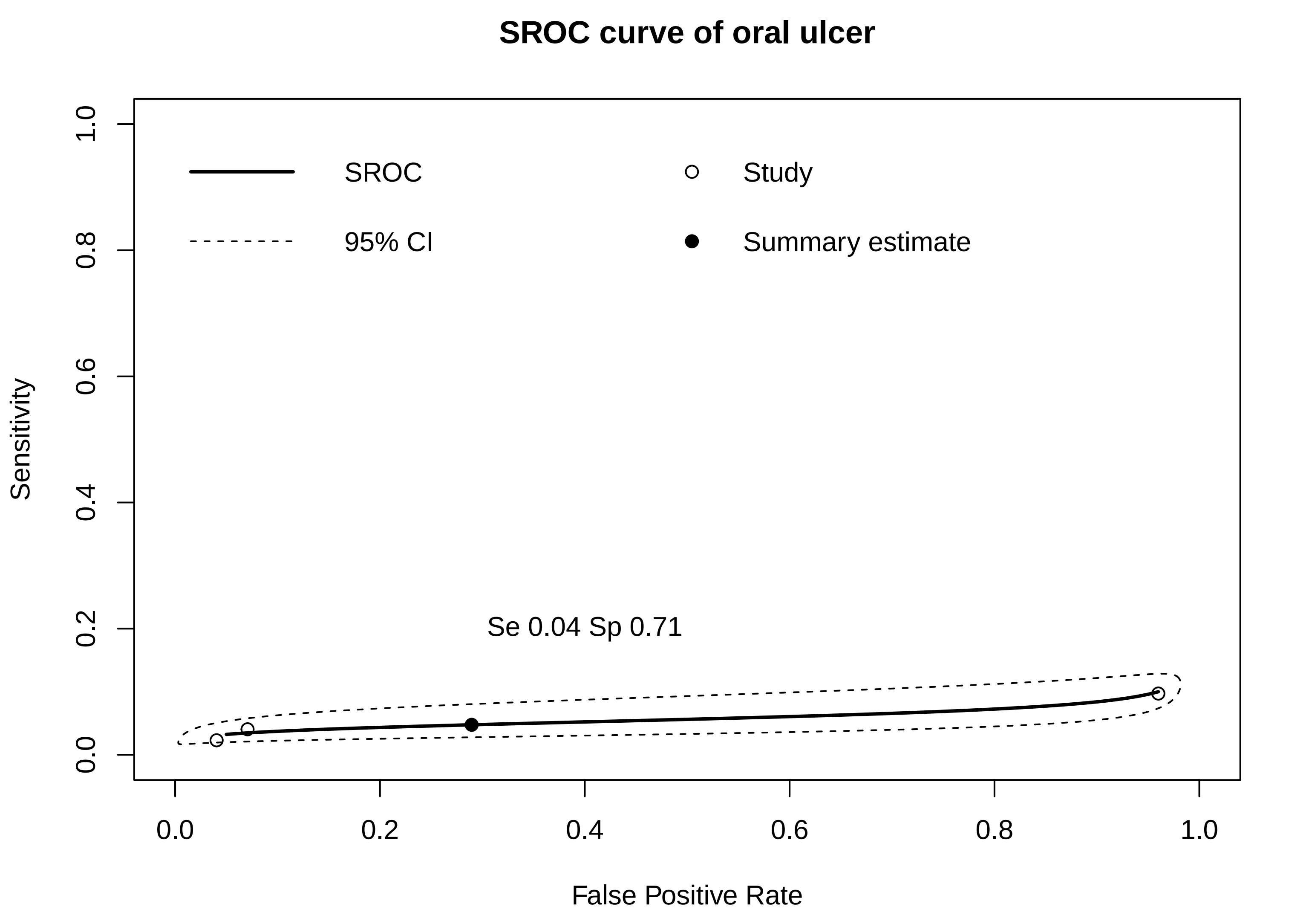

SROC curve of oral ulcer
SROC
Study
95% CI
Summary estimate
Se 0.04 Sp 0.71
Sensitivity
0.0
0.2
0.4
0.6
0.8
1.0
False Positive Rate

SROC curve of pulse deficit in arm

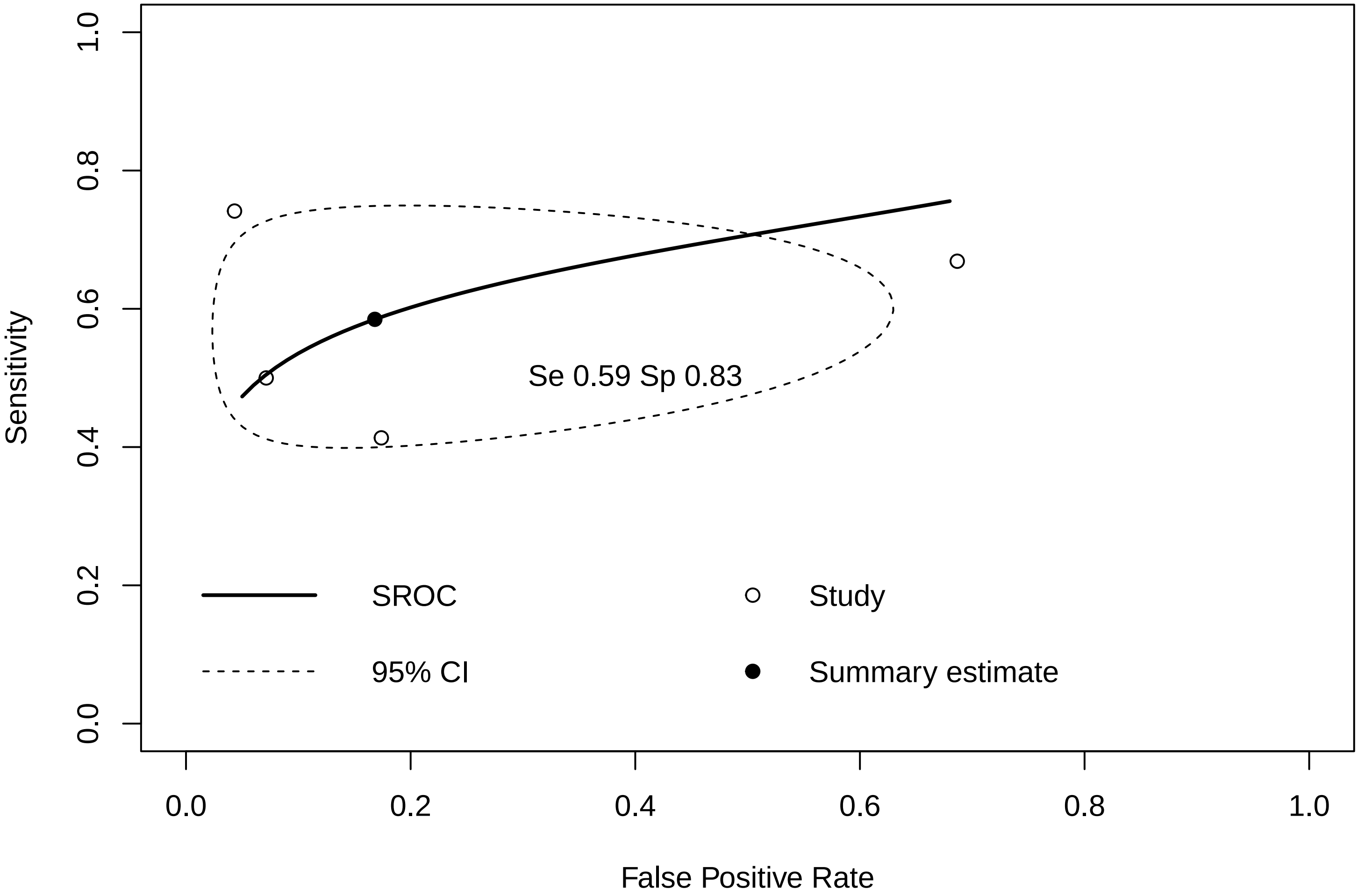

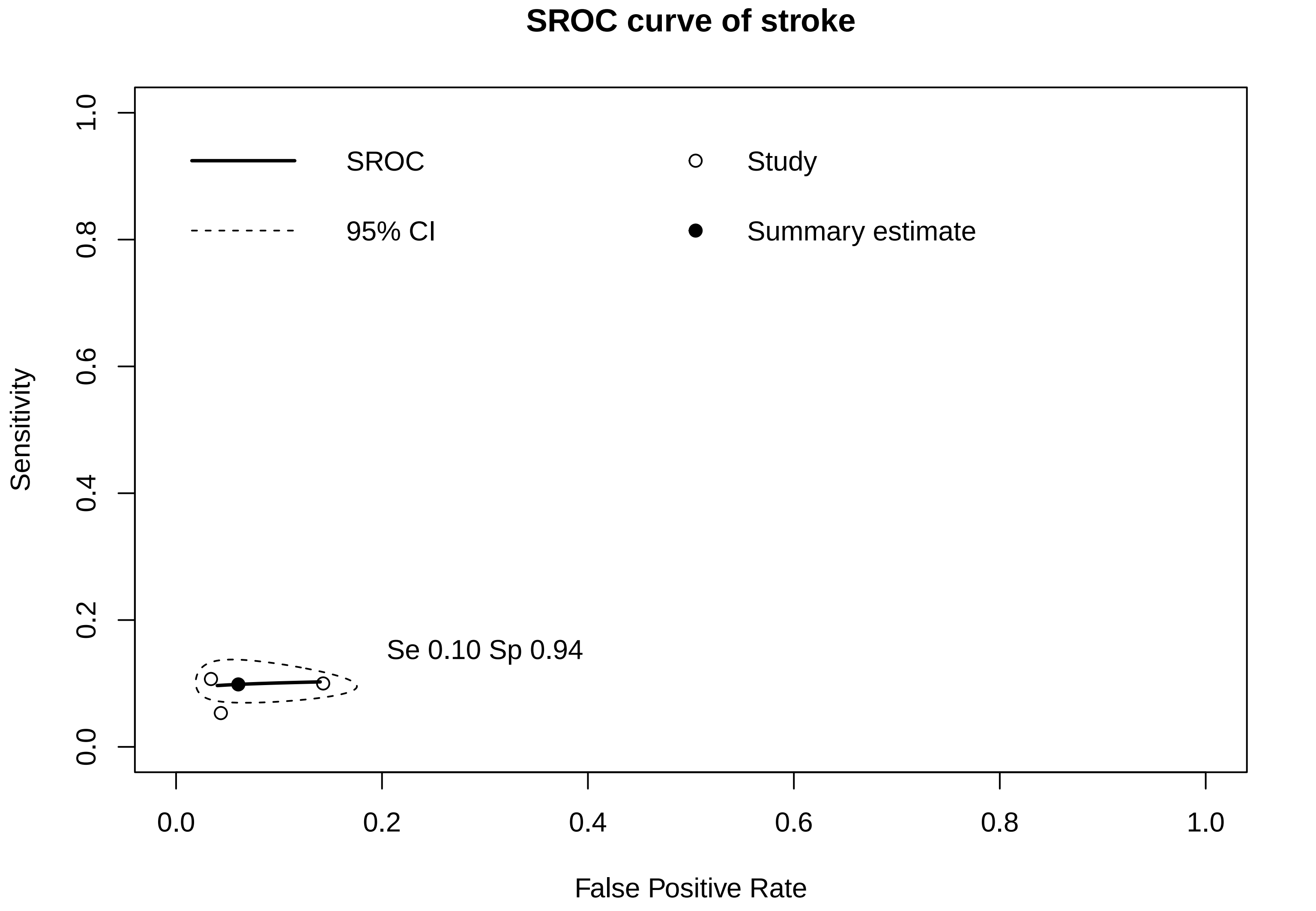

SROC curve of stroke
SROC
Study
95% CI
Summary estimate
Se 0.10 Sp 0.94
Sensitivity
0.0
0.2
0.4
0.6
0.8
1.0
False Positive Rate

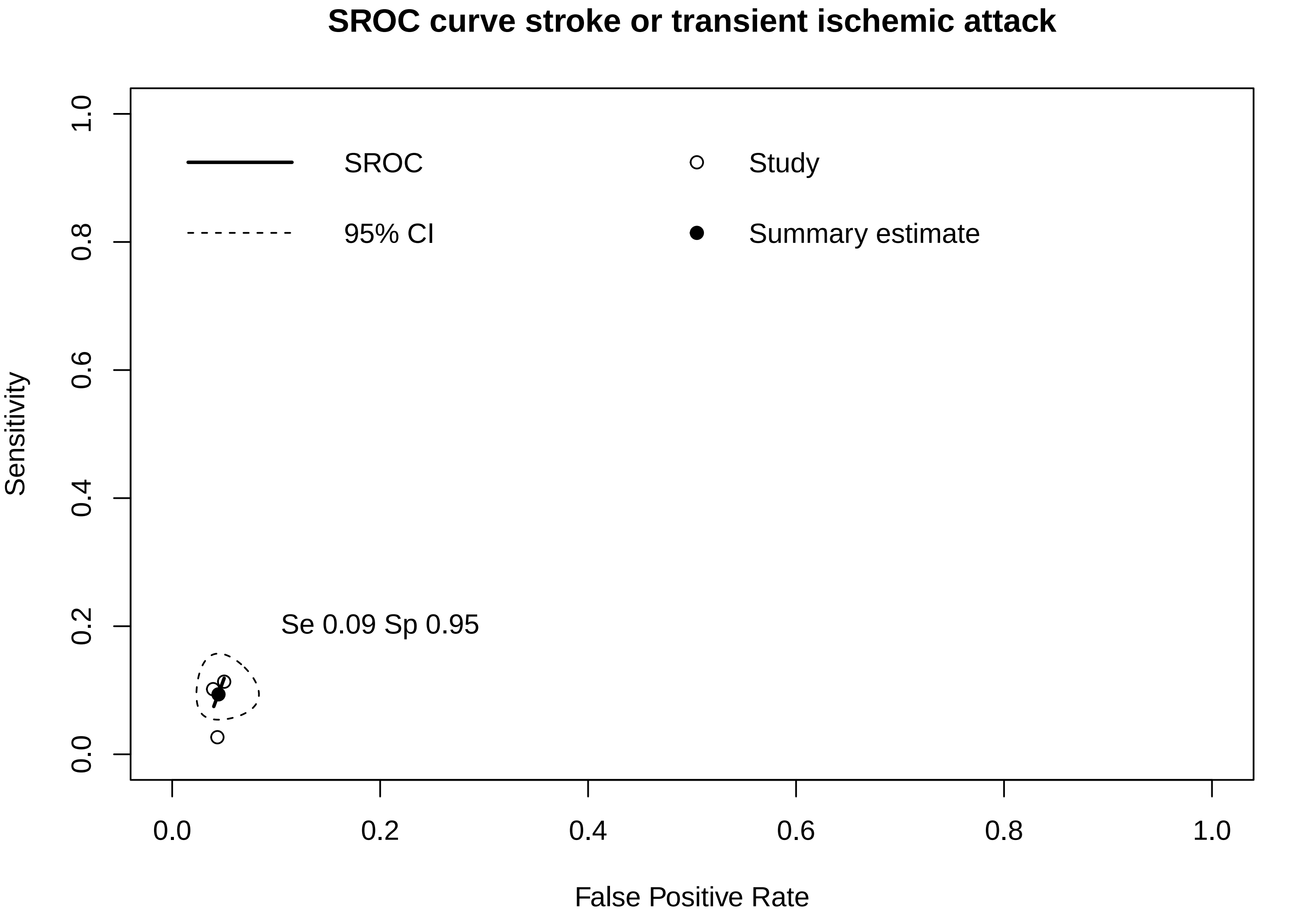

SROC curve stroke or transient ischemic attack
SROC
95% CI
Study
Summary estimate
Se 0.09 Sp 0.95
Sensitivity
False Positive Rate
0.0
0.2
0.4
0.6
0.8
1.0

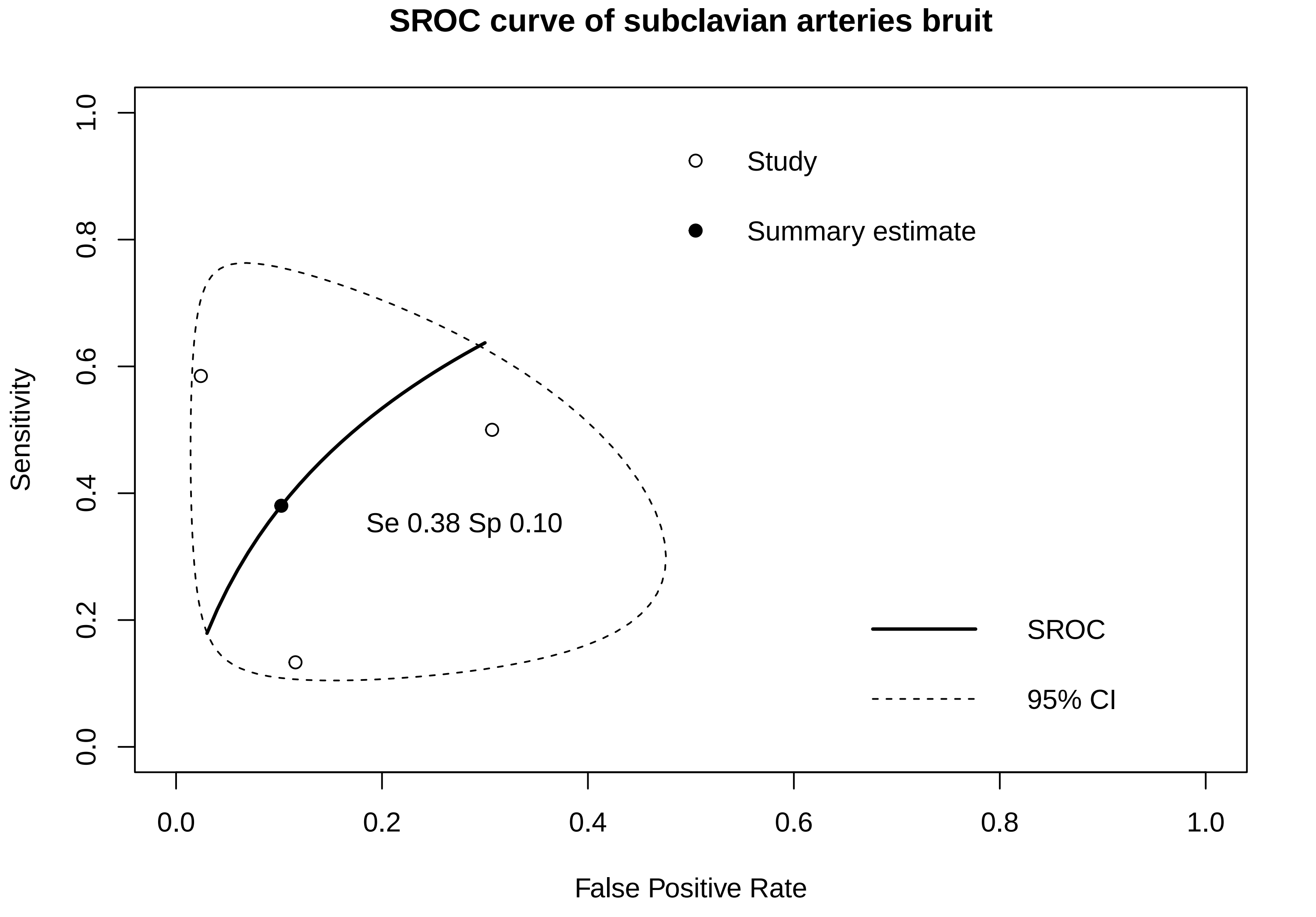

SROC curve of subclavian arteries bruit
Study
Summary estimate
Se 0.38 Sp 0.10
SROC
95% CI
Sensitivity
False Positive Rate
0.0
0.2
0.4
0.6
0.8
1.0

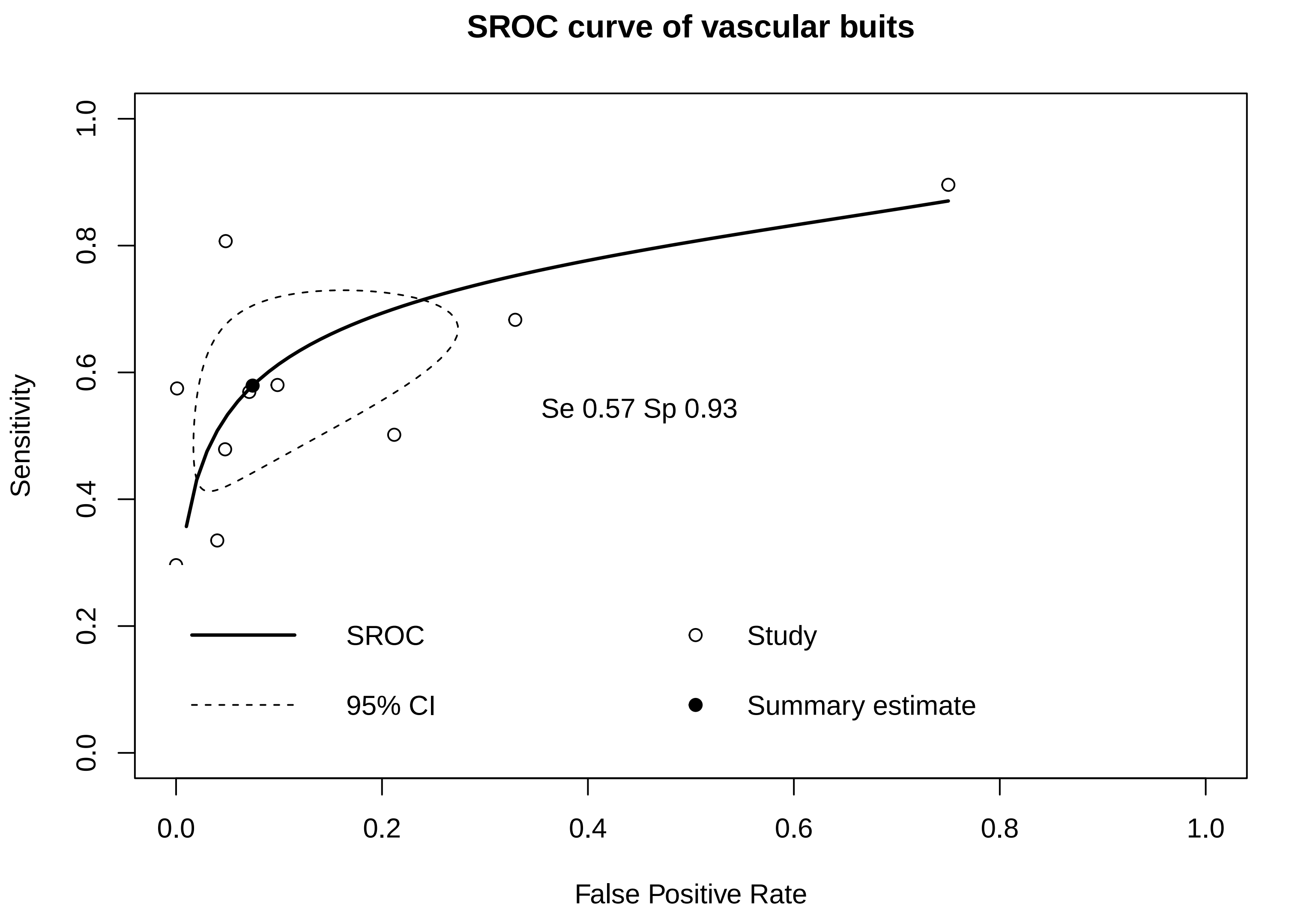
SROC curve of vascular buits
Sensitivity
False Positive Rate
0.0
0.2
0.4
0.6
0.8
1.0
Se 0.57 Sp 0.93
SROC
95% CI
Study
Summary estimate

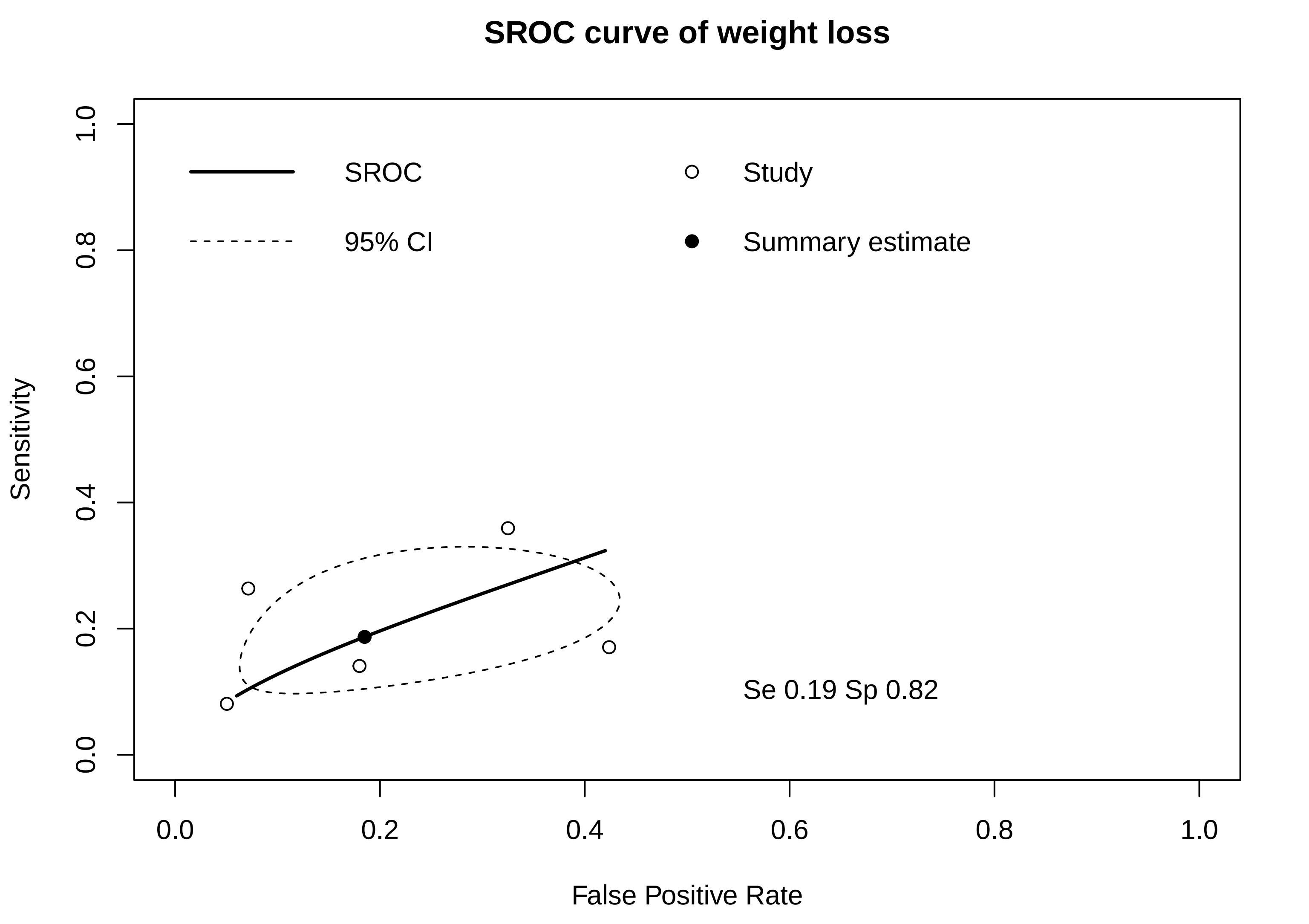
SROC curve of weight loss
SROC
95% CI
Study
Summary estimate
Se 0.19 Sp 0.82
Sensitivity
False Positive Rate
0.0
0.2
0.4
0.6
0.8
1.0
